# Earth orbital rhythms links timing of Deccan trap volcanism phases and global climate change

**DOI:** 10.1126/sciadv.adr8584

**Published:** 2025-03-07

**Authors:** Thomas Westerhold, Edoardo Dallanave, Donald Penman, Blair Schoene, Ursula Röhl, Nikolaus Gussone, Junichiro Kuroda

**Affiliations:** ^1^ MARUM - Center for Marine Environmental Sciences, University of Bremen, 28359 Bremen, Germany.; ^2^ Faculty of Geosciences, University of Bremen, Bremen, Germany.; ^3^ Department of Earth Sciences “Ardito Desio”, University of Milan, 20133 Milan, Italy.; ^4^ Department of Geosciences, Utah State University, Logan, UT 84322, USA.; ^5^ Department of Geosciences, Princeton University, Princeton NJ 08544, USA.; ^6^ Institut für Mineralogie, Universität Münster, 48149 Münster, Germany.; ^7^ Atmosphere and Ocean Research Institute, University of Tokyo, Kashiwa, Chiba 277-8564, Japan.

## Abstract

At the end of the Cretaceous, the massive Deccan trap (DT) volcanic eruptions are regarded as the primary driver of global climate deterioration. Accurate age models are key to unravel the sequence of events related to DT volcanism onset and effects on the global climate system. We establish a direct geochemical link between DT volcanism as recorded in marine osmium isotopic data and global climate change documented in benthic foraminifera carbon and oxygen isotope records. Based on our state-of-the-art astronomically calibrated age model, two major shifts in marine ^187^Os/^188^Os at 66.49 and 66.28 million years ago are contemporaneous with major eruption phases of the DT and disruptions of the global carbon cycle. Geochemical records and modeling suggest larger erupted volumes with high volatile emissions for the early phase of DT volcanism and point to differing emissions of SO_2_ and CO_2_ during the observed marine osmium shifts with diverse effects on the global climate system.

## Teaser

Different phases in end of the Cretaceous massive flood basalt volcanism had a differential impact on the global climate system.

## INTRODUCTION

On 10 thousand year (kyr)– to million-year timescales, climate dynamics on the Earth’s surface are driven by both external and internal processes. Subtle, quasiperiodic changes in Earth’s orbit around the sun are caused by gravitational interactions of larger celestial bodies in the solar system (*1*). This external or exogenic forcing is known as the orbital pacing of the climate system, and it regulates the amount of incoming solar radiation on the planet’s surface as well as its distribution across latitude, affecting the length and intensity of the seasons. Geological records show that global-scale variations in temperature and carbon cycle, the most fundamental cycle for life on our planet, are dominated by these orbitally paced changes with periodicities of ~20, ~40, and ~100 kyr. Stable carbon and oxygen isotope data, expressed as δ^13^C and δ^18^O, from carbonate tests of benthic foraminifera, which are unicellular organisms living on the sea floor of the ocean basins, provide a proxy record for past changes in global temperature and carbon cycle (*2*). However, Cenozoic data show that the Earth climate system does not respond in a linear way to the external orbital forcing. The response is complex and depends on the climatic boundary conditions, which are set by internal or endogenic processes driving plate tectonic motion. Plate tectonics shape the continental geography and topography, steer oceanic gateway locations and bathymetry, and influence climate dynamics mainly by the concentrations of atmospheric greenhouse gases, finely balanced between volcanic CO_2_ outgassing, sequestration by weathering of minerals as well as fossil organic carbon on land, and deposition as marine carbonates as well as organic matter (*3*). The interplay of external and internal forcing results in the long-term trends and short-term rhythmic variations observed in δ^13^C and δ^18^O records (*2*, *4*).

Disruption of climate dynamics by Large Igneous Province (LIP) flood basalt volcanism is proposed to have led to most of the mass extinctions in Earth’s history (*5*–*7*). The most recent extinction at the end of the Cretaceous, however, coincides with both LIP volcanism and a meteorite impact. Recent advances in radioisotopic dating of the voluminous Deccan trap (DT) flood basalt volcanism in India and the Chicxulub large meteorite impact crater in Mexico have unraveled the marked sequence of events that the living world had to face in the late Maastrichtian (*8*–*15*). A high-resolution benthic foraminifer stable isotope record in combination with an astronomically calibrated age model from the South Atlantic Ocean Drilling Program (ODP) Site 1262 shows that the onset of the main phase of DT volcanism correlates with deep-sea warming and a perturbation of the carbon cycle (*16*). However, a direct link between late Maastrichtian warming (LMW) to the different phases of the DT volcanism is needed to test causality arguments of the observed environmental deterioration before the meteorite impact at the Cretaceous/Paleogene (K/Pg) boundary [66 million years ago (Ma)].

Some marine sedimentary records contain geochemical fingerprints of flood basalt eruptions in osmium (Os) isotope data (*17*, *18*), because weathering of unradiogenic basalts can substantially affect ocean ^187^Os/^188^Os, which is then incorporated into marine carbonates. Late Maastrichtian ^187^Os/^188^Os records suggest a clear separation of the K/Pg meteorite impact and the onset of the main DT eruptions phase just before the C30n/C29r magnetic polarity chron reversal (*19*–*21*), consistent with the latest radioisotopic dating of DT volcanism (*13*, *14*). These Os isotope records were generated at low resolution in Deep Sea Drilling Project (DSDP) Hole 525A on Walvis Ridge in the Southeastern Atlantic and DSDP Site 577 from the Shatsky Rise in the Northeastern Pacific. To increase the resolution, additional data were generated for Site 577 as well as records generated at DSDP Site 690 from Maud Rise in the Southern Ocean and the Bottaccione land Section in Italy (*21*). These records reconfirmed that ^187^Os/^188^Os can be used to somehow constrain the onset of Deccan volcanism, but they could not disentangle the sequence of events because of the lack of an accurate age model for the sedimentary record. Nevertheless, an astronomically calibrated record of mercury (Hg) anomalies from Tunisia (*12*) advocates a tight correlation between eruptions (*11*, *14*) and global climate events. If such a direct link exists, then a geochemical fingerprint should be recorded in the marine ^187^Os/^188^Os as well as carbonate δ^13^C and δ^18^O data if placed on a common time frame by a very precise and accurate age model.

Here, we investigated new and published data from DSDP Site 577 and ODP Site 1209, in the Pacific Ocean, as well as DSDP Site 525 and ODP Site 1262 in the South Atlantic Ocean by using astronomically calibrated and cyclostratigraphic age models. We generated x-ray fluorescence (XRF) core scanning data for all cores to correlate records between holes and sites. We produced high-resolution benthic δ^13^C and δ^18^O data to document temperature and carbon cycle changes at ODP Site 1209. We generated magnetostratigraphic data to precisely define the position of the C30n/C29r chron boundary in these deep marine records. Last, we analyzed Os isotopes at Site 1209 and Site 1262, as well as synchronized the Tunisian Elles section (*12*) with our age models.

## RESULTS

**Fig. 1.**
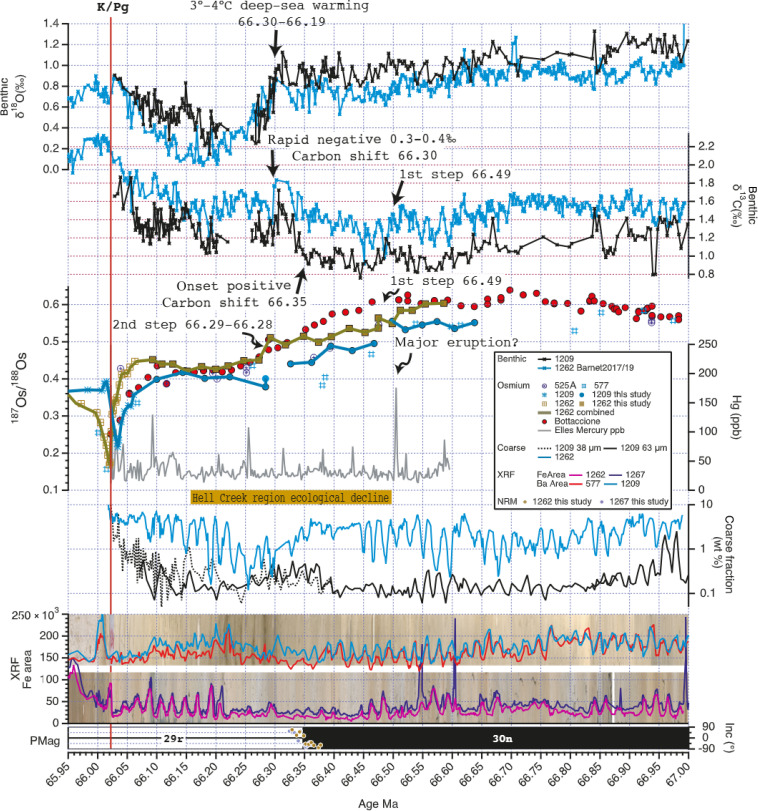
Late Maastrichtian marine geochemical records from Atlantic and Pacific Ocean on a highly accurate astronomically calibrated age model show evidence for and the sequence of events related to the onset of the Deccan trap (DT) flood basalt volcanic activity. Most of the shown data are derived from marine sediment records recovered in the South Atlantic (Walvis Ridge, Sites 525, 1262, and 1267) and the western Equatorial Pacific (Shatsky Rise, Sites 577 and 1209). The mercury (Hg) concentration record (*12*), however, is from the Tunisian Elles land section. Benthic foraminifer stable carbon and oxygen isotopes, representative of changes in the global carbon cycle and deep-sea temperature, reveal major synchronous shifts/steps in the marine Os isotope data and peaks in Hg concentration, suggesting a causal relationship to massive volcanic eruptions. Coarse fraction data indicate dissolution changes in the sedimentary records. Dominating cyclic variations in these data and semiquantitative elemental concentration data of iron and barium from XRF core scanning are related to astronomical cycles. The eccentricity-modulated precession cycles encountered in the data are the base for defining an astrochronological age model for the late Maastrichtian. Ecological decline in the hell Creek region from (*31*) and (*89*).

Integrating high-resolution paleoenvironmental data on a newly assembled astrochronology is key to document the sequence of events and assessing their causality during the past million years before the K/Pg boundary. XRF elemental intensity data obtained from all the cores of this study exhibit a pervasive imprint of orbital precession cycles (Fig. 1 and the Supplementary Materials). Late Maastrichtian records from around the world are well-known to be dominated by this orbital cycle (*22*–*24*), which, at about 67 Ma, had an average periodicity of ~20.4 kyr (*1*). To date, Maastrichtian deep-sea records from the Pacific and Atlantic Oceans have not been correlated at the single precession cycle level. Using the K/Pg boundary as a starting point and by simply counting 85 precession cycles back in time, a cyclostratigraphy was developed for each site. As a cross-check, we also have plotted the data using the existing astrochronology for the Site 1209 record based on stable 405-kyr–long eccentricity cycles, which results into an almost identical outcome compared to the age model based on precession cycle counting.

During the past million years of the Maastrichtian, all benthic δ^13^C and δ^18^O as well as coarse fraction data, representing changes in dissolution at the sea floor, and elemental XRF data show variations at the precession frequency (Fig. 1). From 67 Ma toward the K/Pg boundary at 66 Ma, Os isotopes show two marked decreases at 66.49 Ma and 66.29 to 66.28 Ma (tables S5 and S6), before dropping off (*19*, *21*) into the K/Pg boundary event. The first step at 66.49 Ma is associated with a major peak in Hg concentration in the Tunisian Elles record at 66.50 Ma and a negative 0.2 per mil (‰) shift in benthic δ^18^O from 66.50 to 66.45 Ma, indicating a maximum 1°C deep-sea temperature increase.

From 66.35 to 66.30 Ma, before the second ^187^Os/^188^Os drop just before the C30n/C29r reversal, benthic δ^13^C from both oceans shows a pronounced gradual shift toward higher values. At the same time, coarse fraction data start to rise in the Pacific sections and fall in the Atlantic records, pointing to a major change in deep-sea geochemistry. From 66.30 to 66.28 Ma, a very rapid 0.3‰ to 0.4‰ shift toward lighter δ^13^C values is synchronous with the second decreasing step in Os isotope data. Starting at 66.30 Ma and culminating at 66.19 Ma, the deep sea warmed by 3° to 4°C, marking the LMW event, hypothesized to be caused by DT volcanism (*16*, *25*). After the maximum warming at 66.19 Ma, benthic δ^18^O in both ocean basins recover slowly to pre-event levels just before the K/Pg boundary. Six dark-colored sediment intervals at ODP Site 1209 likely correspond to carbonate dissolution periods between 66.30 and 66.19 Ma where the absence of suitable benthic foraminifers caused small gaps in our δ^13^C and δ^18^O record. In the Elles section, this interval was characterized by high-stress conditions for benthic foraminifera (*12*).

## DISCUSSION

**Fig. 2.**
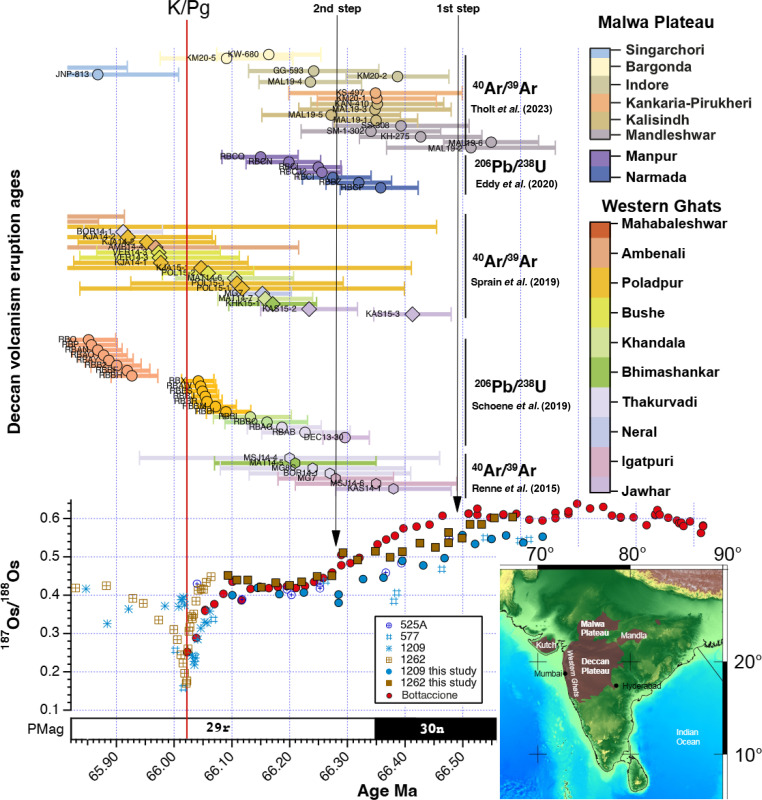
Deccan trap (DT) eruptive history and its fingerprint observed in ocean geochemistry. U/Pb and Ar/Ar ages of DT volcanic group from the Malwa Plateau and Western Ghats along marine sediment Os isotope record from the South Atlantic Walvis and the western Equatorial Pacific Shatsky rise. U/Pb ages are reported at the 2-sigma level without systematic uncertainties and refined by a stratigraphic Bayesian model after (*14*). Ar/Ar ages are displayed with a 2-sigma age uncertainty including analytical sources and calibrated after the Fish Canyon sanidine (FC) 28.294-Ma age (*90*). Systematic sources of uncertainty in both dating schemes are discussed in the Supplementary Materials.

Studies of changes in marine sediment Os isotope records around the K/Pg boundary have suggested that they have the potential to function as a proxy indicator of LIP volcanism (*21*). Comparing the two major steps in the synchronized Os isotope records from Atlantic ODP Site 1262 and Pacific ODP Site 1209 with the latest radioisotopic dated DT eruptions (Fig. 2 and table S7) (*10*, *11*, *13*–*15*) provides compelling evidence of a direct linkage between large-scale volcanic activity and global changes in climate. The first step at 66.49 Ma occurred before the C30n/C29r reversal and long before the proposed onset of major DT volcanism at 66.288 Ma (*14*), which correlates to the second step in Os isotopes and the rapid negative shift in benthic δ^13^C at 66.28 Ma. A high peak in the Hg data from the Elles section coinciding with the first Os isotope step suggests that the first major eruptions occurred substantially before the previously proposed onset. Dated flows beneath the C30n/C29r reversal do point to eruptions as early as 66.358 Ma (*11*). A U/Pb zircon date from a red bole between basalt flows in the Mandu Ghat section, belonging to the lower-middle Narmada Formation of the Malwa Plateau, erupted within the C30n magnetic polarity chron (*11*, *14*, *26*) and postdates the first Os isotopes step. Because the lowest dated basalts are not the lowest basalts in those sections (*11*), volcanism likely began before 66.358 ± 0.06 Ma. The Malwa Plateau, at the northernmost margin of the Deccan volcanic province (Fig. 2), has received less attention than the Western Ghats basalts, and its original volume was likely much greater than is presently preserved (*11*, *26*). At Malwa Plateau, the highest basaltic eruption rates occur ~350 kyr before the K/Pg boundary and at least start ~400 kyr before Western Ghats basalts (*11*, *27*). Thus, the minor warming of ~1°C in benthic δ^18^O along with a moderate δ^13^C shift at 66.49 Ma might be related to climatically relevant emissions of greenhouse gases.

More important DT activity correlates with the second step in Os isotope data at 66.28 Ma. At least, 15 dated major eruptions located in the Malwa Plateau Narmada and Manpur Formations and the lower Western Ghats Jawhar to Bhimashankar Formations cluster between 66.3 and 66.2 Ma. The rapid step in marine Os isotopes and benthic δ^13^C suggests that larger amounts of weatherable basalt must have been present at that time. If most of the basalt units were emplaced in a rather short period of time, then part of the negative δ^13^C shift could be caused by the cooking of organic-rich sedimentary rocks of the Narmada-Tapti rift basin (*11*).

An important feature of our benthic δ^13^C record is the positive shift in both the Pacific and Atlantic Ocean records from 66.35 to 66.30 Ma. Relating this increase in benthic δ^13^C to an interval of intensified DT volcanism is counterintuitive, as LIP volcanism is usually associated with negative δ^13^C excursions due to the negative δ^13^C (~−5‰) of most volcanogenic CO_2_. However, experiments using the Long-term Ocean-Sediment CArbon Reservoir (LOSCAR) model (see the Supplementary Materials) demonstrate that sulfur-heavy volcanic emissions can drive a positive δ^13^C shift in the deep ocean. Volcanogenic sulfur erupted as SO_2_ can readily oxidize to sulfuric acid in the atmosphere and rain out into the ocean. Sulfuric acid addition affects seawater carbonate chemistry in a somewhat different manner than CO_2_ (or carbonic acid) addition, but the effects are in many ways the same. Whereas CO_2_ increases seawater total dissolved inorganic carbon concentration ([DIC]) without directly affecting alkalinity, sulfur addition lowers the total alkalinity of seawater without immediately affecting [DIC]. Either way, this lowers seawater carbonate mineral saturation states, driving dissolution of seafloor carbonate and/or less burial of new carbonate, increasing the ocean’s total DIC inventory. With higher total [DIC], the biological pump—the flux of organic, light δ^13^C carbon from the surface to the deep ocean—has a smaller effect on the surface-to-deep δ^13^C gradient. In the case of CO_2_-rich emissions, the addition of low-δ^13^C CO_2_ overwhelms the DIC-increase effect and both surface and deep δ^13^C decrease. But when only S is released, surface δ^13^C is nearly invariant, and the smaller surface-deep δ^13^C gradient under higher [DIC] results in higher benthic δ^13^C.

These simple experiments also reveal that S-rich volcanism leads to a notably smaller CO_2_ rise than equivalent C-rich volcanism, which is consistent with the observation that the positive benthic excursion is not associated with a coincident warming in benthic δ^18^O. We, therefore, propose that the apparently global benthic foraminifera δ^13^C increase at 66.35 Ma represents an especially sulfur-rich phase of DT volcanism. Lavas from the Jawhar and Bushe Formations in the Western Ghats are reported to have much higher sulfur concentrations (*28*). According to the benthic records warming happened afterward, along with a δ^13^C decrease. It seems plausible that an initial pulse of volcanism at 66.35 Ma with high sulfur content was followed by a different volcanic eruption period at 66.28 Ma, characterized by high carbon outgassing or basalt intruding into carbon-rich host rock. The latter is supported by high-precision zircon ages of two silicic-alkaline intrusive complexes at the northwest edge of the DT and the Narmadi-Tapti Lineament (*29*). Also of interest is the observation that global benthic foraminifera δ^13^C start to shift toward heavier values at 66.07 Ma being synchronous with the Bushe Formation sulfur-rich lavas (*30*) just before the K/Pg boundary when Os isotope data start to shift as well. Both observations indicate that major phases in the DT volcanism start with sulfur-rich eruptions, stressing the ecosystem locally (*30*) and, possibly, more globally (*31*).

Last, we used a simple box model of the marine Os cycle (see the Supplementary Materials) to estimate how much DT volcanism would be required to drive the two late Maastrichtian shifts in ^187^Os/^188^Os observed in our records. The lack of narrow constraints on both the concentration and isotopic composition of Os in DT basalt (*21*, *32*) propagates into notable uncertainties in eruptive volume. Nevertheless, using reasonable assumptions for Os concentration (60 to 90 parts per thousand) and isotopic composition, our model requires weathering of 1.2 × 10^5^ to 1.8 × 10^5^ km^3^ and 4.4 × 10^5^ to 6.6 × 10^5^ km^3^ basalt of DT to explain the first (66.49 Ma) and second (66.28 Ma) ^187^Os/^188^Os shifts, respectively. These modeling results support our assertion that a larger proportion of the total extrusive DT component must have erupted earlier than previously thought (*11*), well before the K/Pg boundary, providing enough weatherable basalt to have an impact on the marine Os cycle. But because the basalts are placed in the lowermost part of the DT volcanic sequence, it is difficult at the moment to assess whether this amount of Os was supplied by the weathering of the basalts. Other possibilities may be considered to explain the supply of unradiogenic Os from DT to the ocean, such as releasing Os in a volatile phase, or via sulfide minerals. For example, the aged Archean rocks with highly radiogenic Os were widely covered by young basalts in the Indian Craton when the DT was formed, which may also have contributed to the decrease in marine Os isotope ratio (*19*).

## MATERIALS AND METHODS

### Marine sediment material from Scientific Ocean Drilling

In this study, we combined published and geochemical data generated on sediment cores drilled on the western Equatorial Pacific Shatsky Rise during DSDP Leg 86 [Site 577; (*33*)] and ODP Leg 198 [Sites 1209 and 1210; (*34*)], drilled on the eastern South Atlantic Walvis Ridge by DSDP Leg 74 [Sites 525, 527, and 528; (*35*)] and ODP Leg 208 [Sites 1262 and 1267; (*36*)] (fig. S1). To correlate the data in depth and time, the records were initially studied regionally and then globally using new high-resolution geochemical data, particularly high-resolution XRF core scanning and bulk stable carbon isotope data.

### Nondestructive XRF core scanning

Due to the high signal-to-noise ratio of nondestructive XRF core scanning data, they are perfect to perform testing of composite records, integrating data from multiple holes and sites by high-resolution correlation and cyclostratigraphic or astronomical age model development (*37*–*41*). To combine Walvis Ridge sites, we not only used published data from ODP Sites 1262 and 1267 (*38*) but also generated XRF data for cores from DSDP 525A-39R to 45R, DSDP 527*-30R to 34R, DSDP 528*-28R to 36R, ODP 1262-C14H, and ODP 1267B-36X.

For cores of DSDP Sites 525, 527, and 528, the XRF Core Scanner data were collected every 2 cm down-core over a 0.6-cm^2^ area with down-core slit size of 5 mm using generator settings of 10 kV, a current of 300 μA, and a sampling time of 20 s directly at the split-core surface of the archive half with XRF Core Scanner III (AVAATECH Serial No. 12) at MARUM Center for Marine Environmental Sciences, University of Bremen. The split-core surface was covered with a 4-μm–thin SPEXCerti Prep Ultralene1 foil to avoid contamination of the XRF measurement unit and desiccation of the sediment. The here reported data have been acquired by a X-PIPS detector from Canberra Industries Inc. (Areva Group; Model SXD 15C-150-500, with 150-eV x-ray resolution), the Canberra Digital Spectrum Analyzer DAS 1000, and an Oxford Instruments 100 W Neptune X-ray tube with rhodium (Rh) target material. Raw data spectra were processed by the analysis of x-ray spectra by Iterative Least square software (WIN AXIL) package from Canberra Eurisys.

For ODP Site 1262 and 1267 cores, the XRF Core Scanner data were collected every 2 cm down-core over a 1.2-cm^2^ area with down-core slit size of 10 mm using generator settings of 10 kV, a current of 35 μA, and a sampling time of 10 s directly at the split-core surface of the archive half with XRF Core Scanner III (AVAATECH Serial No. 12) at MARUM - Center for Marine Environmental Sciences, University of Bremen. The split-core surface was covered with a 4-μm-thin SPEXCerti Prep Ultralene1 foil to avoid contamination of the XRF measurement unit and desiccation of the sediment. The here reported data have been acquired by a SGX Sensortech Silicon Drift Detector (Model SiriusSD D65133Be-INF with 133-eV x-ray resolution), the Topaz-X High- Resolution Digital MCA, and an Oxford Instruments 100 W Neptune X-Ray tube with Rh target material. Raw data spectra were processed by the analysis of x-ray spectra by iterative least square software (WIN AXIL) package from Canberra Eurisys.

For DSDP Site 577 as well as ODP Sites 1209 and 1210, XRF data were collected every 2 cm down-core using XRF core scanner Avaatech serial no. 17 from the XRF core scanning facility located in at the International Ocean Discovery Program (IODP) Gulf Coast Repository at Texas A&M University (College Station, USA). The XRF scanning data for Sites 1209 and 1210 are published in (*42*). Data were collected over a 1.2-cm^2^ area with a down-core slit size of 10 mm using generator settings of 50 kV and a current of 0.75 mA, ideally for detecting Ba, and a sampling time of 12 s in each run directly at the split-core surface of the archive half. The split-core surface was covered with a 4-μm-thin SPEXCerti Prep Ultralene foil to avoid contamination of the XRF detector prism and desiccation of the cores. The data were acquired with a SiriusSD 65-mm^2^ Silicon Drift Detector Model 878-0616B - SGX Sensortech with 133-eV x-ray resolution at 5.9 keV and 3 kilo counts per second (kcps), the Canberra digital spectrum analyzer DAS 1000, and an Oxford Instruments 50-W Neptune X-ray tube with a Rh target. Raw data spectra were processed using the analysis of x-ray spectra by iterative least square software (bAxil) package from Canberra Eurisys. Because the sediment is rich in carbonate (mostly >96 wt %), measurements of other major and minor elements such as silicon (Si), aluminum (Al), titanium (Ti), manganese (Mn), and magnesium (Mg) were not done. Furthermore, Fe area data are unreliable when measured under 50 kV that is ideal for Ba. All XRF data used in this study are compiled in data S1 and available at the open-access Data Publisher for Earth & Environmental Science PANGAEA.

### Bulk and benthic stable carbon and oxygen isotope data

Published bulk and benthic stable isotope data were compiled for DSDP Sites 525 (*43*) and 577 (*44*–*46*), as well as for ODP Sites 1209 (*47*–*49*) and 1210 (*47*, *50*–*52*) [also published in (*42*)], 1262 (*16*, *53*, *54*), and 1267 (*47*, *55*). Benthic foraminifer carbonate δ^13^C and δ^18^O were measured on 404 samples at MARUM - Center for Marine Environmental Sciences, University of Bremen. Samples were oven dried over night at <50°C, then washed over a 63-μm sieve, and, again, dried over night at <50°C. Good to moderately preserved specimen of benthic foraminifera *Nuttallides truempyi* from the >150-μm fraction were analyzed. Analysis at MARUM was performed on Finnigan MAT 252 with Kiel III or ThermoFisher Scientific 253plus with Kiel IV automated carbonate preparation line. Samples were reacted with orthophosphoric acid at 75°C. Analytical precision based on replicate analyses of in-house standard (Solnhofener Limestone) averages 0.03 and 0.05‰ (1σ) for δ^13^C and d^18^O, respectively. Data are reported relative to the Vienna Pee Dee Belemnite international standard, determined via adjustment to calibrated in-house standards and NBS-19. All bulk and benthic stable isotope data used in this study are compiled in data S2 and available at the open-access Data Publisher for Earth & Environmental Science PANGAEA.

### Os isotope data

First-published Os isotope data were compiled for Sites 525 (*19*), 577 (*19*, *21*), 1209 (*56*), and 1262 (*56*). For this study, we generated Os isotope data at Site 1209 (18 samples) and Site 1262 (23 samples). We extracted Re and Os from sediment samples using inverse aqua regia digestion. The detailed method for the extraction and separation of Re and Os is described by Tejada *et al.* (*57*) and Kuroda *et al.* (*58*). After spiking of ^190^Os and ^185^Re, 0.3 to 1.5 g of powdered sample was sealed in a Carius tube (*59*) with 4 ml of inverse aqua regia and heated at 240°C for 24 hours. Os was then separated from the leachate using CCl4 (*60*, *61*) and further purified using the microdistillation method [modified after (*62*)]. Re was separated using a Bio-Rad AG1-X8 anion exchange resin (100 to 200 mesh).

Abundances and isotopic compositions of Os were analyzed by negative thermal ionization mass spectrometry (Thermo Fisher Scientific TRITON) at Japan Agency for Marine-Earth Science and Technology (JAMSTEC), and abundances of Re were measured by an inductively coupled plasma-quadrupole mass spectrometer (Thermo Fisher Scientific iCAP-Q) at JAMSTEC. Typical total procedural blanks for Re and Os were 0.2 to 3 and 0.3 to 1.6 pg, respectively, with an average ^187^Os/^188^Os value of 0.16 ± 0.03 (*n* = 6). All data were corrected for the procedural blank for each analytical batch. Instrument reproducibility was monitored on the basis of replicate analyses of the synthetic standard. Initial Os isotopic compositions (^187^Os/^188^Os_i_) were calculated for the time of deposition on the basis of the measured ^187^Os/^188^Os and ^187^Re/^188^Os values, the age of sediment, and the ^187^Re decay constant of 1.666 (± 0.017) × 10^−11^ year^−1^ (*63*). All Os isotope data used in this study are compiled in data S3 and available at the open-access Data Publisher for Earth & Environmental Science PANGAEA.

### Correlation and integration of marine drill cores

The first step to synchronize the records down to the single precession cycle level required high-resolution XRF data on sediments from multiple holes at each site. For this study generated and published XRF data were then used to test and refine the composite records of DSDP Site 577 (fig. S2), ODP Site 1209 (fig. S3A), and ODP Site 1210 (fig. S3B). For Site 577, some adjustments to the composite record had to be made. All relevant tables are given in data S4. For Site 1209, adjustments to the composite had to be made in the Maastrichtian. All relevant tables are given in data S5, which is published in (*42*). No correction was necessary for Site 1210 data. After the composite checks, XRF barium (Ba) records for ODP Sites 1209 and 1210 (fig. S4), as well as DSDP Site 577 and ODP Site 1209 (fig. S5), were correlated to each other forming an integrated record for the Pacific Shatsky Rise with Site 1209 as the depth reference. To form a combined Atlantic Walvis Ridge late Maastrichtian XRF iron (Fe) record, data from DSDP Sites 525, 527, and 528, as well as ODP Sites 1262 and 1267 (fig. S6), were correlated. Here, Site 1267 served as the depth reference. The correlation tie points are listed in data S7 and allow a detailed correlation on the single-cycle level.

### Data compilation for Walvis Ridge (South Atlantic) and Shatsky Rise (Northwest Pacific)

We compiled published data (*16*, *19*, *21*, *25*, *36*, *38*, *43*–*54*, *56*, *64*–*68*) with data generated for this study each for Walvis Ridge (fig. S7) and Shatsky Rise (fig. S8). Compiled data types are magnetostratigraphic parameters, XRF core scanning elemental data, osmium ^187^Os/^188^Os isotope data, benthic foraminifer, and bulk carbonate stable carbon and oxygen isotope data. Data that were not available in a data repository or from the publication itself have been digitized from graphs in the respective publication. We are aware that this introduced some uncertainty; however, this did not affect any conclusion of this study. Digitized data are provided for Chave (*65*) (magnetostratigraphy of Sites 525, 527, and 528) and Bleil (*67*) (magnetostratigraphy of Site 577). Data are reported in the figure on the depth of the reference Site 1209 for Shatsky Rise and Site 1267 for Walvis Ridge. The compilation of data reaffirms the high quality of the detailed correlation by showing clear trends and pattern even from multiple publications that were not possible in previous compilations.

For reproducibility and completeness, all data are part of the Supplementary Materials:

-Magnetostratigraphic data: data S6

-XRF core scanning elemental data: data S1

-Os isotope data: data S3

-Stable carbon and oxygen isotope data: data S2

### Correlation Walvis Ridge to Shatsky Rise—Phase relationships

A key task in our study was to correlate the regional records of Shatsky Rise and Walvis Ridge on a, so far, unprecedented level. Records from both regions display orbital precession-related variations in multiple parameters. Identifying and counting the precession cycles in XRF core scanning iron and barium elemental data was the initial step. The second step was to correlate the counted cycles between the regions. To do so, the phase relationship of the individual cycles in XRF core scanning data and bulk stable isotope data was established. Assuming that bulk stable isotope variations are a global synchronous signal, the phase relationship of XRF core scanning data between different regions can be estimated. XRF barium maxima in the Site 1209 record occur when bulk carbon isotope data are heavier. XRF iron maxima in the Site 1267 record occur when bulk carbon isotope data are lighter. Thus, to correlate the Shatsky Rise to the Walvis Ridge records, Site 1267 XRF iron minima were correlated to Site 1209 XRF barium maxima (fig. S9). Starting in the earliest Paleocene, 111 tie points were established, as given in data S7, correlating the two regional records on the precession cycle level. The cycle counting for each regional base site is given in figure S10a for Shatsky Rise and in figure S10b for Walvis Ridge.

XRF data for barium from Site 1209 can be used as the signal for cyclostratigraphy as shown by Kim *et al.* (*42*). Over a few cycles in XRF Barium data, pelagic marine barite was extracted from discrete samples of the sediment, suggesting that XRF Barium records represent changes in export production in these tropical Pacific sediments. It was proposed that the primary production, as well as its recycling in the euphotic zone, was precession paced by changing water column stratification, upwelling intensity, and continental nutrient fluxes (*42*). Pelagic marine barite in deep-sea sediments correlate with organic carbon flux out of the photic zone (*69*, *70*), making these good proxies for export production.

### Astrochronology and cyclostratigraphy

The second key task for this study was to establish both an astrochronological and cyclostratigraphical age model for the records. The astronomical age model can be achieved by identifying cycles related to variations in Earth orbit in the records and tying those cycles to astronomical solutions of orbital cycles calculated for the 66- to 70-Ma time interval. Because of uncertainties in the initial conditions of the solar system and the chaotic behavior of the largest bodies in the asteroid belt orbital solutions for short eccentricity with a period of ~100 kyr, the main target for constructing astrochronologies in the early Paleogene (*2*) will never be precise beyond 60 Ma (*71*). However, to establish an astrochronology, the stable long eccentricity cycle with a period of ~405 kyr can be used (*1*, *72*). For avoiding circular reasoning and allowing to extract characteristic patterns of eccentricity embedded in the records, it was suggested to use a simple cosine function for intervals older than 60 Ma (*1*, *72*). An age model identifying the long eccentricity cycle in bulk carbonate carbon stable isotope data was recently established for Site 1209 for the interval from 62.5 to 71.28 Ma using this cosine function (*42*). The astrochronology for Site 1209 is given in table S1 and transferred to Walvis Ridge records through correlating Site 1209 and Site 1267 as described above.

Independent from the astrochronology, which can only provide an age tie point every 405 kyr, we also acquired a much higher resolved cyclostratigraphy using the cycle counting of the Walvis Ridge to Shatsky Rise precession cycle correlation (fig. S10, A and B). We started the cycle counting at the K/Pg boundary setting its age to 66.022 Ma (*73*) and then added 21 kyr for 80 consecutive precession cycles below (fig. S11). The cyclostratigraphic age model is given in table S2. The resulting sedimentation rates from the cyclostratigraphy are fluctuating around the sedimentation rates resulting from the astrochronology and average from 0.8 cm/kyr in the latest Maastrichtian to 1.2 cm/kyr at 280-m–composite depth Site 1209 (fig. S11). Sedimentation rates for both age models are similar from the K/Pg boundary to precession cycles 68 to 70 representing the interval from 66.022 to 67.45 Ma. In the older interval, sedimentation rates diverge with higher sedimentation rates in the cyclostratigraphic age model likely related to missing a precession cycle in the amplitude minimum around 276.5-m–composite depth Site1209. Because, for this study, the interval younger than 67 Ma is critical, no correction was implemented in the oldest part for now.

Both age models were than applied to the compiled data from both regions (figs. S12 and S13). In the main text of the study, we use the cyclostratigraphic age model because of its higher resolving character, allowing much more detailed age constrains. Nevertheless, both age models provide a very accurate timing of events for the late Maastrichtian period.

### Natural remanent magnetization and magnetostratigraphy

We collected a total of 32 oriented cube specimens from three different sites: 12 specimens at Site 528, 10 specimens at Site 1262, and 10 specimens at Site 1267. The sampling strategy was developed following the previous knowledge of the expected C30n/C29r chron boundary position. The cores stored at the Bremen Core Repository consist of calcareous ooze drilled in 1980 in the case of Site 528 (*74*) and in 2003 in the case of Sites 1262 (*75*) and 1267 (*76*). The long permanence in storage increased the stiffness of the sediments, minimizing the physical disturbance that can be induced by the sampling procedure. The samples were first cut with a nonmagnetic ceramic blade in 8-cm^3^ cubes from the working half of the cores and oriented following the right-hand standard IODP convention (*70*). Each sample was then inserted within standard 8-cm^3^ plastic cubes. The stratigraphic position of the specimens within each site is listed in table S3.

We investigated the vector components of the sediments natural remanent magnetization (NRM) by means of alternate field (AF) demagnetization. After measuring the initial NRM, we adopted 17 demagnetization steps from 3 mT to a maximum of 100 mT, measuring the NRM after each steps with a 2G-Enterprises SQUID magnetometer. We identified the characteristic remanent magnetization (ChRM) of each sample by means of vector end-point diagram visual inspection (*77*), using the PuffinPlot freeware (*78*). The selected demagnetization points are interpolated with principal components analysis (*73*), and, to avoid an artificially low maximum angular deviation, we did not force any of the ChRM directions to pass through the origin of the demagnetization axes, a procedure normally referred to as “anchoring” (*79*). Because the cores are not oriented with respect to the geographic North, the declination of the ChRM directions is not representative of the paleomagnetic field. We thus interpreted the magnetic polarity using only the inclination data, with the up-pointing ChRM directions (i.e., negative inclination) acquired during normal geomagnetic polarity field, while the down-pointing (i.e., positive) inclinations were acquired during reversed geomagnetic field.

To investigate the nature of the magnetic minerals, after NRM analysis, all specimens were used for anhysteretic remanent magnetization (ARM) acquisition, using a direct current bias field of 50 μT and 22 AF field steps ranging from 2 to 100 mT. After the last AF step of 100 mT, we demagnetized the peak ARM by applying the same AF steps used for the acquisition. This set of analyses was eventually followed by isothermal remanent magnetization (IRM) acquisition adopting 24 steps from ~10 to ~700 mT, measuring the induced remanence after each steps. This last analysis explores a much wider range of magnetic coercivity than the ARM-based analysis. We isolated the coercivity components of the IRM curves by using the MAX UnMix freeware (*80*), which is based on the skewed generalized Gaussian function theory of coercivity distribution (*81*). NRM demagnetization and rock-magnetic measurements were carried out at the paleomagnetic laboratory of the University of Bremen (Germany).

### Carbon cycle modeling using LOSCAR

To explore the effects of massive sulfur- and carbon-rich extrusive volcanism on marine δ^13^C records, we performed a suite of idealized experiments using the LOSCAR (version 2.0.4) model. The model was run to a stable equilibrium in its “Paleogene” configuration (including a Tethys ocean reservoir) with an equilibrium pCO_2_ of 1000 parts per million by volume (ppmv). Three scenarios of volcanic emissions were subsequently run: a CO_2_-only release of 10,000 giga tons of Carbon (GtC) to the atmosphere as CO_2_ (δ^13^C = −5‰), a SO_2_-only release of 10,000 GtS [which is implemented in the model as a reduction in surface ocean alkalinity, after (*82*) and (*47*)], and a scenario with both of those C and S releases combined. All volcanic emissions occur at a constant rate over 100 kyr.

### Os isotope data box model

We used a simple 1-box model of the marine Os cycle to estimate how much DT basaltic rocks would be required by weathering to drive the two late Maastrichtian shifts in ^187^Os/^188^Os observed in our records. The kinetic box model is basically similar to those provided by Tanaka *et al.* (*83*) and Sato *et al.* (*84*). The typical Os isotope ratio of seawater before 66.49 Ma was ~0.56. This value is considered to represent the background steady state before the DT formation. Under the steady state, the flux and isotopic ratio of Os transported by rivers from the upper continental crusts and the flux and isotopic ratio of Os supplied by cosmic dust from extraterrestrial sources are assumed to be the same as those at present and kept constant through time, i.e., the riverine input was 295 t of Os ky^−1^ and ^187^Os/^188^Os = 1.54 (*85*), and cosmic input was 17.64 t of Os ky^−1^ (*85*) and ^187^Os/^188^Os = 0.126 (*86*).

### Integration of the Tunisian Elles section

The Elles section in Tunisia is composed of marls, silty marls, shales, and clay stones deposited in 100- to 150-m paleodepth on the continental shelf of the Tethys seaway (*12*, *87*, *88*). It has a cyclostratigraphic age model based on precession cycle counting and, besides other geochemical data, a record of Hg concentration thought to potentially reflect a fingerprint of DT volcanic eruptions (*12*). We have adopted the cycle identification developed by Keller *et al.* (*12*) and correlated the Elles carbonate weight % record to the XRF barium record of Site 1209. Carbonate content maxima in the Elles section correspond to heavier benthic foraminifer stable carbon isotope values (fig. S14); at Site 1209 XRF barium, maxima correspond to heavier carbon isotope values. Thus, we correlated maxima in the Elles carbonate weight % to maxima in Site 1209 XRF barium. The tie points are given in the cycle counting age model (table S2).

## Supplementary Materials

### The PDF file includes:

Supplementary Text Figs. S1 to S21 Tables S1 to S7 Legends for data S1 to S7 References
