# Supplementary material for: Earth orbital rhythms links timing of Deccan trap volcanism phases and global climate change
Source: Sci Adv. 2025 Mar 7;11(10):eadr8584. doi: 10.1126/sciadv.adr8584 (PMC11887795; doi:10.1126/sciadv.adr8584)
Supplement: Supplementary file 1 — Supplementary Text Figs. S1 to S21 Tables S1 to S7 Legends for data S1 to S7 References [file sciadv.adr8584_sm.pdf]

Science Advances
AAAS

Supplementary Materials for

Earth orbital rhythms links timing of Deccan trap volcanism phases and global climate change

Thomas Westerhold *et al.*

Corresponding author: Thomas Westerhold, twesterhold@marum.de

Sci. Adv. **11**, eadr8584 (2025)
DOI: 10.1126/sciadv.adr8584

The PDF file includes:

Supplementary Text
Figs. S1 to S21
Tables S1 to S7
Legends for data S1 to S7
References

Other Supplementary Material for this manuscript includes the following:

Data S1 to S7

Supplementary Text

Carbon Cycle Modeling Results

Results from idealized LOSCAR C and S emissions experiments (atmospheric pCO_2, surface and deep carbonate saturation state and $\delta^{13}C$) are plotted in Figure S15. Both C and S emissions acidify the ocean and cause seawater carbonate saturation states to decrease, resulting in dissolution of carbonate on the seafloor and in the water column, driving higher seawater dissolved inorganic carbon concentrations. Under higher [DIC], the biological pump (the flux of isotopically light organic carbon from the surface to deep ocean) results in a smaller $\delta^{13}C$ gradient between the surface and deep oceans. This reduction in $\delta^{13}C$ gradient holds true across all 3 experiments, regardless of carbon vs. sulfur emissions, but whether that reduction is accomplished through changes in surface or deep $\delta^{13}C$ varies. In the CO_2-only experiment, this manifests as a lower surface $\delta^{13}C$ (due to the addition of volcanogenic CO_2 with $\delta^{13}C$ = -5 ‰) and nearly invariant deep $\delta^{13}C$. In the sulfur-only experiment, this manifests as nearly invariant surface $\delta^{13}C$ (as the higher DIC comes from dissolution of marine carbonates with a $\delta^{13}C$ near 0 ‰) and higher benthic $\delta^{13}C$. Simultaneous C and S emissions result in lower surface ocean $\delta^{13}C$ and higher benthic $\delta^{13}C$.

Geochronology compilation and reporting

Published U-Pb and Ar/Ar geochronology for Deccan Traps eruptions are reported here with 2-sigma internal uncertainties only, neglecting the uncertainties in decay constants and physical constants (*31*). We do so because the U-Pb date with only internal uncertainties (66.016±0.050 Ma, recalculated in (*14*) using data from Clyde et al. (*94*)) and the Ar/Ar date with only internal uncertainties (66.052±0.041 Ma) agree well at this level of comparison near the K-Pg boundary using the Fish Canyon sanidine age calibration at 28.294 Ma reported in Renne et al. (*14*). Additionally, in this paper the cyclostratigraphic age models are pinned to the K/Pg boundary astrochronology age of 66.022 Ma (*76*), consistent with the above U-Pb radioisotopic age for the K-Pg boundary, thereby absolving the need for systematic sources of uncertainty when comparing the radioisotopic dates to the cyclostratigraphic age models. The original publications with the geochronologic data report the dates with full systematic uncertainties in addition to those used here, so the reader is referred to those publications for cases where they are needed.

Osmium Box Model Results

As initial conditions, the total amount of Os in the ocean before the event was assumed to be 13,000 t Os, the same as today (*95*). This amount affects the time it takes to reach the next stable phase (steady state) when a change occurs in the model. In the modern ocean, total amount of Os is thought to be in the range between 7200 and 16000 t Os (*96*). Even if the initial amount of the Os in the ocean is varied in this range, model result is not significantly changed.

To obtain the pre-event $^{187}Os/^{188}Os$ value of 0.56 under these conditions, the Os supply flux from the mantle ($^{187}Os/^{188}Os$ = 0.126, (*97*)) at that time should be 543 t ky^{-1}. The additional Os flux from DT basalt ($^{187}Os/^{188}Os$ = 0.127, (*35*)) required to reduce the marine $^{187}Os/^{188}Os$ value from 0.56 to 0.49 after 66.49 Ma (the first decrease) would be 156 t Os ky^{-1} (Fig. S16 and Table S4a). The total amount of Os supplied to the ocean during the period 66.49 Ma to 66.29 Ma was estimated to be at 3.15 x 10^4 t Os.

The Os flux from DT basalt required to cause the second decrease in the $^{187}Os/^{188}Os$ value to 0.41 after 66.28 Ma would be 429 t Os ky^{-1} (Fig. S16 and Table S4b). The total amount of unradiogenic Os released to the ocean during the period 66.28 to 66.02 Ma is estimated to be 1.14 x 10^5 t Os. Assuming an average Os concentration of 60-90 ppt and a typical density of 2.9 g cm^{-3} in the DT basalts, the estimated amounts of Os are supplied from DT basalt weathering in the order of 1.2-1.8 x 10^5 km^3 and 4.4-6.6 x 10^5 km^3 for the initial

and second declines, respectively, which are equivalent to 8-12% and 29-44% of the volume of the estimated DT basalt (1.5 x 10^6 km^3, Coffin and Eldholm (*98*)). In practice, however, these basalts are placed in the lowermost part of the DT volcanic sequence, making it difficult to assess whether this amount of Os was supplied by the weathering of the basalts. For both scenarios, we must assume weathering rates in the order of 10^{12} tons of basalt per kyr. This is significantly higher than the lab-based estimation of dissolution rate of silicate minerals that is in the order of ~10^{-8} mol s^{-1} m^{-2} at maximum (*99* and references therein). Although we understand that it is not simple enough to directly compare the lab-based dissolution rates with actual basalt weathering rates, the difference may be explained by the fact that juvenile basalts are more readily weathered compared with the other silicate rocks (*100, 101*).

There could be other possibilities that explain the supply of unradiogenic Os from DT to the ocean, such as releasing Os in a volatile phase, or via sulfide minerals. For example, the high amount of Os was detected in the volatile phase during the 1984 eruption of Mauna Loa, Hawaii (*102*). In addition, high concentrations of Os in the weathered residue (laterite) in the DT area may imply the presence of Os-rich phase in the original DT rocks (*103*). As Ravizza and Peucker-Ehrenbrink (*19*) suggested, there is a possibility that DT covered a wide area of aged (Archean) rocks on the Indian Craton, thereby reducing the supply of highly radiogenic Os to the ocean. In such case , the volume of basaltic rock to be weathered from DT should have been much smaller. More recently, (*104*) estimated the flux of Os from DT during the latest Maastrichtian to be 87-238 kg/yr (average 138 kg/yr), which is comparable with our estimation for the first drop of $^{187}Os/^{188}Os$ values (156 t Os ky^{-1}) but smaller than the second drop (429 t Os ky^{-1}). The difference would be attributed to the fact that Sinnesael et al. (*104*) took into account in their box model the cessation of the supply of radiogenic Os from the aged Indian cratonic rocks due to the covering by DT basalt. Although more work is needed to constrain the process how the emplacement of DT caused the gradual decrease in $^{187}Os/^{188}Os$ value from 0.6 to 0.4, marine Os isotope ratio should have started decreasing immediately when unradiogenic Os was released to the ocean given the relatively short residence time of Os in the ocean (10^3 to 10^4 years).

It should also be noted that an extremely high weathering rate (~five orders of magnitude higher than the lab-based dissolution rate of silicate minerals) is required to explain the major drop of $^{187}Os/^{188}Os$ value from 0.4 to less than 0.2 at K/Pg. Thus, it is unlikely to be caused by the weathering of DT basalt. From this perspective, it is most plausible to attribute the decrease in the Os isotopic ratio of the main to the Chicxulub meteorite impact, as has been previously interpreted (e.g., (*19*)).

The global warming caused by the inferred DT volcanism might have enhanced the rate of silicate weathering (e.g., (*105*)). Future development of a model that integrates the Os cycles with oxygen and carbon isotopic compositions, temperature records and other factors will improve the accuracy of the geochemical cycle simulation.

Natural Remanent Magnetization and Magnetostratigraphy Results

Rock Magnetism

The samples from all three sites are dominated by a low magnetic coercivity phase. This phase possesses an average $B_{1/2}$ (the field at which half of the saturation is reached) of 25 mT in the samples from Sites 1262 and 1267, while of 49 mT at Site 528 (Figure S17 A-D). This low coercivity phase is interpreted as magnetite (Fe_3O_4), which went through a more pervasive partial oxidized to maghemite (Υ-Fe_2O_3) in the case of the sediments from Site 528, likely due to the longer time spent in the aerobic storage conditions. Low-temperature oxidation of magnetite results in particles with an oxidized shell around a pristine core, with the consequence of a strong internal oxidation gradient that increases the magnetic coercivity of the magnetic grain (*106*). IRM curves are best interpolated adding also the minimal

contribution of a high coercivity magnetic phase, either hematite or goethite, which however do not contribute to the remanence, as revealed by analysis of the NRM vector components.

ARM analysis also shows similar coercivity difference between Sites 1262-1267 and Site 528. In case of non-interacting magnetic particles the remanence acquisition and demagnetization curves should be symmetrical, both in case of IRM (*107*) and ARM (*108*). ARM acquisition and demagnetization curves from the analyzed samples appear to be symmetrical (Figure S17 E). This can be quantified by the R parameter (*107*), determined by the ratio of the ARM at the crossing point over the maximum ARM of each curve, where R= 0.5 indicates non-interacting particles. Samples from the three sites all possess values around 0.46, suggesting negligible magnetic particles interaction (Figure S17 F). The crossing point between acquisition and demagnetization curves approximate the coercivity of remanence (Hcr), which is about 10 mT higher in the samples from Site 528 (Figure S17 F), in agreement with the higher $B_{1/2}$ observed in the IRM curves.

In synthesis, sediments from Sites 1262, 1267, and 528 are dominated by virtually non-interacting magnetite that went through partial low temperature oxidation (maghemite-coated magnetite) in Site 528, likely due to the longer exposure to the aerobic storage conditions.

Paleomagnetism

The intensity of the NRM is similar in the three sites, and ranges from a minimum of $3.4x10^{-3}$ A/m to a maximum of $5.1\ x10^{-2}$ A/m, with an average of $2.0x10^{-2}$ A/m. A viscous magnetic overprint was isolated in all samples between the initial measurement and an AF field ranging from six to a maximum of 20 mT. In Sites 1262 and 1267 this magnetic component is well-developed and oriented vertically downcore, parallel to the coring line (Figure S18 A-D), so it was very likely acquired during the drilling process (*109*). In site 528 the same overprint is less developed, and the vector end-points are rather grouped very close to the initial measurement, without defining a clear and interpretable vector (Figure S17 E-F). Despite this difference, all specimens from the three sites show the presence of a linear characteristic remanent magnetization (ChRM). This component was isolated within an AF interval ranging from a minimum of 6–20 mT to a maximum of 70–100 mT, with the maximum unblocking field lower than 70 mT only in three cases (Table S3). The ChRM directions are isolated by interpolating on average 13 vector end-points, with an average MAD of 2.77°, supporting the reliability of the dataset (Table S3).

In all three sites the inclination shifts from positive to negative values moving stratigraphically downward, which indicates a transition from reversed to normal paleomagnetic field from the youngest to the older sediments (Figure S19). This reversal is sharp and constrained between two consecutive samples in Hole 1262B and Hole 528X, while it is defined by four samples in Hole 1267B (Figure S19). This is not unusual, as sedimentary rocks often record transitional paleomagnetic directions across polarity reversals (*110*), or saw-tooth inclination pattern due mobilization of magnetic particles near the sediment-water interface before complete burial lock (see e.g. magnetostratigraphy of Dallanave et al. (*111*), particularly across Chron C27n).

Magnetostratigraphy

The main focus of the new NRM data was to locate the C30n to C29r reversal with high precision. Best results were obtained by samples from Site 1262 and 1267. Because those sites are less affected by coring disturbance then the other drill cores we defined the reversal location based on those data (Fig. S20 and S21). The C30n to C29r reversal is placed as given in the table PMag 1.

Table PMag 1: Position and ages for the C30n to C29r reversal from this study

Reversal	Top Sample	Bottom Sample	CycloAge	AstroAge
C30n(y)	1267B33X1, 88-90cm	1267B33X1, 105-107cm	66.346 Ma	66.328 Ma

Duration of C29r is not focus of this manuscript, but using the astronomically tuned age in CENOGRID (*2*) for the C29r/C29n boundary of 65.653 Ma and ages given above for the C30n/C29r boundary we can estimate the duration of Chron C29r. The cyclo age gives a duration of 693 kyr and the astro age a duration of 675 kyr. Sprain et al. (*26*) calculate the duration of Chron C29r based on 14 new magnetostratigraphic sections and 18 new $^{40}Ar/^{39}Ar$ tephra ages from the Hell Creek region, Montana, to be 587 ± 53 kyr (534-640 kyr). Sprain et al. (*26*) provides for the C30n/C29r boundary an age of 66.304 ± 0.054/0.069 Ma and for the C29r/C29n boundary an age of 65.708 ± 0.043/0.060 Ma. In comparison the astrochronological duration is a bit longer maybe related to lower sedimentation rates in the early Paleocene part of C29r in the used drill sites. The duration between the K/Pg boundary and the C29r/C30n boundary is according to Sprain et al. (*26*) between 183 and 321 kyr, consistent with the astrochronological estimate of 306 to 324 kyr. There are also age estimates in Schoene et al. (*14*) for each reversal, as recorded in the DT. With 384 ± 65 kyr they are broadly consistent with the duration estimate 306 to 324 kyr given here.

The Osmium isotope record from the Bottaccione Gorge (Gubbio, Italy)

A very detailed Osmium isotope record is available from the Bottaccione Gorge (Gubbio, Italy) published by Robinson et al. (*21*). The Bottaccione section has no published cyclostratigraphic framework and/or astronomical age model, but a good magnetostratigraphy exists. The magnetostratigraphy was established in 1977 (*112*) and tested comparing it to the nearby Contessa Highway section (*113*) with new data. The Chauris et al. (*113*) study suggests that a fault at 328 m (Depth as in Alvarez et al. (*112*)) (363 m Coccioni and Premoli Silva (*115*) depth) in the Bottaccione section, located in a normal polarity interval, cut out about 1 million year of strata. This finding is overlooked in subsequent studies and not further investigated thus far. But the observation of Chauris et al. (*113*) is important with respect to the effects on the sedimentation rates and the age model for the Os isotope data located in the C30n normal Chron, where the first Os isotope step is located in 1209 and 1262.

To investigate a different interpretation we first applied the frequently used interpretation for the Bottaccione section following Lowrie and Alvarez (*112*) as given below. The depth estimate for C30n(y) is taken from Montanari and Koeberl (*114*) by calculating the mean between the first normal indicating C30n at 378.35 and the first reversed indicating C29r at 378.6 m. We provide (Table PMag2) ages being consistent with both Cyclo- and AstroAge developed for the ODP records in our study for the Bottaccione section.

Table PMag2: Bottaccione magnetostratigraphic interpretation after (*112*)

Reversal	Depth (m)	Depth Source	CycloAge	AstroAge	Age Source
C29n(o)	384.00	Lowrie and Alvarez (*112*)	65.653	65.653	Westerhold et al. (*2*)
K/Pg	382.60	Coccioni and Premoli Silva (*115*)	66.022	66.022	Dinares-Turell et al. (*76*)
C30n(y)	378.47	Montanari and Koeberl (*114*)	66.346	66.328	this study
C30n(o)	370.80	Coccioni and Premoli Silva (*115*)	68.178	68.178	GPTS2020 (*116*)
C31n(y)	370.60	Coccioni and Premoli Silva (*115*)	68.351	68.351	GPTS2020 (*116*)
C31n(o)	350.00	Coccioni and Premoli Silva (*115*)	69.271	69.271	GPTS2020 (*116*)

The sedimentation rate estimate at Bottaccione from the K/Pg boundary to the Chron C29r/C30n boundary of ~1.3 cm/kyr (Figure Bottaccione 1) is very robust as this interpretation is not in question. Using the Lowrie and Alvarez interpretation the sedimentation rate drops after the C29r/C30n boundary to 0.4 cm/kyr, comparable to the rate after the K/Pg boundary. Globally the sedimentation rates across the K/Pg boundary drop (e.g. (*51*)) due to the breakdown of primary productivity in the surface ocean. Sedimentation rate estimates at Bottaccione jump at the interpreted transition to the C31n Chron from 0.4 to 2.2 cm/kyr. The entire pattern is, compared to other deep sea records (*24*), rather unusual and, taking the observation of Chauris et al. (*113*) into account, needs to be revised.

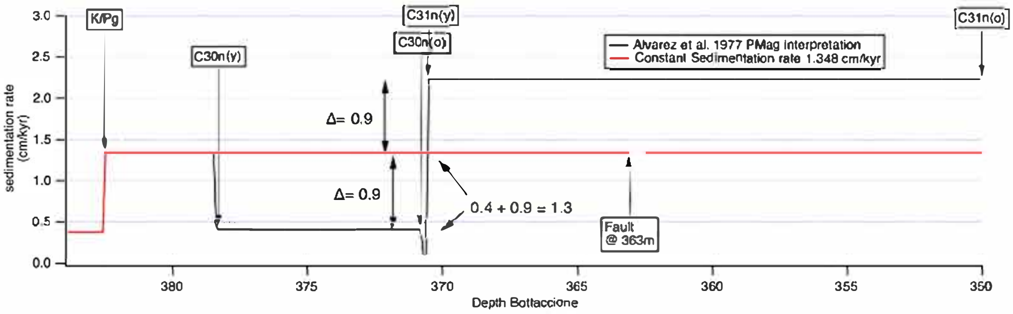

Figure Bottaccione 1: Sedimentation rates for the Bottaccione section using the magnetostratigraphic interpretation from (*112*, black line) and assuming constant sedimentation rates (see text).

The Lowrie and Alvarez (*112*) interpretation of the C30n(o) and C30n(y) is based on one data point around 370.70 m, the described fault is at 363 m (both depth as in Coccioni and Premoli Silva (*115*)) in a normal polarity interval. The C30r Chron, bracketed by C30n(o) and C30n(y), is as can be seen in the record from ODP Site 1262 (Figure S06) relatively short, in the order of 5-6 precession cycles or 100-120 kyr. According to the GPTS2020 this interval spans 173 kyr (*116*) and based on cyclostratigraphic estimates ~120 kyr (*24*). Based on the biostratigraphy of ODP Site 1267 (see Figure S06) the calcareous datum Base *L. quadratus* is close to the bottom of Chron C30n, in line with the finding at ODP Site 762 (*117, 118*). Close to the top of Chron C30n the calcareous datum Base *M. murus* is located in ODP 1267 (Figure S06) and other records like Zumaia. However, this marker is reported to be diachronous with respect to the latitudinal setting of the location (*119*). In the Contessa Highway section and the Bottaccione section (*120*) the Base *M. murus* is placed in the lower part of what is interpreted C30n, based on the Lowrie and Alvarez (*112*) interpretation of C30n(o) and C30n(y). The Base *L. quadratus* is reported in the lower part of what is labeled C31n. The fault found in the Bottaccione section by Chauris et al. (*113*) could also be present unnoticed in the Contessa Highway section as well.

To be independent from the ambiguous interpretation of the magnetostratigraphy towards and below the fault in the Bottaccione section, we assumed constant sedimentation rates between C30n(y) and the fault to get an alternate age model for the Os isotope data (Figure Bottaccione 1, Table PMag3).

Table PMag3 Bottaccione revised magnetostratigraphic interpretation from this study

Reversal	Depth (m)	Depth Source	CycloAge	AstroAge	Age Source
C29n(o)	384.00	Lowrie and Alvarez (*112*)	65.653	65.653	Westerhold et al. (*2*)
K/Pg	382.60	Coccioni and Premoli Silva (*115*)	66.022	66.022	Dinares-Turell et al. (*76*)
C30n(y)	378.47	Montanari and Koeberl (*114*)	66.346	66.328	this study
Fault	363.00	Chauris et al. (*113*)	67.562	67.476	this study*

*assuming constant sedimentation rate of 1.273 (Cyclo) and 1.348 (Astro) cm/kyr between C30n(y) and the fault

In Figure Bottaccione 2 the Robinson et al. (*21*) Os isotope data are plotted on the CycloAge related age model and the Lowrie and Alvarez (*112*) magnetostratigraphic interpretation. As expected the data lower than the C30n(y) boundary are stretched as more time is put into this segment by the magnetostratigraphic interpretation. The constant sedimentation rate model is given by the red dots and we added a unpublished cyclostratigraphic age model of Sinnesael et al. (*104*) of the Bottaccione section for comparison. The constant sedimentation rate model and the unpublished cyclostratigraphic age model show very similar results, suggesting missing time removed by the fault. For the main figure in the study, to compare the Os isoptope data of the Bottaccione section we thus use the constant sedimentation rate model.

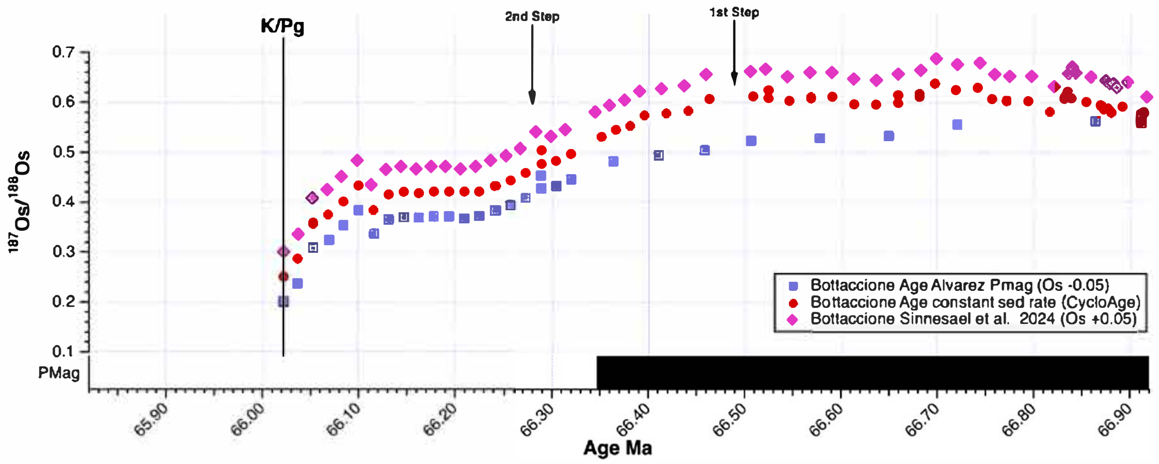

Figure Bottaccione 2: Os isotope data of Robinson et al. (*21*) plotted on the CycloAge related age model and the Lowrie and Alvarez (*112*) magnetostratigraphic interpretation. Osmium data have been offset vertically as given for comparison.

Calcium isotope records of the latest Maastrichtian

The massive perturbations in the Carbon cycle at the K/Pg transition, impact the global Ca system, as the global carbon and Ca cycles are coupled via processes such as continental weathering and carbonate precipitation and dissolution. Mass dependent isotope fractionation processes taking place during some of these processes can induce fluctuations of Ca isotopes in the marine biogenic carbonate records (e.g. (*121*)) and it has been suggested that the Ca isotope record may provide additional insights into the coupled global $CaCO_3$ cycle (e.g. (*122*)). The interpretation of these proxy records in terms is however not straight forward, as the $\delta^{44/40}Ca$ or $\delta^{44/42}Ca$ ($[^{44}Ca/^{40}Ca_{sample} / {}^{44}Ca/^{40}Ca_{standard} - 1] * 1000$; $[^{44}Ca/^{42}Ca_{sample} / {}^{44}Ca/^{42}Ca_{standard} - 1] * 1000$) of marine carbonate records is affected by numerous processes which cannot readily be resolved (e.g. (*123*)). This includes factors affecting the primary Ca isotope fraction during biomineral formation, the composition of the surrounding water and processes related to post depositional alteration. Ca isotope records of mollusks (*124*) and foraminifers (*125*) across the K/Pg boundary show fluctuations in the order of halve a permille that are in general consistent with perturbations in the marine Ca-C system. Both publications propose that the observed Ca isotope variability is mainly caused by changes in the isotope fractionation during biomineral formation, related to the carbonate saturation and pH of the ocean water. Based on the timing of the Ca isotope signal, Deccan Trap volcanism was suggested as the major cause for the changes in the oceanic carbonate system (*124, 125*).

The interpretation of the Ca isotope record is mainly based on the reasoning that 1) the observed Ca isotope changes cannot be explained by shifts in ocean Ca isotopic composition which take place on longer time scales and 2) the Ca isotope fractionation during biogenic and inorganic $CaCO_3$ formation depends on environmental factors such as temperature, carbonate system parameters and precipitation rate (*126, 127*). Additionally, changes in $\delta^{44/40}Ca$ of local (restricted) water masses (*128*) and changes in porewater Ca isotope composition due to carbonate dissolution, may have influenced the Ca isotope records. This may in particular be relevant for the applied endo-benthic species and times of low carbonate saturation. In addition the relation between calcite saturation/pH and Ca fractionation factor is not sufficiently validated for the applied archives, to clearly distinguish different processes. In particular for planktic and benthic foraminifers (*129*) it has been reported that interfering environmental parameters cause complicated species specific fractionation patterns that restricts a detailed paleo-environmental reconstruction. Nevertheless the isotope records of Linzmeyer et al. (*124*) and Jouini et al. (*125*) are fully consistent with perturbations of the C cycle in response to the events at the K/Pg transition.

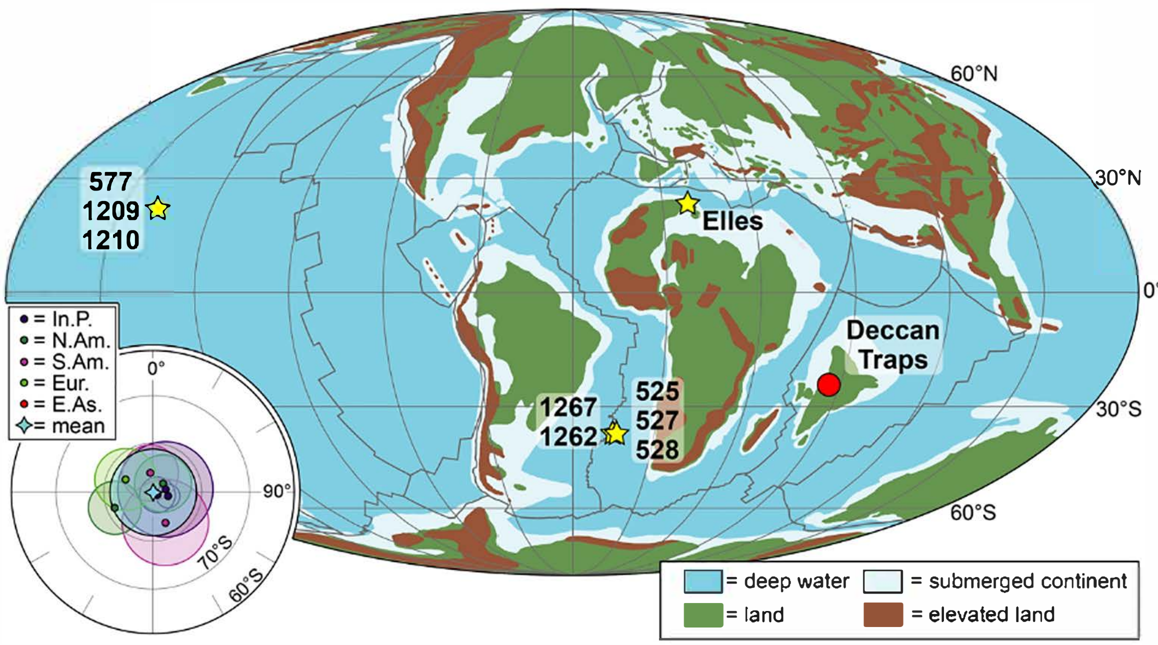

Fig. S1 Paleogeographic map 66 Ma.

The position of the continent, ocean basins, plate boundaries, and the deep-sea sites used for this work is determined by using the global relative plate motion model of Müller et al. (M16) (*130*). The main paleogeographic features (exposed and submerged continent, elevated land) are after Cao et al. (*131*). The global reconstruction is anchored to the Earth's spin axis by using seven selected paleomagnetic poles (66±5 Ma) from North America (N.Am.) (*132, 133*), South America (S.Am.) (*134*), stable Europe (Eur.) (*135*), and India-Pakistan (In.P.) (*136-138*). The single poles are rotated using the M16 model and then averaged by standard spherical statistic (*139*). All plates have been consequently rotated by forcing the average pole to coincide with the geographic south (bottom left inset of figure). Map drawn with GPlates (*140*). Map of records used in this study.

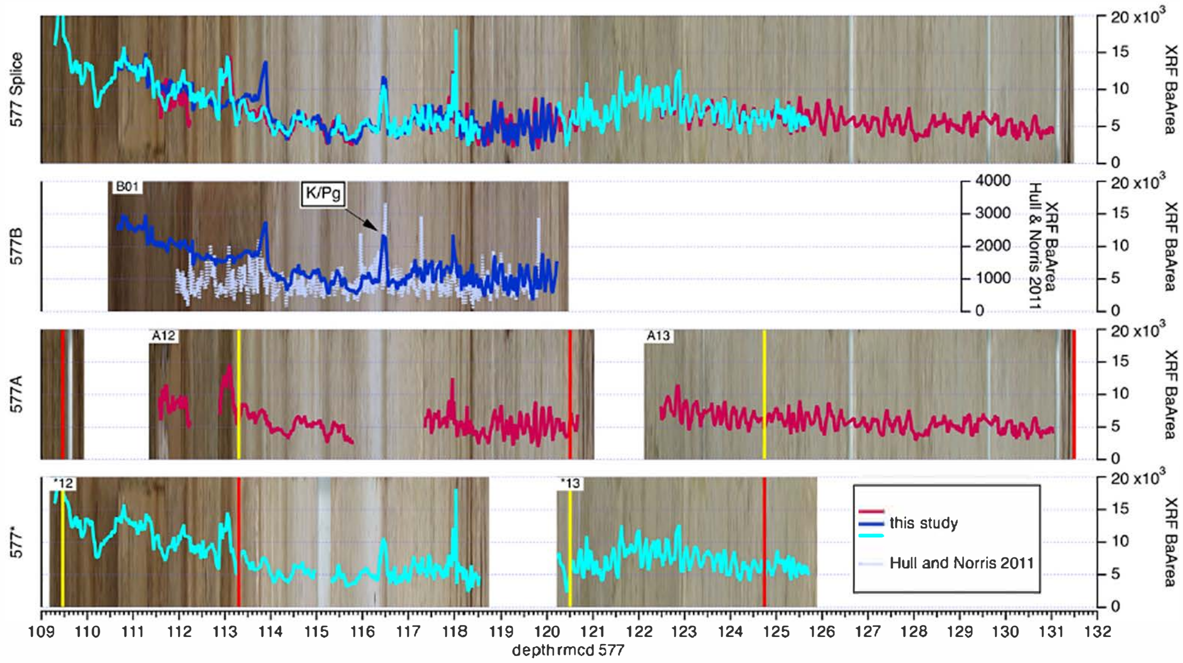

Fig. S2 DSDP577 X-ray fluorescence barium data.

Published and newly generated X-ray fluorescence core scanning barium elemental intensity data for DSDP Site 577. Here for Hole 577*, Hole 577A, Hole 577B and the new composite record for Site 577 underlain by core images. Yellow and red bars mark the top and bottom of the splice intervals in the respective core.

a,

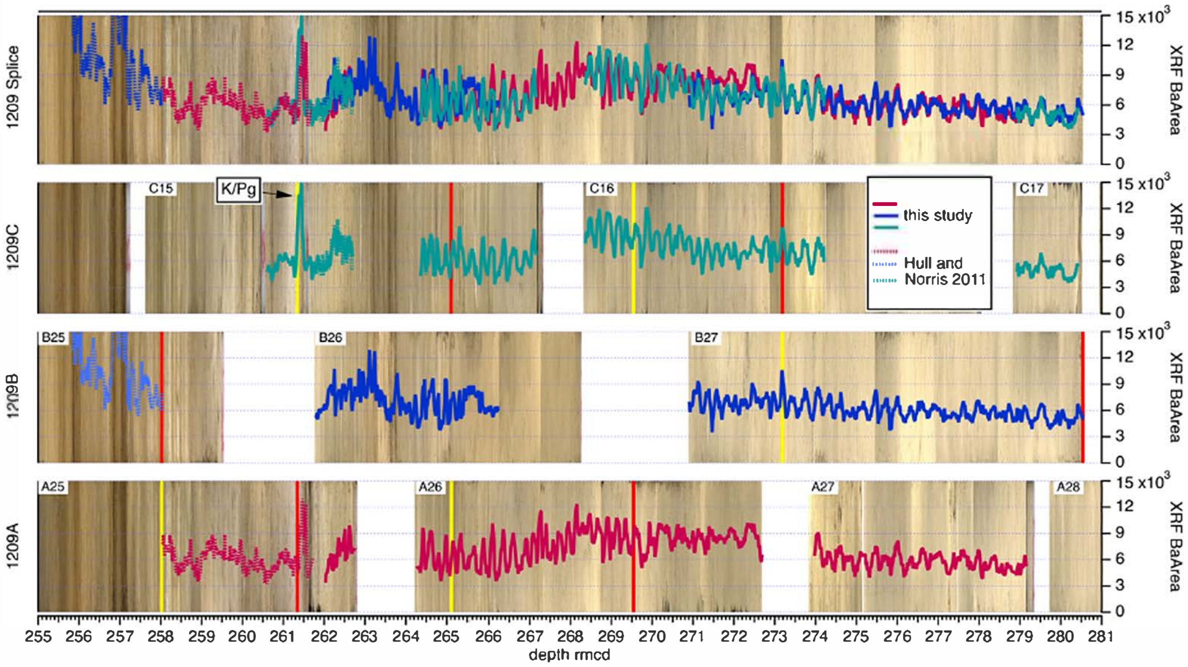

b,

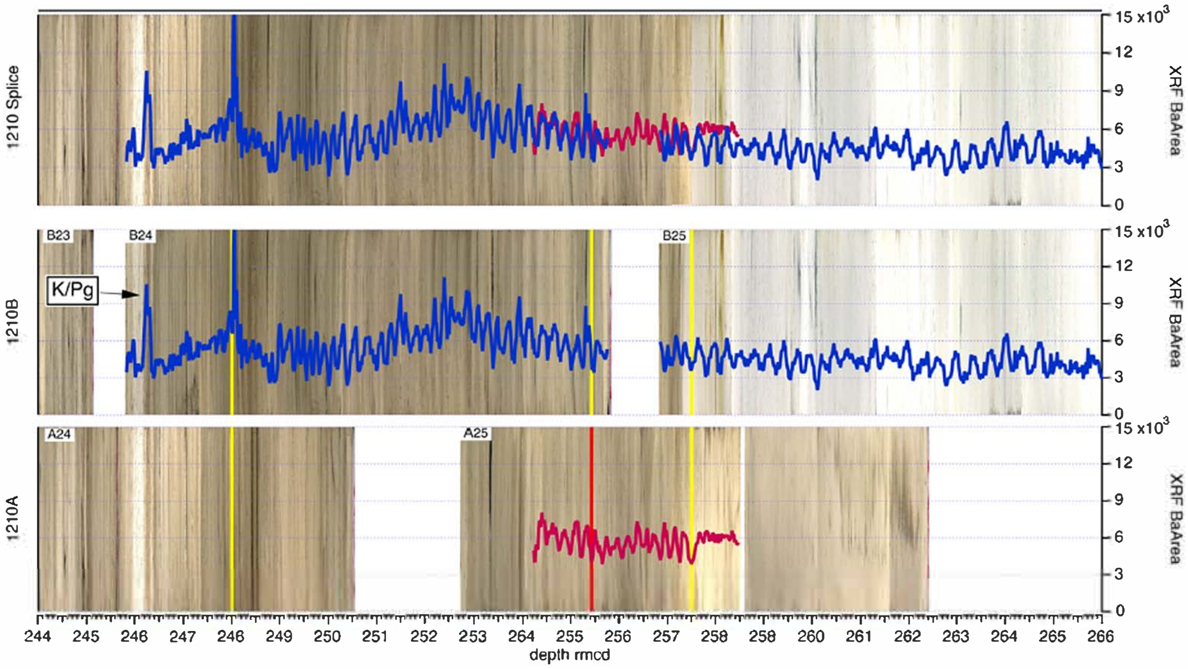

Fig. S3 ODP Site 1209 and 1210 X-ray fluorescence barium data.
Published X-ray fluorescence core scanning barium elemental intensity data for ODP Site 1209 (a) and Site 12010 (b). Here for Hole 1209A, Hole 1209B, Hole 1209C in (a) as well as Hole 12010A and 1210B in (b) including the new composite record.

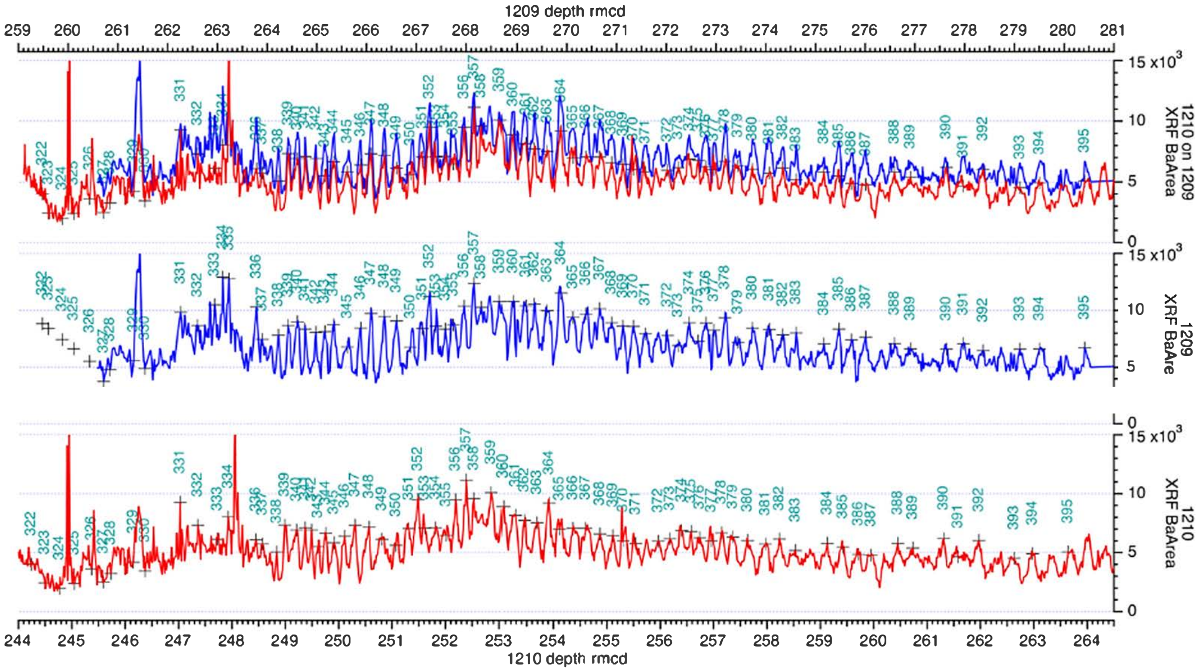

Fig. S4 Correlation between Shatsky Rise ODP Site 1209 and Site 1210.

This figure shows the very detailed correlation and integration based on the cyclic variations present in the X-ray fluorescence core scanning barium elemental intensity data of ODP Site 1209 and Site 12010 to form a composite sequence. The data and correlation are from Kim et al. 2020 (*45*).

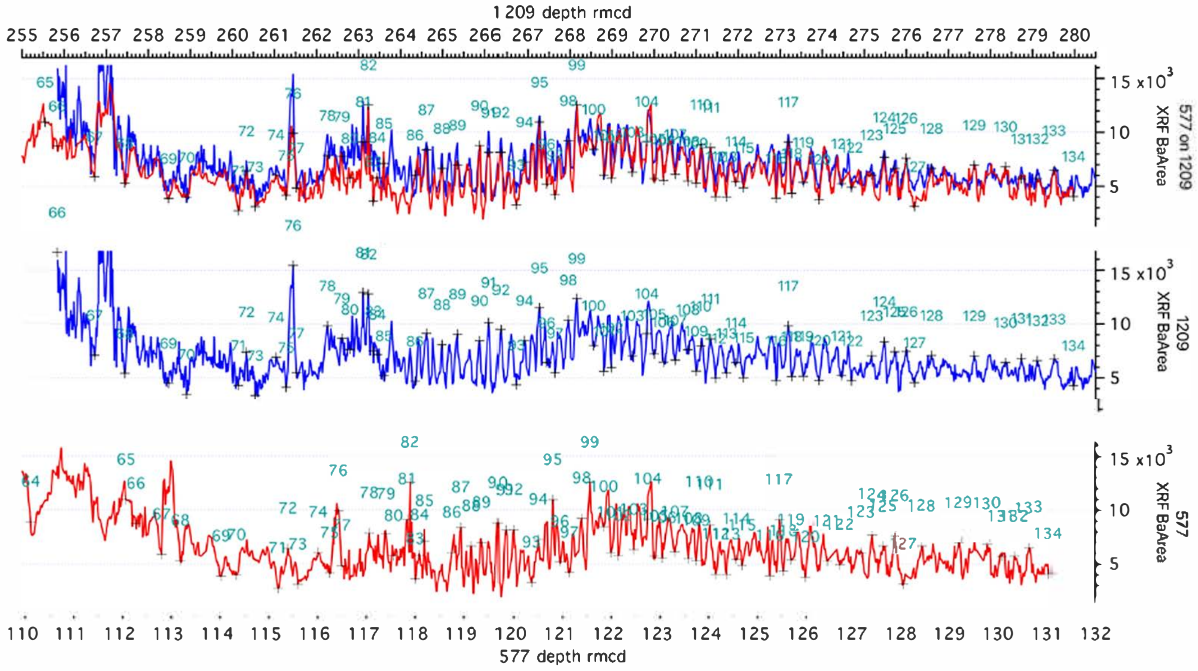

Fig. S5 Correlation between Shatsky Rise DSDP Site 577 and ODP Site 1209.

This figure is to show the very detailed correlation and integration based on the cyclic variations present in the X-ray fluorescence core scanning barium elemental intensity data of DSDP Site 577 with ODP Site 1209.

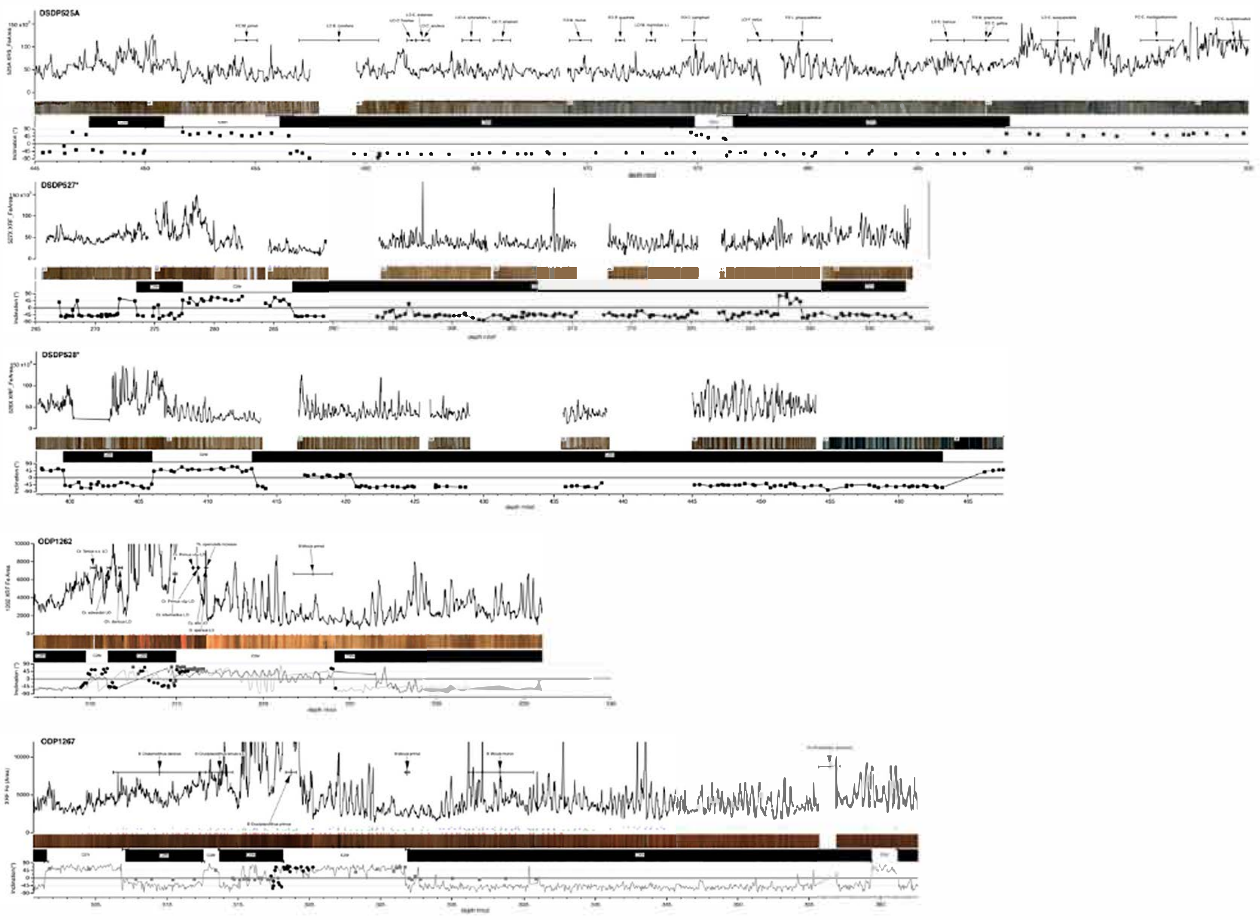

Fig. S6 Oversized – Overview of DSDP and ODP records from Walvis Ridge.

X-ray fluorescence core scanning iron elemental intensity data, bio- and magnetostratigraphy, core images and natural remanent magnetization inclination data for 525A, 527*, 528*, 1262, and 1267. Color coded numbers mark correlation tie points (Data S7) mapping all sites to the based Site 1267 depth.

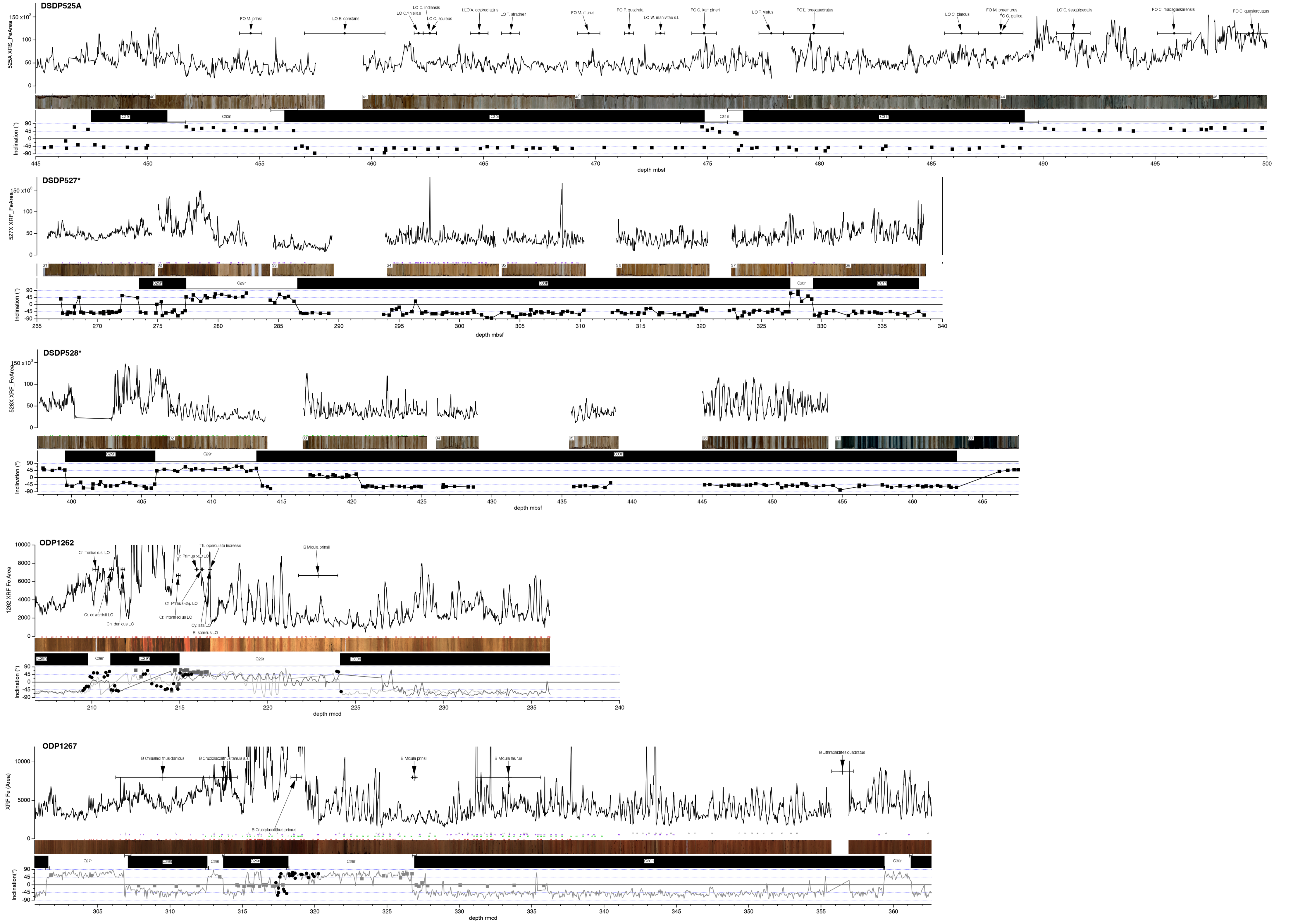
DSDP525A
525A XRS_FeArea
Inclination (°)
depth mbsf
FO M. prinsii
LO B. constans
LO C. indiensis
LO C. aculeus
LLO A. octoradiata s
LO T. stradneri
FO M. murus
FO P. quadrata
LO W. manivitae s.l.
FO C. kamptneri
LO P. vetus
FO L. praequadratus
LO C. biarcus
FO M. praemurus
FO C. gallica
LO C. sesquipedalis
FO C. madagaskarensis
FO C. quasiarcuatus
C30n
C31n
DSDP527*
527X XRF_FeArea
C29r
C30r
DSDP528*
528X XRF_FeArea
C29r
ODP1262
1262 XRF Fe Area
depth rmcd
Cr. Tenius s.s. LO
Cr. edwardsii LO
Ch. danicus LO
Cr. Primus >5μ LO
Cr. Primus <5μ LO
Cr. intermedius LO
Th. operculata increase
Cy. alta LO
B. sparsus LO
B Micula prinsii
C28r
C29r
ODP1267
XRF Fe (Area)
depth rmcd
B Chiasmolithus danicus
B Cruciplacolithus tenuis s.s.
B Cruciplacolithus primus
B Micula prinsii
B Micula murus
B Lithraphidites quadratus
C27r
C28r
C29r
C30r

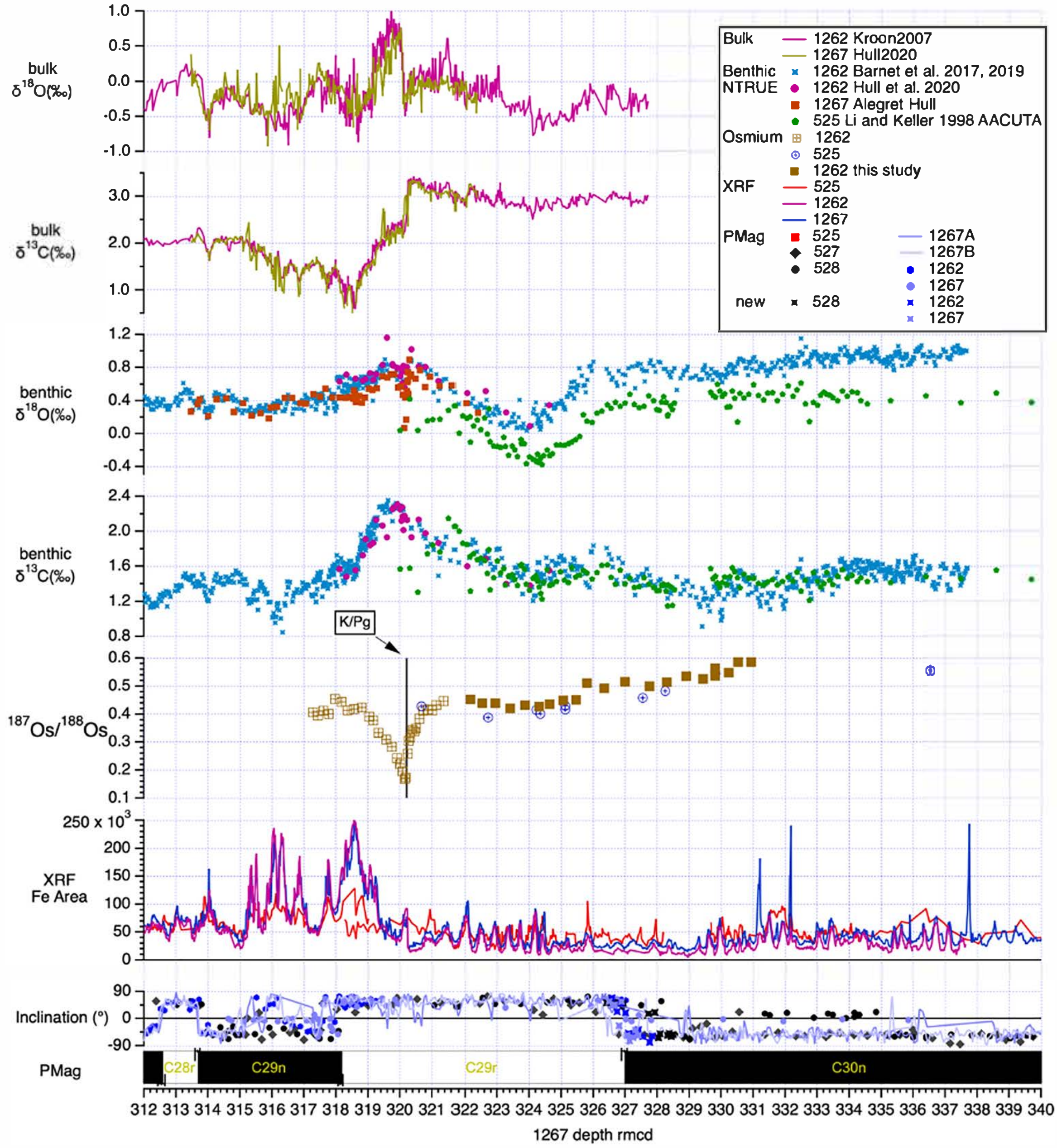

Fig. S7 Full data set for South Atlantic Walvis Ridge on ODP Site 1267 depth.

All published and newly generated magnetostratigraphic, X-Ray fluorescence iron elemental intensity, osmium isotope, benthic and bulk stable carbon and oxygen isotope data from DSDP Sites 525, 527, 528 and ODP Sites 1262, 1267 integrated onto the depth of base Site 1267.

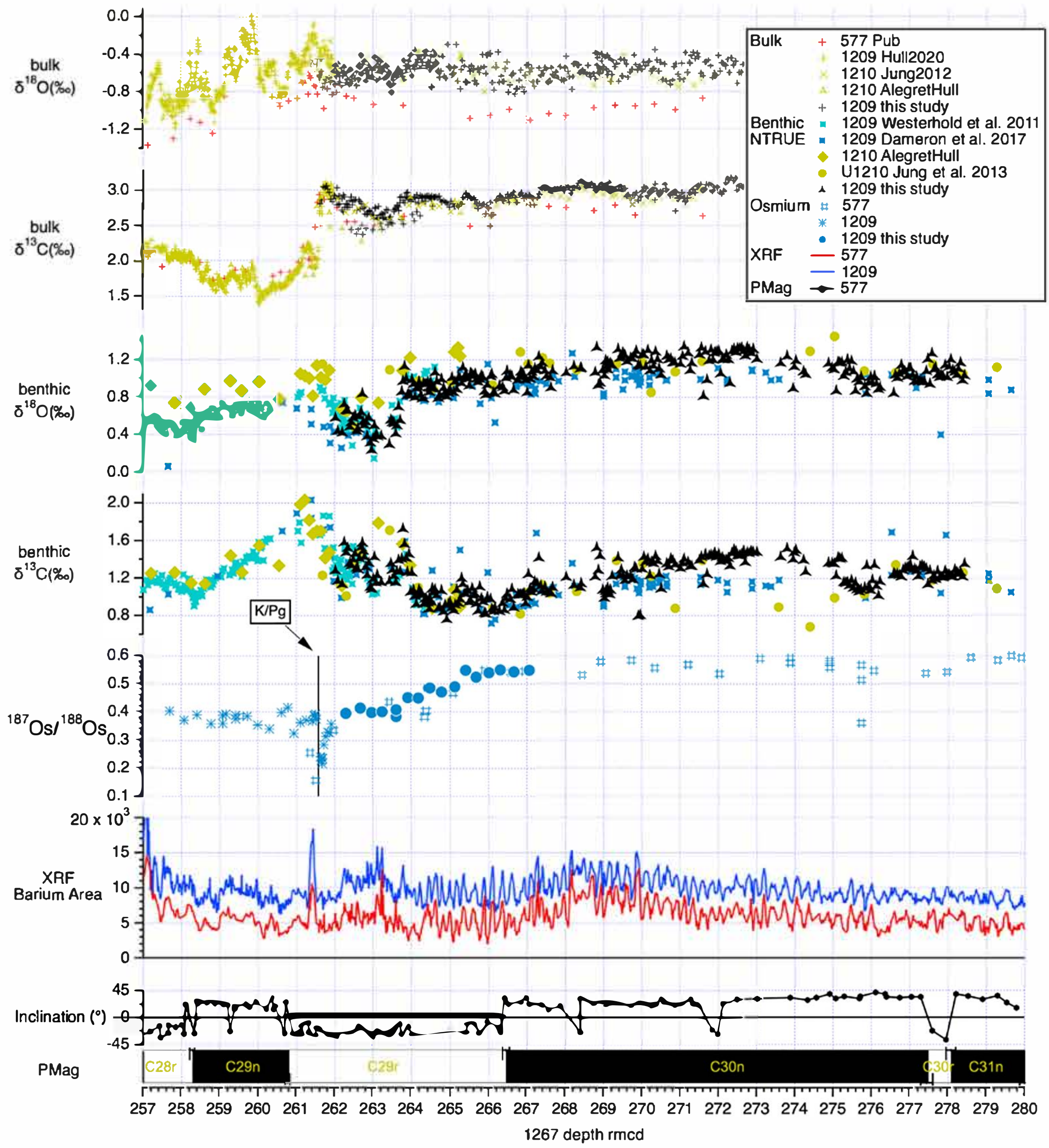

Fig. S8 Full data set for equatorial Pacific Shatsky Rise on ODP Site 1267 depth. All published and newly generated magnetostratigraphic, X-Ray fluorescence iron elemental intensity, osmium isotope, benthic and bulk stable carbon and oxygen isotope data from DSDP Sites 577 and ODP Sites 1209, 1210 integrated onto the depth of base Site 1267.

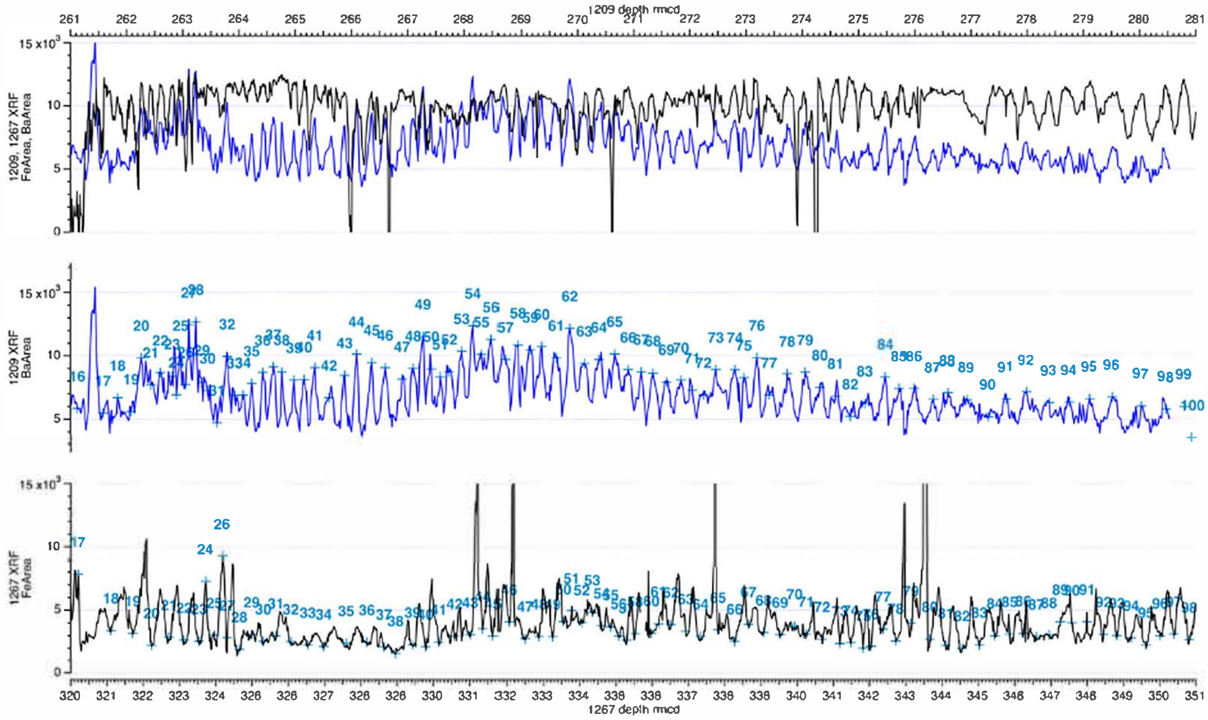

Fig. S9 Correlation between Shatsky Rise Site 1209 and Walvis Ridge Site 1267.

X-Ray fluorescence barium (Site 1209) and iron (Site 1262) elemental intensity data with mapping pairs indicated by numbers (Data S7). Correlation was done tying minima in 1262 Fe XRF to maxima in 1209 Ba XRF following the cycle identification and counting as shown in the next figure. Note that in the top graph 1262 Fe XRF data have been inverted to better illustrate the match.

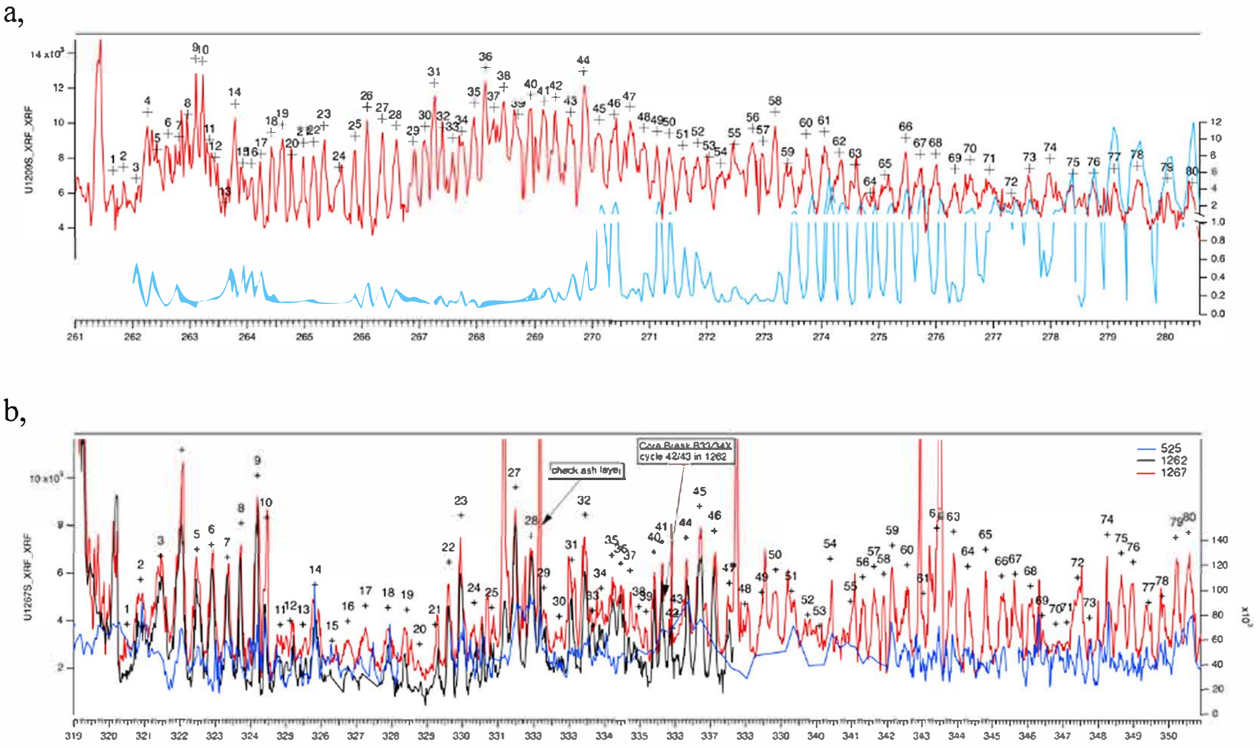

Fig. S10 Precession cycle counting of key records from Shatsky Rise and Walvis Ridge. Precession cycle counting of (a) ODP Site 1209 X-Ray fluorescence barium (red line) and coarse fraction weight % data (blue line, note the splice axis to account for the large range in data); (b) DSDP Site 525, ODP Sites 1262 and 1267 X-Ray fluorescence Iron data.

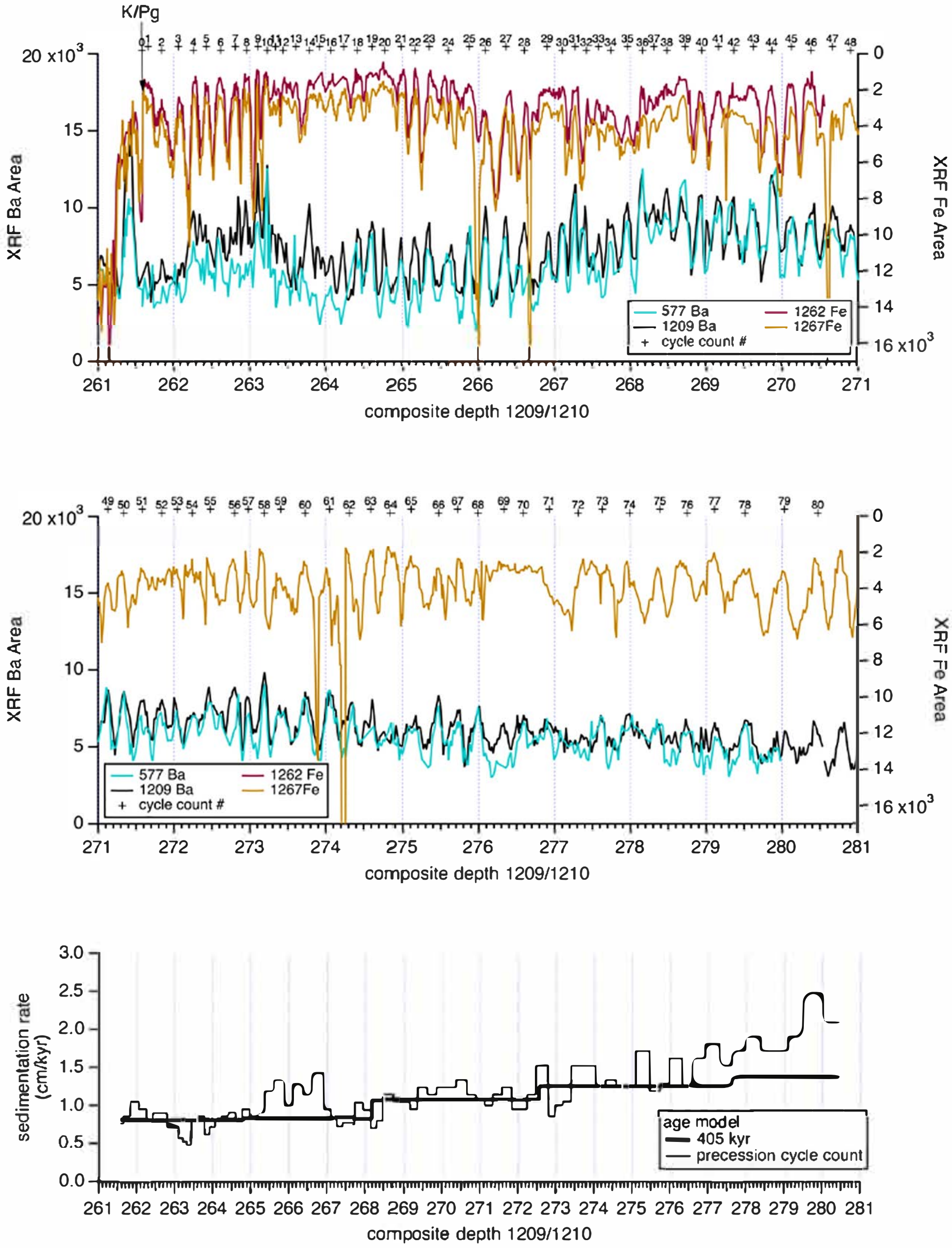

Fig. S11 Comparison of Astrochronology and Cyclostratigraphy.

Top two graphs show the precession cycle counting for the cyclostratigraphic age model for the Shatsky Rise records of 577 and 1209 and the Walvis ridge record of 1262 and 1267. Bottom graph compares the sedimentation rate resulting from precession cycle based cyclostratigraphy and the astronomically calibrated age model using the stable 405-kyr eccentricity cycle (*45*). Both age models yield similar sedimentation rates with the cyclostratigraphy showing more variability, in particular the 405-kyr eccentricity minima are related to higher sedimentation rates in the cyclostratigraphy model as cycle tend to be thicker in this interval (e.g. 266 meters composite depth).

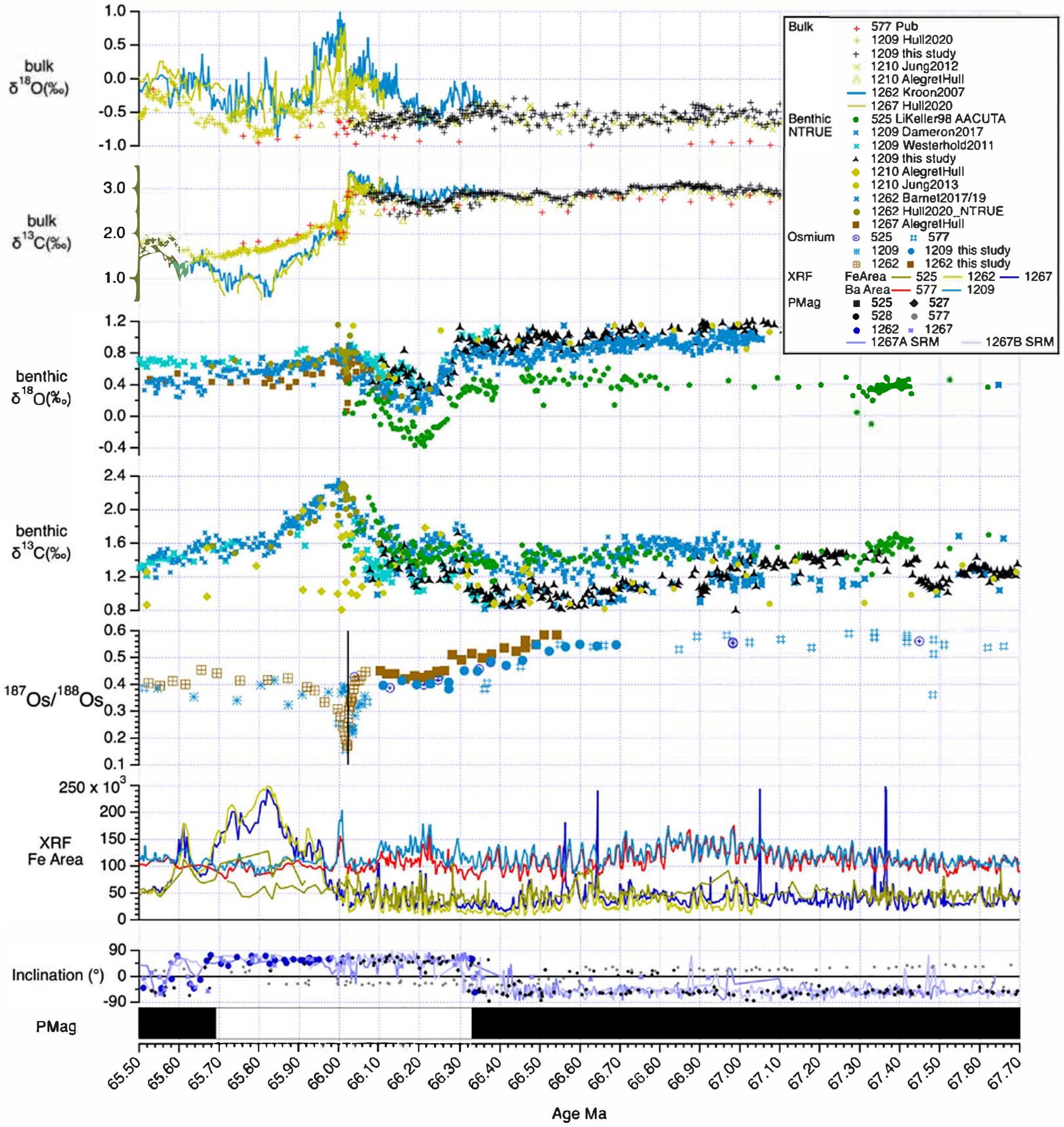

Fig. S12 Full data set for Shatsky Rise and Walvis Ridge on astrochronology. All published and newly generated magnetostratigraphic, X-Ray fluorescence iron elemental intensity, osmium isotope, benthic and bulk stable carbon and oxygen isotope data from Shatsky Rise and Walvis Ridge DSDP and ODP Sites on the astronomically calibrated age model.

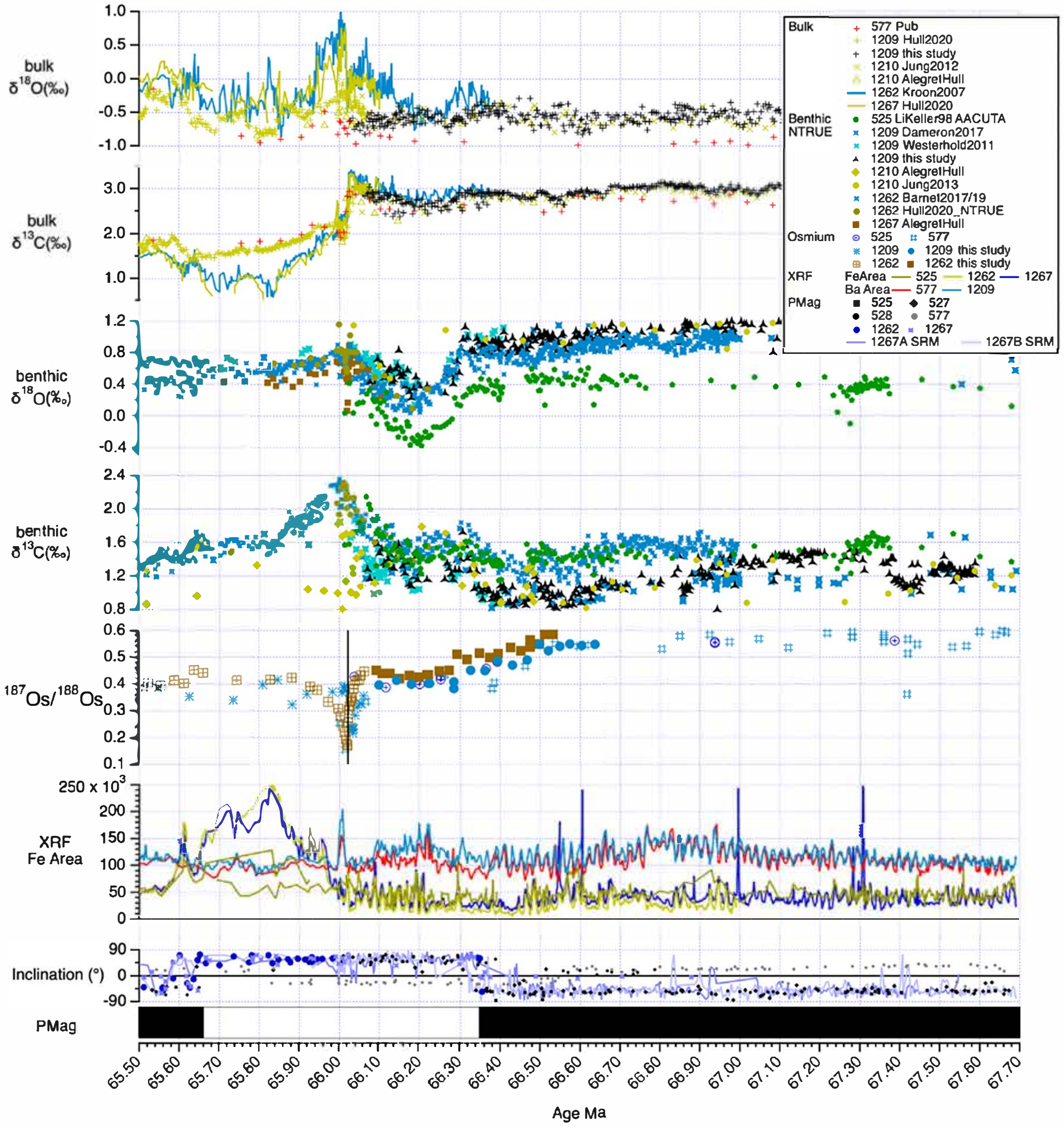

Fig. S13 Full data set for Shatsky Rise and Walvis Ridge on cyclostratigraphy.
All published and newly generated magnetostratigraphic, X-Ray fluorescence iron elemental intensity, osmium isotope, benthic and bulk stable carbon and oxygen isotope data from Shatsky Rise and Walvis Ridge DSDP and ODP Sites on the precession cycle counting cyclostratigraphy age model.

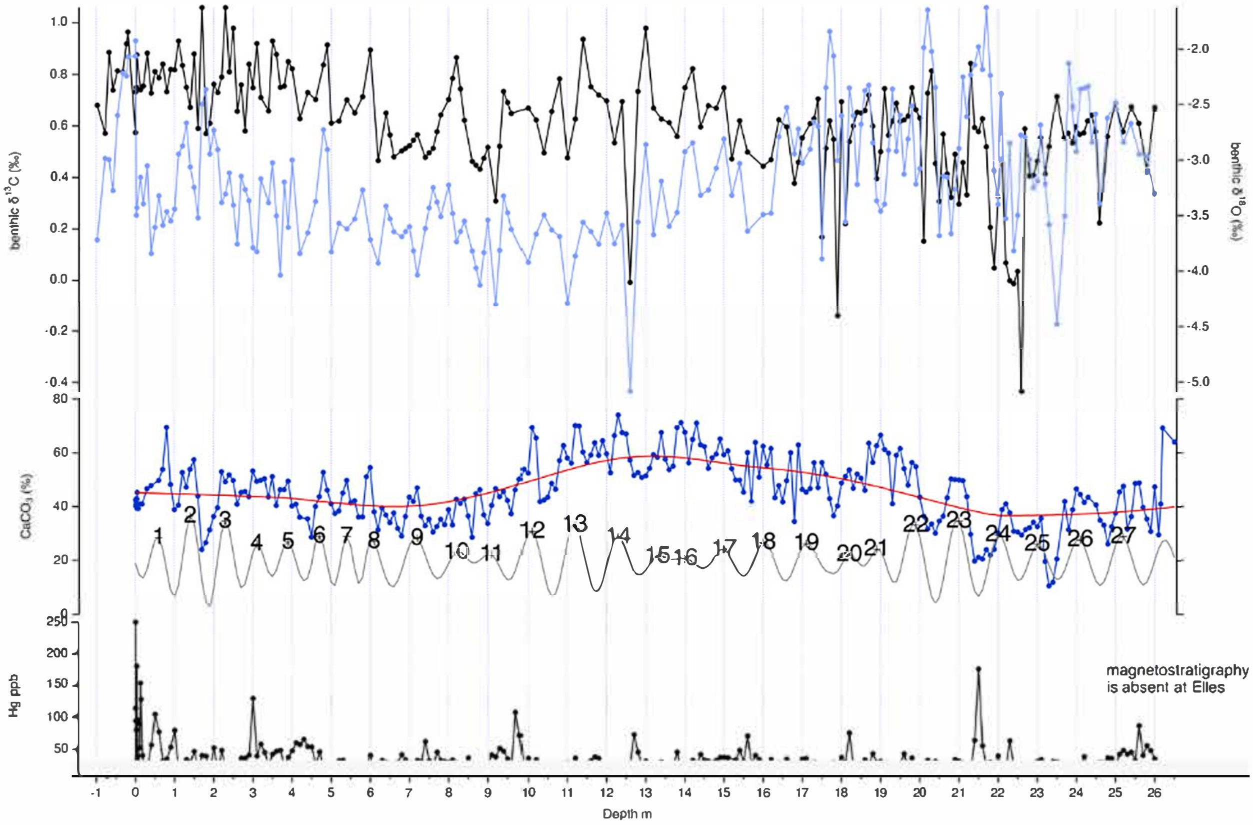

Fig. S14 Tunesian Elles section cyclostratigraphic interpretation.

Mercury concentrations, calcium carbonate content and benthic foraminifer stable carbon and oxygen isotope data from the Elles section in Tunesia (*12, 92, 93*). The gray line shows the filtered precession cycle (*12*) component in the carbonate content data used to establish a cyclostratigraphy consistent with the precession cycle counting age model for the Shatsky Rise and Walvis Ridge records. Numbers are equivalent to precession cycle numbers for Shatsky Rise and Walvis Ridge, thus can be seen as correlation tie points as well (Table S2).

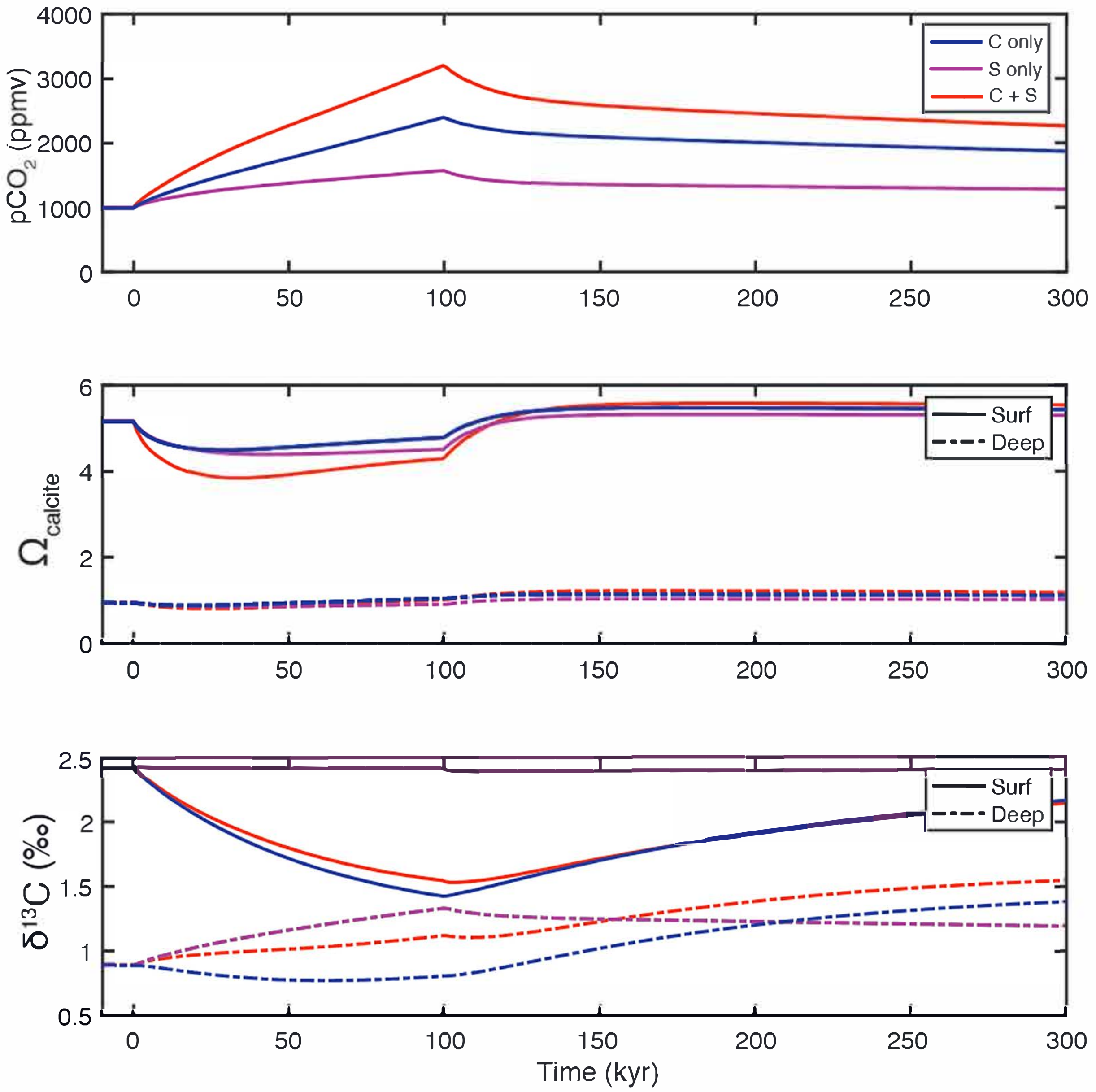

Fig. S15 LOSCAR idealized C and S emissions experiments.

LOSCAR v.2.0.4 simulations of carbon and sulfur volatile release. SO_2 release (which would rapidly oxidize to sulfuric acid and rain out into the ocean) is represented by a reduction in surface seawater total alkalinity. The "C only" run features 10,000 Gt C, the "S only" features 10,000 Gt S, and the "C+S" includes both. All emissions are released at a constant rate over 100kyr (model year 0 to 100,000 in the above plots). Plotted are atmospheric pCO_2, calcite saturation state of the surface and deep ocean (averaged across all basins), and carbon isotopic composition ($\delta^{13}C$ in ‰ relative to V-PDB) of dissolved organic carbon in the surface and deep ocean. Each experiment is plotted in a different color, while surface tracers are plotted in solid lines while deep tracers are dashed. Results are discussed in the main text and in the supplementary text.

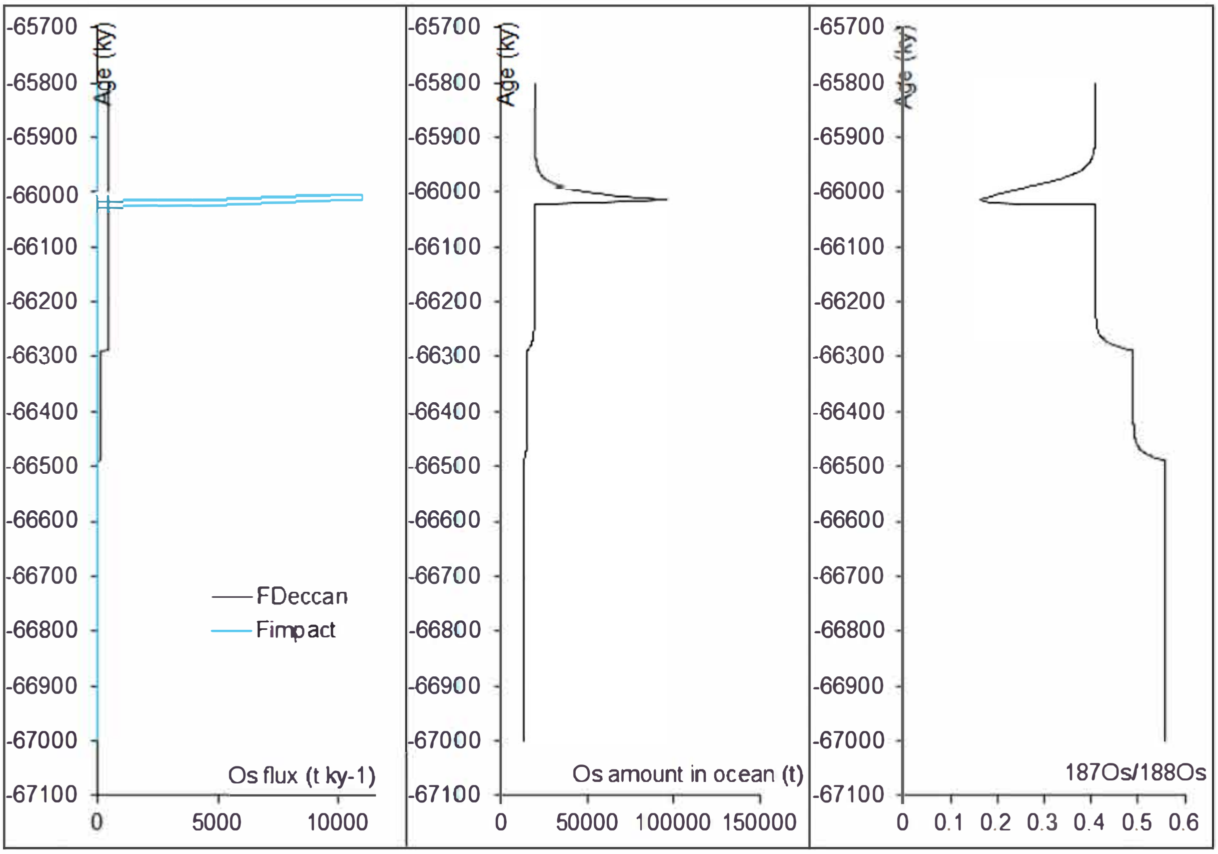

Fig. S16 Os Box Model results

Left: Os flux from the DT basalt (black lines) and K-Pg impact (light blue), Middle: Os amount in ocean, Right: $^{187}Os/^{188}Os$ of seawater.

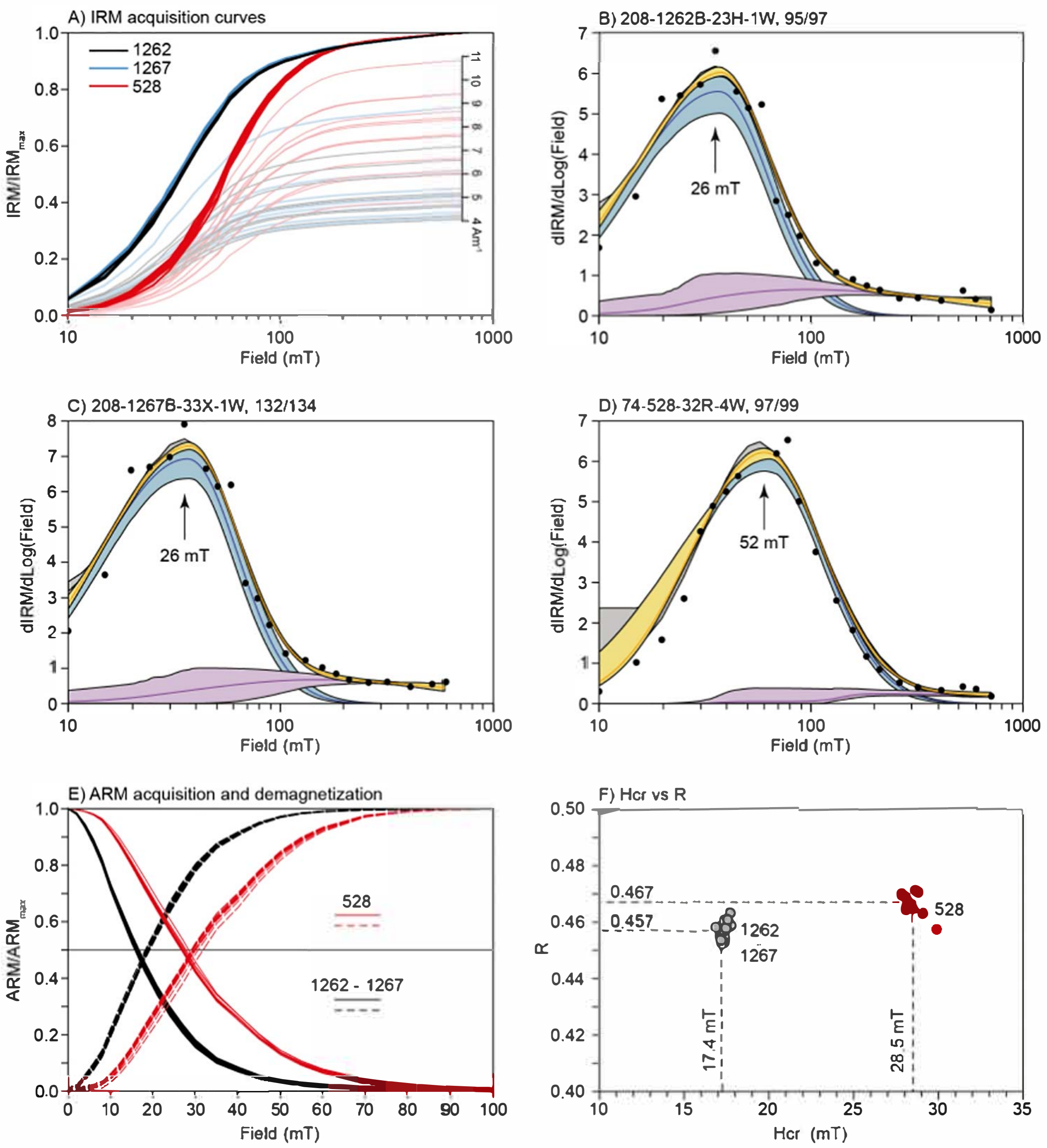

Fig. S17 Rock-magnetic analyses results.

A) Isothermal remanent magnetization acquisition curves of all specimens, plotted on a logarithmic field scale, showed with both absolute intensity (curves in the background) and normalized (curves in the front). B-D) Results of the IRM curves "unmixing" for a representative sample from each site indicated on the panel; the black dots are the first derivative data of the IRM curve, the blue curve is the low coercivity modeled component, with the associated 95% bootstrapped confidence boundary, the purple line is the modeled high coercivity component with the 95% confidence, the yellow curve is the sum of the two components (*85*); the number within each panel is the half saturation field of the low coercivity component. E) Normalized anhysteretic remanent magnetization (ARM) acquisition (dashed lines) and demagnetization (solid line). F) Diagram of the parameter R (*106*) determined as explained in the main text, plotted against the remanence coercivity Hcr estimated by the crossing point between the ARM acquisition and demagnetization curve of each sample.

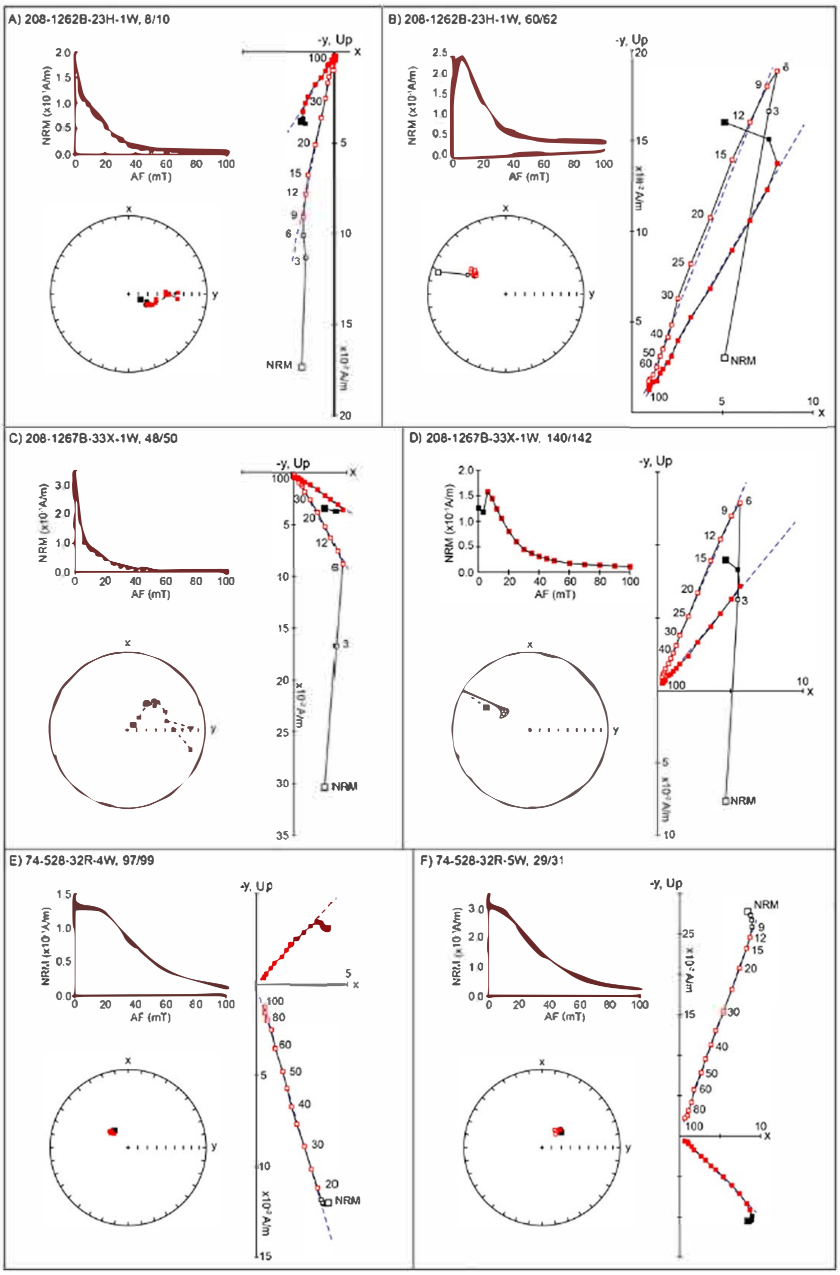

Fig. S18 Representative vector end-point diagrams (*81*) from Site 1262 (A-B), 1267 (C-D), and 528 (E-F), with indication of the sample code.

In each panel are shown: the natural remanent magnetization (NRM) intensity variation during alternate field (AF) demagnetization (top left); the equal area projection of the vector end-points (bottom left; solid and empty symbols are projection onto the lower and upper hemisphere, respectively); the vector end-point diagram (right), with solid and empty symbols indicating the projection of the point onto the horizontal and vertical plane, respectively, and demagnetization steps in mT. In all diagrams, the red symbols are the ones selected for estimating the characteristic remanent magnetization (ChRM) component. The coordinates of the diagrams follow the standard right-hand convention (*80*).

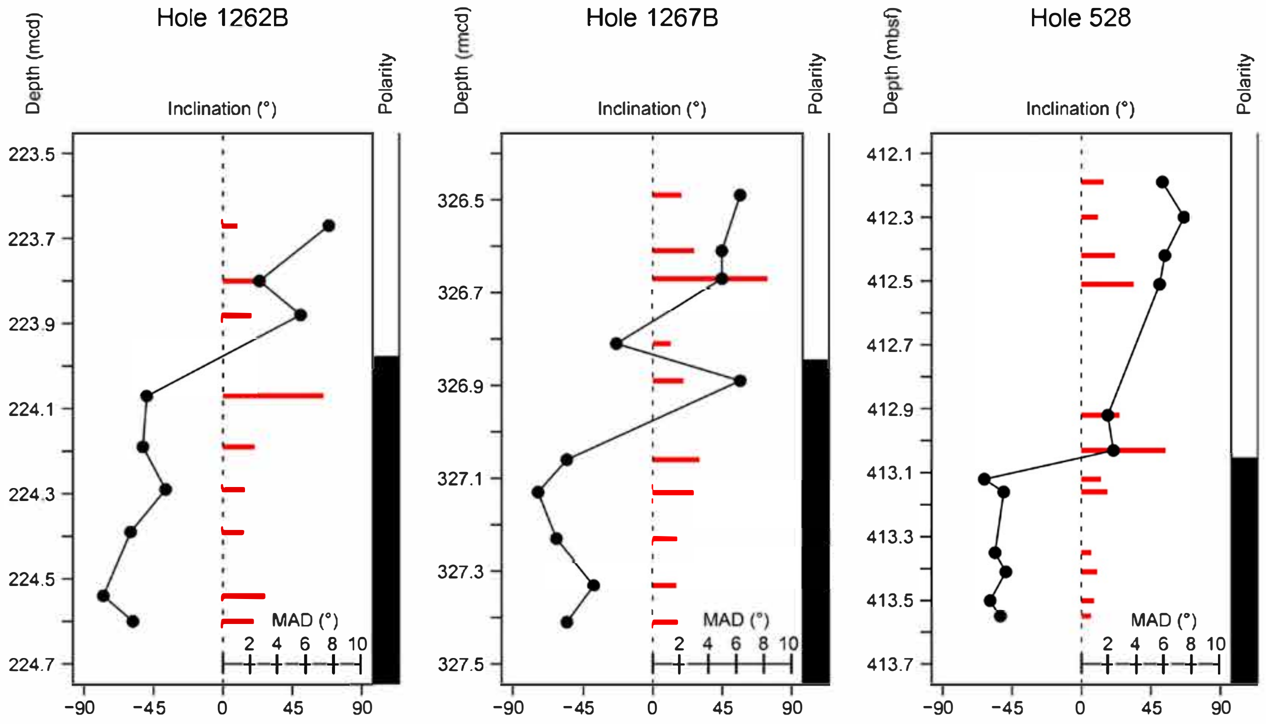

Fig. S19 Magnetic polarity stratigraphy of Hole 1262B, 1267B, and 528.

The magnetic polarity is interpreted by means of paleomagnetic inclination, with positive inclination representing reversed polarity (white band to the right hand side of each column), while negative inclination interpreted as normal (black right band). In each column it is shown also the maximum angular deviation (MAD; (*83*)) associate to each paleomagnetic direction.

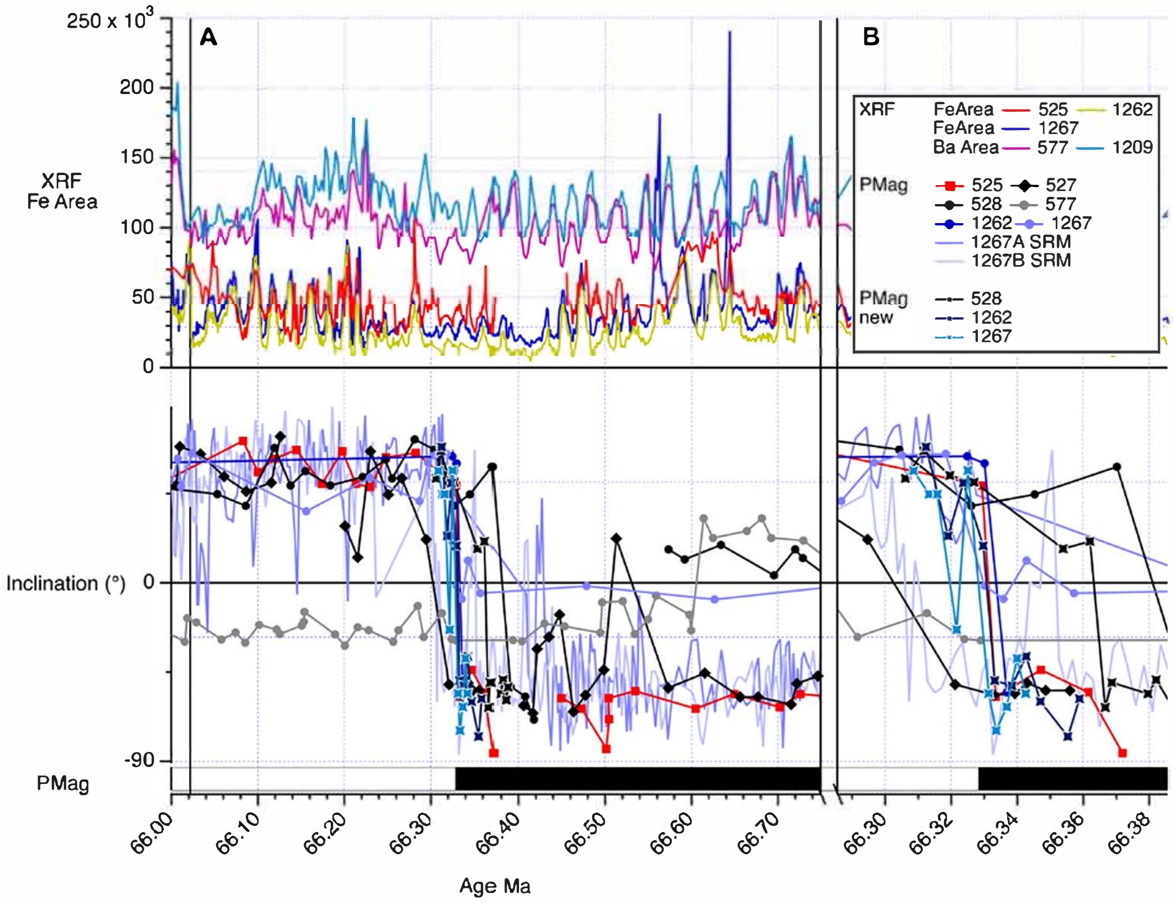

Fig. S20 Magnetostratigraphic data on astrochronology.

Published and newly generated Natural Remanent Magnetism (NRM) inclination data for Shatsky Rise and Walvis Ridge in the bottom graph. Discrete sample data are plotted with markers and lines, shipboard NRM data on section halves (SRM) are also plotted as lines. For reference to the cyclicity the top graph displays the X-Ray fluorescence iron and barium elemental intensity data. Part A of the figure shows all data from 66 to 66.75 Ma; part B shows a zoomed in 100 kyr interval cross the reversal.

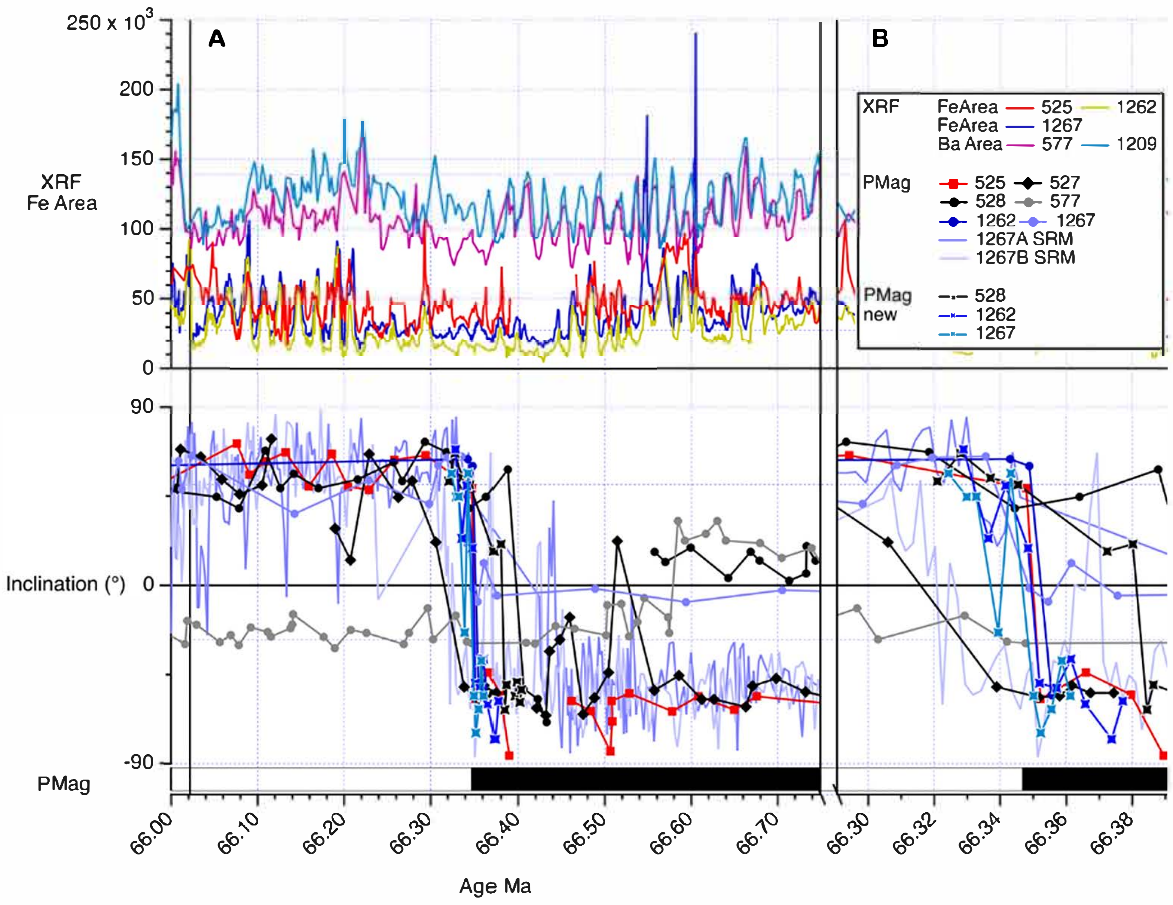

Fig. S21 Magnetostratigraphic data on cyclostratigraphy.

See capture Fig. S20.

Table S1 ODP Sites 1209 basic 405 kyr astronomical age model.

meters composite depth 1209	Age Ma	Tie Note	Source
249.860	62.510	tuned to 1262	(*76*)
251.230	62.917	tuned to 1262	(*76*)
252.520	63.324	tuned to 1262	(*76*)
253.070	63.703	tuned to 1262	(*76*)
253.150	63.731	tuned to 1262	(*76*)
253.370	63.808	tuned to 1262	(*76*)
253.690	63.901	tuned to 1262	(*76*)
254.120	63.990	tuned to 1262	(*76*)
254.510	64.096	tuned to 1262	(*76*)
254.800	64.201	tuned to 1262	(*76*)
255.100	64.308	tuned to 1262	(*76*)
255.470	64.425	tuned to 1262	(*76*)
255.610	64.528	tuned to 1262	(*76*)
255.920	64.635	tuned to 1262	(*76*)
256.400	64.735	tuned to 1262	(*76*)
256.840	64.832	tuned to 1262	(*76*)
257.100	64.932	tuned to 1262	(*76*)
257.540	65.035	tuned to 1262	(*76*)
258.180	65.132	tuned to 1262	(*76*)
258.360	65.225	tuned to 1262	(*76*)
258.800	65.329	tuned to 1262	(*76*)
259.060	65.434	tuned to 1262	(*76*)
259.640	65.527	tuned to 1262	(*76*)
259.940	65.601	tuned to 1262	(*76*)
260.100	65.707	tuned to 1262	(*76*)
260.660	65.812	tuned to 1262	(*76*)
261.120	65.911	tuned to 1262	(*76*)
261.320	65.994	tuned to 1262	(*76*)
261.580	66.022	K/Pg boundary	(*76*)
264.810	66.420	La2004CosineFunction Minimum	(*45*)
268.200	66.826	La2004CosineFunction Minimum	(*45*)
272.560	67.230	La2004CosineFunction Minimum	(*45*)
277.630	67.636	La2004CosineFunction Minimum	(*45*)
283.188	68.040	La2004CosineFunction Minimum	(*45*)
289.474	68.446	La2004CosineFunction Minimum	(*45*)
294.240	68.850	La2004CosineFunction Minimum	(*45*)
299.200	69.256	La2004CosineFunction Minimum	(*45*)
304.4106	69.660	La2004CosineFunction Minimum	(*45*)
309.410	70.066	La2004CosineFunction Minimum	(*45*)
315.570	70.470	La2004CosineFunction Minimum	(*45*)
320.720	70.876	La2004CosineFunction Minimum	(*45*)
326.140	71.280	La2004CosineFunction Minimum	(*45*)

Table S2 Cyclostratigraphic Age Model.

Cycle No. #	Cycle Count Age Ma	Depth Zumaia (*23*)	Depth Elles (*93*)	Composite Depth Site 1209
K/Pg boundary	66.022	0.00	0.00	261.58
1	66.033	0.55	0.60	261.66
2	66.054	1.25	1.40	261.84
3	66.075	2.08	2.30	262.06
4	66.096	3.03	3.10	262.26
5	66.117	4.03	3.90	262.43
6	66.138	5.00	4.70	262.62
7	66.159	6.00	5.40	262.81
8	66.180	7.03	6.10	262.96
9	66.201	7.98	7.20	263.11
10	66.222	8.80	8.20	263.23
11	66.243	9.60	9.10	263.34
12	66.264	10.33	10.10	263.44
13	66.285	10.88	11.20	263.61
14	66.306	11.40	12.30	263.79
15	66.327	12.05	13.30	263.92
16	66.348	12.75	14.00	264.07
17	66.369	13.43	15.00	264.24
18	66.390	14.08	16.00	264.42
19	66.411	14.75	17.10	264.61
20	66.432	15.40	18.20	264.78
21	66.453	15.95	18.90	264.98
22	66.474	16.55	19.90	265.16
23	66.495	17.38	21.00	265.35
24	66.516	18.35	22.00	265.60
25	66.537	19.35	23.00	265.88
26	66.558	20.30	24.10	266.09
27	66.579	21.25	25.20	266.36
28	66.600	22.25	26.20	266.60
29	66.621	23.23		266.90
30	66.642	24.00		267.10
31	66.663	24.75		267.27
32	66.684	25.63		267.42
33	66.705	26.45		267.58
34	66.726	27.28		267.74
35	66.747	28.00		267.96
36	66.768	28.63		268.16
37	66.789	29.30		268.31
38	66.810	30.10		268.48
39	66.831	30.93		268.72
40	66.852	31.65		268.94
41	66.873	32.43		269.16
42	66.894	33.25		269.36
43	66.915	34.08		269.62
44	66.936	34.95		269.86
45	66.957	35.90		270.12
46	66.978	36.88		270.38
47	66.999	37.83		270.66
48	67.020	38.78		270.90
49	67.041	39.65		271.13
50	67.062	40.50		271.34
51	67.083	41.45		271.58
52	67.104	42.43		271.84
53	67.125	43.25		272.04
54	67.146	43.98		272.24
55	67.167	44.73		272.48
56	67.188	45.45		272.80
57	67.209	46.05		272.98
58	67.230	46.55		273.19
59	67.251	47.03		273.41
60	67.272	47.63		273.73
61	67.293	48.35		274.05
62	67.314	49.13		274.31

Cyclostratigraphic Age Model - *continued*

**Cycle No. #**	**Cycle Count Age Ma**	**Depth Zumaia (*23*)**	**Depth Elles (*93*)**	**Composite Depth Site 1209**
63	67.335	49.90		274.59
64	67.356	50.55		274.85
65	67.377	51.23		275.11
66	67.398	51.90		275.47
67	67.419	52.63		275.72
68	67.440	53.50		275.99
69	67.461	54.33		276.33
70	67.482	55.23		276.59
71	67.503	56.40		276.93
72	67.524	57.45		277.31
73	67.545	58.25		277.63
74	67.566	59.05		277.99
75	67.587	59.98		278.39
76	67.608	61.15		278.75
77	67.629	62.15		279.11
78	67.650	62.88		279.51
79	67.671	63.63		280.03
80	67.692	64.38		280.47

Table S3 List of samples and paleomagnetic directions details.

Mcd= meter of composite depth; Dec= declination (with respect the "x" axis of the core); Inc= Inclination; MAD= maximum angular deviation (*83*); N= number of interpolated vector end-points; AF 1 and 2= minimum and maximum field within which the paleomagnetic direction is isolated.

Leg	Site	Hole	Core	Type	Section	Half	Top (cm)	Bottom (cm)	depth mbsf	Dec (°)	Inc (°)	MAD (°)	N	AF1 (mT)	AF2 (mT)
74	528	*	32	R	4	W	86	88	412.19	322.1	52.6	1.6	12	20	100
74	528	*	32	R	4	W	97	99	412.30	312.4	66.6	1.2	12	20	100
74	528	*	32	R	4	W	109	111	412.42	9.7	54.2	2.4	12	20	100
74	528	*	32	R	4	W	118	120	412.51	10.3	50.8	3.8	12	20	100
74	528	*	32	R	5	W	9	11	412.92	106.3	17.2	2.7	10	20	80
74	528	*	32	R	5	W	20	22	413.03	162.5	20.9	6.1	9	20	70
74	528	*	32	R	5	W	29	31	413.12	45.6	-62.8	1.4	14	12	100
74	528	*	32	R	5	W	33	35	413.16	61.5	-50.3	1.9	15	9	100
74	528	*	32	R	5	W	52	54	413.35	158.3	-55.9	0.7	16	6	100
74	528	*	32	R	5	W	58	60	413.41	174.2	-48.9	1.1	16	6	100
74	528	*	32	R	5	W	67	69	413.50	160.8	-59.0	0.9	14	12	100
74	528	*	32	R	5	W	72	74	413.55	167.2	-52.6	0.7	15	9	100
208	1262	B	23	H	1	W	8	10	200.49	121.4	68.7	1.1	15	9	100
208	1262	B	23	H	1	W	21	23	200.62	128.2	23.7	2.4	10	9	50
208	1262	B	23	H	1	W	29	31	200.70	124.1	50.4	2.1	14	12	100
208	1262	B	23	H	1	W	40	42	200.81	154.5	18.8	11.7	11	15	80
208	1262	B	23	H	1	W	48	50	200.89	245.8	-49.3	7.2	9	20	70
208	1262	B	23	H	1	W	60	62	201.01	300.2	-51.9	2.3	16	6	100
208	1262	B	23	H	1	W	70	72	201.11	303.8	-37.1	1.6	15	9	100
208	1262	B	23	H	1	W	80	82	201.21	265.6	-59.8	1.6	14	12	100
208	1262	B	23	H	1	W	95	97	201.36	11.9	-77.5	3.1	15	9	100
208	1262	B	23	H	1	W	101	103	201.42	295.2	-58.4	2.3	16	6	100
208	1267	B	33	X	1	W	48	50	291.09	34.2	56.6	2.1	14	6	80
208	1267	B	33	X	1	W	60	62	291.21	18.7	44.7	3.0	14	9	90
208	1267	B	33	X	1	W	66	68	291.27	90.7	44.8	8.3	11	12	70
208	1267	B	33	X	1	W	80	82	291.41	54.2	-23.7	1.3	12	9	70
208	1267	B	33	X	1	W	88	90	291.49	82.7	56.6	2.2	15	9	100
208	1267	B	33	X	1	W	105	107	291.66	23.1	-55.8	3.4	13	9	80
208	1267	B	33	X	1	W	112	114	291.73	212.5	-74.5	3.0	8	12	45
208	1267	B	33	X	1	W	122	124	291.83	48.2	-62.5	1.8	8	20	60
208	1267	B	33	X	1	W	132	134	291.93	84.7	-38.0	1.7	13	6	70
208	1267	B	33	X	1	W	140	142	292.01	309.4	-55.7	1.8	16	6	100

Table S4a - Os from Deccan Trap

Stages	Duration (kyr)	Released Os from Deccan (t)	Required basalt fraction (%) to be weathered to supply unradiogenic Os				
			3 ppt Os	30 ppt Os	60 ppt Os	90 ppt Os	120 ppt Os
Pre-eruption	510						
1st drop	202	31522	242%	24%	12%	8%	6%
2nd drop	266	114091	874%	87%	44%	29%	22%
impact	8	3431	26%	3%	1%	1%	1%
Danian	214	91788	703%	70%	35%	23%	18%

Deccan Trap volume (extrusive)	1.50×10^{15}	m^3	
Basalt density	2.9	g/cm^3	
Total amount of extrusive basalt	4.4×10^{15}	ton	eruption rate (ton/kyr) : 6.3×10^{12}

Table S4b - Osmium box model basic results

	1st drop in $^{187}Os/^{188}Os$	2nd drop in $^{187}Os/^{188}Os$
Time (onset)	66.490	66.288
Drop in $^{187}Os/^{188}Os$	0.56 to 0.49	0.49 to 0.41
Drop in Δ $^{187}Os/^{188}Os$	0.07	0.08
Input unradiogenic Os required	~156 tons/kyr	~429 t/kyr
Duration of drop	66.490 to 66.288 Ma	66.288 Ma to K/Pg
Total Osmium input	31522 tons	114091 tons
Mean Os concentration Deccan Trap	90, 60 ppt	90, 60 ppt
Basalt required to be weathered	5.3×10^{14} tons (60 ppt)	1.9×10^{15} tons (60 ppt)
in tons	3.5×10^{14} tons (90 ppt)	1.3×10^{15} tons (90 ppt)
Basalt density	2.9 g/cm^3	2.9 g/cm^3
Basalt required to be weathered	1.8×10^{14} km^3 (60 ppt)	6.6×10^{14} km^3 (60 ppt)
in m^3	1.2×10^{14} km^3 (90 ppt)	4.4×10^{14} km^3 (90 ppt)
Total volume	% weathered	% weathered
Deccan Trap	from total	from total
1.5×10^6 m^3 (*90*)	12% (60 ppt)	44% (60 ppt)
	8% (90 ppt)	29% (90 ppt)
5×10^5 m^3 (*14*)	36% (60 ppt)	131% (60 ppt)
	24% (90 ppt)	87% (90 ppt)

Table S5 Age and magnitude of major late Maastrichtian Osmium isotope steps.

Event	$\Delta^{187}Os/^{188}Os$		Age Ma	$\delta^{13}C$		$\delta^{18}O$	
	1209	1262		1262	1209	1262	1209
Osmium isotope Step 1	0.04-0.06	0.05-0.06	66.49	-0.4 ‰	-0.3 ‰	-0.2 ‰	-0.1 ‰
Osmium isotope Step 2	0.04-0.05	0.04-0.06	66.28	-0.4 ‰	-0.4 ‰	-0.3 ‰	-0.5 ‰

Table S6 Age and magnitude of major late Maastrichtian benthic stable isotope steps.

Event	Age Ma	$\delta^{13}C$		$\delta^{18}O$	
		1262	1209	1262	1209
Late Maastrichtian Carbon Isotope Shift	66.33 - 66.29	+0.56‰	+0.80‰	+0.29‰	+0.30‰
Late Maastrichtian Warming	66.29 - 66.19	-0.60‰	-0.55‰	-0.83‰	-0.85‰
Late Maastrichtian Cooling	66.19 - 66.022	+0.90‰	+0.62‰	+0.86‰	+0.80‰

Table S7 Age of Deccan Eruptions near Osmium isotope steps.

Sample ID	Formation	Region	Age	error	Age Type	Source
Osmium Isotope Step 1			***66.49***			
RBCA*	Narmada	Malwa Plateau	66.492	± 0.07[&]	$^{206}Pb/^{238}U$	(*11*)
KAS15-3	Jawhar	Western Ghats	66.413	± 0.079[§]	$^{40}Ar/^{39}Ar$	(*13*)
KAS14-1	Jawhar	Western Ghats	66.380	± 0.05[$]	$^{40}Ar/^{39}Ar$	(*8*)
MAL19-2	Mandleshwar	Malwa Plateau	66.514	± 0.146[§]	$^{40}Ar/^{39}Ar$	(*30*)
MAL19-6	Mandleshwar	Malwa Plateau	66.549	± 0.082[§]	$^{40}Ar/^{39}Ar$	(*30*)
KH-275	Mandleshwar	Malwa Plateau	66.427	± 0.106[§]	$^{40}Ar/^{39}Ar$	(*30*)
SS-308	Mandleshwar	Malwa Plateau	66.393	± 0.118[§]	$^{40}Ar/^{39}Ar$	(*30*)
SM-1-302	Mandleshwar	Malwa Plateau	66.341	± 0.121[§]	$^{40}Ar/^{39}Ar$	(*30*)
Osmium Isotope Step 2			***66.28***			
RBCF	Narmada	Malwa Plateau	66.358	± 0.07[&]	$^{206}Pb/^{238}U$	(*11*)
RBBZ	Narmada	Malwa Plateau	66.312	± 0.06[&]	$^{206}Pb/^{238}U$	(*11*)
RBCI	Narmada	Malwa Plateau	66.275	± 0.06[&]	$^{206}Pb/^{238}U$	(*11*)
MAL19-1	Kalisindh	Malwa Plateau	66.35	± 0.072[§]	$^{40}Ar/^{39}Ar$	(*30*)
MAL19-5	Kalisindh	Malwa Plateau	66.272	± 0.120[§]	$^{40}Ar/^{39}Ar$	(*30*)
MAL19-3	Kalisindh	Malwa Plateau	66.348	± 0.132[§]	$^{40}Ar/^{39}Ar$	(*30*)
KAN-410	Kalisindh	Malwa Plateau	66.352	± 0.115[§]	$^{40}Ar/^{39}Ar$	(*30*)
KM20-1	Kankaria-Pirukheri	Malwa Plateau	66.351	± 0.103[§]	$^{40}Ar/^{39}Ar$	(*30*)
KS-497	Kankaria-Pirukheri	Malwa Plateau	66.349	± 0.150[§]	$^{40}Ar/^{39}Ar$	(*30*)
MAL19-4	Indore	Malwa Plateau	66.236	± 0.089[§]	$^{40}Ar/^{39}Ar$	(*30*)
KM20-2	Indore	Malwa Plateau	66.387	± 0.089[§]	$^{40}Ar/^{39}Ar$	(*30*)
GG-593	Indore	Malwa Plateau	66.242	± 0.113[§]	$^{40}Ar/^{39}Ar$	(*30*)
RBCJ2	Manpur	Malwa Plateau	66.256	± 0.04[&]	$^{206}Pb/^{238}U$	(*11*)
RBCL	Manpur	Malwa Plateau	66.250	± 0.05[&]	$^{206}Pb/^{238}U$	(*11*)
DEC13-30	Jawhar	Western Ghats	66.288	± 0.03[&]	$^{206}Pb/^{238}U$	(*14*)
KAS15-2	Jawhar	Western Ghats	66.234	± 0.093[§]	$^{40}Ar/^{39}Ar$	(*13*)
RBAB	Thakurvadi	Western Ghats	66.227	± 0.08[&]	$^{206}Pb/^{238}U$	(*14*)
RBCC[#]	Manpur	Malwa Plateau	66.320	± 0.42[&]	$^{206}Pb/^{238}U$	(*11*)
MSJ14-6	Igatpuri	Western Ghats	66.345	± 0.05[$]	$^{40}Ar/^{39}Ar$	(*8*)
MG7	Neral	Western Ghats	66.280	± 0.07[$]	$^{40}Ar/^{39}Ar$	(*8*)
BOR14-1	Thakurvadi	Western Ghats	66.270	± 0.07[$]	$^{40}Ar/^{39}Ar$	(*8*)
MG8C	Thakurvadi	Western Ghats	66.239	± 0.08[$]	$^{40}Ar/^{39}Ar$	(*8*)
MSJ14-4	Thakurvadi	Western Ghats	66.200	± 0.13[$]	$^{40}Ar/^{39}Ar$	(*8*)
MAT14-5	Bhimashankar	Western Ghats	66.210	±0.07[$]	$^{40}Ar/^{39}Ar$	(*8*)
felsic igneous complexes						
Girnar 9			66.369	0.03[&]	$^{206}Pb/^{238}U$	(33)
Phenai Mata 4-24			66.171	0.03[&]	$^{206}Pb/^{238}U$	(33)

Youngest zircon ages used here:

*RBCA vesicle rich flows in Mandu Ghat section within the lower-middle Narmada Fm. in the C30n magnetic polarity chron (*29*)

[#]RBCC Mandu Ghat section within the lowermost Manpur Fm. at the boundary between the C30n and C29r magnetic polarity chrons

[$]1σ uncertainty

[§]2σ uncertainty

[&]2σ uncertainty without systematic uncertainties

$^{40}Ar/^{39}Ar$ calibration after Renne et al. 2011 FC 28.294 Ma

Data S1-S7.

All data are open access in Pangaea https://doi.pangaea.de/10.1594/PANGAEA.964872

Data S1_DataSet_Shatsky_Deccan_XRF

Table S1a XRF1209 Hull and Norris 2011 Barium data on new depth scale
Table S1b XRF1209 Kim et al 2022 Barium data
Table S1c Splice of ODP Site 198-1209 XRF Barium data incl. age models
Table S1d XRF1210 Kim et al 2022 Barium data
Table S1e Splice of ODP Site 198-1210 XRF Barium data incl. age models
TableS1f XRF data DSDP Site 74-525A incl. age models.
TableS1g XRF data DSDP Site 74-527 incl. age models.
TableS1h XRF data DSDP Site 74-528 incl. age models.
TableS1i XRF data DSDP Hole 86-577B incl. age models.
TableS1j XRF data DSDP Site 86-577 incl. age models.
TableS1k Splice of DSDP Site 86 -577 incl. age models.
TableS1l XRF data ODP Site 208-1262 incl. age models.
TableS1m XRF data ODP Site 208-1267 incl. age models.

Data S2_DataSet_Shatsky_Deccan_ISO

Table S2a Benthic foraminifer carbonate carbon and oxygen isotopes of DSDP Hole 525A.
Table S2b Raw and species corrected benthic foraminifer data of ODP Site 1209.
Table S2c Generated raw and species corrected benthic foraminifer data of ODP Site 1209.
Table S2d Raw and species corrected benthic foraminifer data of ODP Site 1210.
Table S2e Raw and species corrected benthic foraminifer data of ODP Site 1262.
Table S2f Raw and species corrected benthic foraminifer data of ODP Site 1267.
Table S2g Bulk carbonate isotope data of DSDP Site 577.
Table S2h Bulk carbonate isotopes of ODP Site 1209.
Table S2i Bulk carbonate isotopes of ODP Site 1210.
Table S2j Bulk carbonate isotopes of ODP Site 1262.
Table S2k Bulk carbonate isotopes of ODP Site 1267.
Table S2l Coarse fractions > 63 µm of ODP Site 1209.
Table S2m Generated coarse fractions > 63 µm of ODP Site 1209.

Data S3_DataSet_Shatsky_Deccan_OSdata

Table S3a Osmium isotope data of DSDP Hole 525A.
Table S3b Osmium isotope data of DSDP Site 577.
Table S3c Osmium isotope data of ODP Hole 1209C.
Table S3d Generated Osmium isotope data of ODP Hole 1262B.
Table S3e Osmium and Rhenium isotope data of ODP Site 1209.
Table S3f Generated Osmium and Rhenium isotope data of ODP Site 1262.

Data S4_DataSet_Shatsky_Deccan_577_SPLICE
Table S4a Offsets of DSDP Site 577.
Table S4b Splice table of DSDP Site 577.
Table S4c Mapping pairs of DSDP Hole 577A.
Table S4d Mapping pairs of DSDP Hole 577B.
Table S4e Mapping pairs of DSDP Site 577.

Data S5_DataSet_Shatsky_Deccan_1209_SPLICE
Table S5a Offsets of ODP Site 1209.
Table S5b Splice table of ODP Site 1209.
Table S5c Mapping pairs of ODP Hole 1209A.
Table S5d Mapping pairs of ODP Hole 1209B.
Table S5e Mapping pairs of ODP Hole 1209C.

Data S6_DataSet_Shatsky_Deccan_PMag
Table S6a Magnetic inclination of DSDP Hole 525A.
Table S6b Magnetic inclination of DSDP Site 527.
Table S6c Magnetic inclination of DSDP Site 528.
Table S6d Magnetic inclination and declination of ODP Site 1262.
Table S6e Magnetic inclination and declination of ODP Site 1267.
Table S6f Natural remanent magnetization of DSDP 528, ODP 1262, and ODP 1267.

Data S7_DataSet_Shatsky_Deccan_CorrelationTie
Table S7a Correlation tie points to correlated DSDP Site 577 rmcd depth to ODP 1209 rmcd.
Table S7b Correlation tie points to correlated ODP Site 1210 rmcd depth to ODP 1209 rmcd.
Table S7c Correlation tie points to correlated ODP Site 1262 mcd depth to ODP 1267 rmcd.
Table S7d Correlation tie points to correlated DSDP Hole 525A mbsf depth to ODP 1267 rmcd.
Table S7e Correlation tie points to correlated DSDP Hole 527 mcd depth to ODP 1267 rmcd.
Table S7f Correlation tie points to correlated DSDP Hole 528 mcd depth to ODP 1267 rmcd.
Table S7g Correlation tie points to correlated ODP Site 1267 rmcd depth to ODP 1209 rmcd.

REFERENCES AND NOTES

1. J. Laskar, P. Robutel, F. Joutel, M. Gastineau, A. Correia, B. Levrard, A long-term numerical solution for the insolation quantities of the Earth. *Astron. Astrophys. Rev.* **428**, 261–285 (2004).

2. T. Westerhold, N. Marwan, A. J. Drury, D. Liebrand, C. Agnini, E. Anagnostou, J. S. K. Barnet, S. M. Bohaty, D. De Vleeschouwer, F. Florindo, T. Frederichs, D. A. Hodell, A. E. Holbourn, D. Kroon, V. Lauretano, K. Littler, L. J. Lourens, M. Lyle, H. Pälike, U. Röhl, J. Tian, R. H. Wilkens, P. A. Wilson, J. C. Zachos, An astronomically dated record of Earth's climate and its predictability over the last 66 million years. *Science* **369**, 1383–1387 (2020).

3. R. A. Berner, A model of atmospheric CO2 over Phanerozoic time. *Am. J. Sci.* **291**, 339–376 (1991).

4. J. Zachos, M. Pagani, L. Sloan, E. Thomas, K. Billups, Trends, rhythms, and aberrations in global climate 65 Ma to present. *Science* **292**, 686–693 (2001).

5. V. E. Courtillot, P. R. Renne, On the ages of flood basalt events. *Collect C. R. Geosci.* **335**, 113–140 (2003).

6. R. E. Ernst, D. P. G. Bond, S.–H. Zhang, K. L. Buchan, S. E. Grasby, N. Youbi, H. El Bilali, A. Bekker, L. S. Doucet, "Large igneous province record through time and implications for secular environmental changes and geological time-scale boundaries" in *Large Igneous Provinces: A Driver of Global Environmental and Biotic Changes*, Geophysical Monograph Series, R. E. Ernst, A. J. Dickson, A. Bekker, Eds. (AGU, 2020), pp. 1–26.

7. R. E. Ernst, in *Large Igneous Provinces* (Cambridge Univ. Press, 2014), pp. 545–640.

8. P. R. Renne, C. J. Sprain, M. A. Richards, S. Self, L. Vanderkluysen, K. Pande, State shift in Deccan volcanism at the Cretaceous-Paleogene boundary, possibly induced by impact. *Science* **350**, 76–78 (2015).

9. K. F. Kuiper, A. Deino, F. J. Hilgen, W. Krijgsman, P. R. Renne, J. R. Wijbrans, Synchronizing rock clocks of earth history. *Science* **320**, 500–504 (2008).

10. L. O'Connor, D. Szymanowski, M. P. Eddy, K. M. Samperton, B. Schoene, A red bole zircon record of cryptic silicic volcanism in the Deccan traps, India. *Geology* **50**, 460–464 (2022).

11. M. P. Eddy, B. Schoene, K. M. Samperton, G. Keller, T. Adatte, S. F. R. Khadri, U-Pb zircon age constraints on the earliest eruptions of the Deccan Large Igneous Province, Malwa Plateau, India. *Earth Planet. Sci. Lett.* **540**, 116249 (2020).

12. G. Keller, P. Mateo, J. Monkenbusch, N. Thibault, J. Punekar, J. E. Spangenberg, S. Abramovich, S. Ashckenazi-Polivoda, B. Schoene, M. P. Eddy, K. M. Samperton, S. F. R. Khadri, T. Adatte, Mercury linked to Deccan traps volcanism, climate change and the end-Cretaceous mass extinction. *Glob. Planet. Change* **194**, 103312 (2020).

13. C. J. Sprain, P. R. Renne, L. Vanderkluysen, K. Pande, S. Self, T. Mittal, The eruptive tempo of Deccan volcanism in relation to the Cretaceous-Paleogene boundary. *Science* **363**, 866–870 (2019).

14. B. Schoene, M. P. Eddy, K. M. Samperton, C. B. Keller, G. Keller, T. Adatte, S. F. R. Khadri, U-Pb constraints on pulsed eruption of the Deccan traps across the end-Cretaceous mass extinction. *Science* **363**, 862–866 (2019).

15. B. Schoene, K. M. Samperton, M. P. Eddy, G. Keller, T. Adatte, S. A. Bowring, S. F. R. Khadri, B. Gertsch, U-Pb geochronology of the Deccan traps and relation to the end-Cretaceous mass extinction. *Science* **347**, 182–184 (2015).

16. J. S. K. Barnet, K. Littler, D. Kroon, M. J. Leng, T. Westerhold, U. Röhl, J. C. Zachos, A new high-resolution chronology for the late Maastrichtian warming event: Establishing robust temporal links with the onset of Deccan volcanism. *Geology* **46**, 147–150 (2018).

17. B. Peucker-Ehrenbrink, G. E. Ravizza, in *Geologic Time Scale 2020*, F. M. Gradstein, J. G. Ogg, M. D. Schmitz, G. M. Ogg, Eds. (Elsevier, 2020), pp. 239–257.

18. B. Peucker-Ehrenbrink, G. Ravizza, The marine osmium isotope record. *Terra Nova* **12**, 205–219 (2000).

19. G. Ravizza, B. Peucker-Ehrenbrink, Chemostratigraphic evidence of Deccan volcanism from the marine osmium isotope record. *Science* **302**, 1392–1395 (2003).

20. B. Peucker-Ehrenbrink, G. Ravizza, A. W. Hofmann, The marine $^{187}Os/^{186}Os$ record of the past 80 million years. *Earth Planet. Sci. Lett.* **130**, 155–167 (1995).

21. N. Robinson, G. Ravizza, R. Coccioni, B. Peucker-Ehrenbrink, R. Norris, A high-resolution marine $^{187}Os/^{188}Os$ record for the late Maastrichtian: Distinguishing the chemical fingerprints of Deccan volcanism and the KP impact event. *Earth Planet. Sci. Lett.* **281**, 159–168 (2009).

22. T. D. Herbert, I. Premoli-Silva, E. Erba, A. G. Fischer, in *Geochronology*, *Time Scales and Global Stratigraphic Correlation*, W. A. Berggren, D. V. Kent, M. P. Aubry, J. Hardenbol, Eds. (SEPM, Special Publications, 1995), vol. 54, pp. 81–93.

23. S. J. Batenburg, M. Sprovieri, A. S. Gale, F. J. Hilgen, S. Hüsing, J. Laskar, D. Liebrand, F. Lirer, X. Orue-Etxebarria, N. Pelosi, J. Smit, Cyclostratigraphy and astronomical tuning of the Late Maastrichtian at Zumaia (Basque country, Northern Spain). *Earth Planet. Sci. Lett.* **359–360**, 264–278 (2012).

24. D. Husson, B. Galbrun, J. Laskar, L. A. Hinnov, N. Thibault, S. Gardin, R. E. Locklair, Astronomical calibration of the Maastrichtian (Late Cretaceous). *Earth Planet. Sci. Lett.* **305**, 328–340 (2011).

25. L. Li, G. Keller, Abrupt deep-sea warming at the end of the Cretaceous. *Geology* **26**, 995–998 (1998).

26. S. Schöbel, H. de Wall, M. Ganerød, M. K. Pandit, C. Rolf, Magnetostratigraphy and ^{40}Ar–^{39}Ar geochronology of the Malwa Plateau region (Northern Deccan traps), central western India: Significance and correlation with the main Deccan large igneous province sequences. *J. Asian Earth Sci.* **89**, 28–45 (2014).

27. A. J. Tholt, P. R. Renne, A. Marzoli, L. Vanderkluysen, D. Mohabey, B. Samant, A. Dhobale, K. Pande, S. Self, Geochronological constraints on the evolution and petrogenesis of the

malwa plateau subprovince of the Deccan traps. *Geochem. Geophys. Geosyst.* **24**, e2023GC011137 (2023).

28. S. Self, S. Blake, K. Sharma, M. Widdowson, S. Sephton, Sulfur and chlorine in late cretaceous Deccan Magmas and eruptive gas release. *Science* **319**, 1654–1657 (2008).

29. A. R. Basu, P. Chakrabarty, D. Szymanowski, M. Ibañez-Mejia, B. Schoene, N. Ghosh, R. B. Georg, Widespread silicic and alkaline magmatism synchronous with the Deccan traps flood basalts, India. *Earth Planet. Sci. Lett.* **552**, 116616 (2020).

30. S. Callegaro, D. R. Baker, P. R. Renne, L. Melluso, K. Geraki, M. J. Whitehouse, A. De Min, A. Marzoli, Recurring volcanic winters during the latest Cretaceous: Sulfur and fluorine budgets of Deccan traps lavas. *Sci. Adv.* **9**, eadg8284 (2023).

31. C. J. Sprain, P. R. Renne, W. A. Clemens, G. P. Wilson, Calibration of chron C29r: New high-precision geochronologic and paleomagnetic constraints from the Hell Creek region, Montana. *GSA Bull.* **130**, 1615–1644 (2018).

32. C. J. Allègre, J. L. Birck, F. Capmas, V. Courtillot, Age of the Deccan traps using ^{187}Re–^{187}Os systematics. *Earth Planet. Sci. Lett.* **170**, 197–204 (1999).

33. G. R. Heath, L. H. Burckle, A. E. D'Agostino, U. Bleil, K.–i. Horai, R. D. Jacobi, T. R. Janecek, I. Koizumi, L. A. Krissek, S. Monechi, N. Lenôtre, J. J. Morley, P. J. Schultheiss, A. A. Wright, *Initial Reports of the Deep Sea Drilling Project covering Leg 86 of the Cruises of the Drilling Vessel Glomar Challenger*, *Honolulu*, *Hawaii*, *to Yokohama*, *Japan*, *May-June 1982*, K. L. Turner, Ed. (Texas A&M University, Ocean Drilling Program, 1985).

34. Shipboard Scientific Party, Leg 198 summary, in T. J. Bralower, I. Premoli Silva,M. J. Malone, *et al.*, *Proc. ODP, Init. Repts.,* 198: College Station TX (Ocean Drilling Program, 2002), pp. 1–84.

35. T. C. Moore, Jr., *Initial reports of the Deep Sea Drilling Project covering Leg 74 of the Cruises of the Drilling Vessel Glomar Challenger; Cape Town, South Africa, to Walvis Bay, South Africa,*

June to July, 1980, J. H. Blakeslee, M. Lee, Eds. (Texas A&M University, Ocean Drilling Program, 1984).

36. J. C. Zachos, *Proceedings of the Ocean Drilling Program; Initial Reports; Early Cenozoic Extreme Climates; the Walvis Ridge Transect; Covering Leg 208 of the Cruises of the Drilling Vessel JOIDES Resolution; Rio de Janeiro*, *Brazil*, *to Rio de Janeiro*, *Brazil; Sites 1262-1267*, *6 March-6 May 2003*, H. Neville, K. Sherar, Eds. (Texas A&M University, Ocean Drilling Program, 2004).

37. T. Westerhold, U. Röhl, H. Pälike, R. Wilkens, P. A. Wilson, G. Acton, Orbitally tuned timescale and astronomical forcing in the middle Eocene to early Oligocene. *Clim. Past* **10**, 955–973 (2014).

38. T. Westerhold, U. Röhl, I. Raffi, E. Fornaciari, S. Monechi, V. Reale, J. Bowles, H. F. Evans, Astronomical calibration of the Paleocene time. *Palaeogeogr. Palaeoclimatol. Palaeoecol.* **257**, 377–403 (2008).

39. T. Westerhold, U. Röhl, J. Laskar, J. Bowles, I. Raffi, L. J. Lourens, J. C. Zachos, On the duration of magnetochrons C24r and C25n and the timing of early Eocene global warming events: Implications from the Ocean Drilling Program Leg 208 Walvis Ridge depth transect. *Paleoceanography* **22**, 10.1029/2006PA001322 (2007).

40. A. J. Drury, D. K. Kulhanek, T. Westerhold, A. C. Ravelo, G. Mountain, A. Holbourn,Y. Rosenthal, A. Schmitt, J. B. Wurtzel, J. Xu, R. Wilkens, U. Röhl, *Proceedings of the International Ocean Discovery Program*, *Volume 363: Western Pacific Warm Pool*,Y. Rosenthal, A. E. Holbourn, D. K. Kulhanek, Eds., the Expedition 363 Scientists (International Ocean Discovery Program, 2021).

41. A. J. Drury, T. Westerhold, D. Hodell, U. Röhl, Reinforcing the North Atlantic backbone: revision and extension of the composite splice at ODP Site 982. *Clim. Past* **14**, 321–338 (2018).

42. J. E. Kim, T. Westerhold, L. Alegret, A. J. Drury, U. Röhl, E. M. Griffith, Precessional pacing of tropical ocean carbon export during the Late Cretaceous. *Clim. Past* **18**, 2631–2641 (2022).

43. L. Li, G. Keller, Maastrichtian climate, productivity and faunal turnovers in planktic foraminifera in South Atlantic DSDP sites 525A and 21. *Mar. Micropaleontol.* **33**, 55–86 (1998).

44. N. J. Shackleton, M. A. Hall, U. Bleil, in *Init. Repts. DSDP, 86: Washington* (U.S. Govt. Printing Office), G. R. Heath, L. H. Burckle, et al., Eds. (1985), pp. 503-511.

45. E. I. Thompson, B. Schmitz, Barium and the late Paleocene d13C maximum: Evidence of increased marine surface productivity. *Paleoceanography* **12**, 239–254 (1997).

46. J. C. Zachos, M. A. Arthur, R. C. Thunell, D. F. Williams, E. J. Tappa, in *Initial Reports of the Deep Sea Drilling Project, Volume 86*, G. R. Heath, L. H. Burckle, Eds. (US Government Printing Office, 1985), pp. 513–532.

47. P. M. Hull, A. Bornemann, D. E. Penman, M. J. Henehan, R. D. Norris, P. A. Wilson, P. Blum, L. Alegret, S. J. Batenburg, P. R. Bown, T. J. Bralower, C. Cournede, A. Deutsch, B. Donner, O. Friedrich, S. Jehle, H. Kim, D. Kroon, P. C. Lippert, D. Loroch, I. Moebius, K. Moriya, D. J. Peppe, G. E. Ravizza, U. Röhl, J. D. Schueth, J. Sepúlveda, P. F. Sexton, E. C. Sibert, K. K. Śliwińska, R. E. Summons, E. Thomas, T. Westerhold, J. H. Whiteside, T. Yamaguchi, J. C. Zachos, On impact and volcanism across the Cretaceous-Paleogene boundary. *Science* **367**, 266–272 (2020).

48. T. Westerhold, U. Röhl, B. Donner, H. K. McCarren, J. C. Zachos, A complete high-resolution Paleocene benthic stable isotope record for the central Pacific (ODP Site 1209). *Paleoceanography* **26**, 10.1029/2010PA002092 (2011).

49. S. N. Dameron, R. M. Leckie, K. Clark, K. G. MacLeod, D. J. Thomas, J. A. Lees, Extinction, dissolution, and possible ocean acidification prior to the Cretaceous/Paleogene (K/Pg) boundary in the tropical Pacific. *Palaeogeogr. Palaeoclimatol. Palaeoecol.* **485**, 433–454 (2017).

50. L. Alegret, E. Thomas, K. C. Lohmann, End-Cretaceous marine mass extinction not caused by productivity collapse. *Proc. Natl. Acad. Sci. U.S.A.* **109**, 728–732 (2012).

51. C. Jung, S. Voigt, O. Friedrich, M. C. Koch, M. Frank, Campanian-Maastrichtian ocean circulation in the tropical Pacific. *Paleoceanography* **28**, 562–573 (2013).

52. C. Jung, S. Voigt, O. Friedrich, High-resolution carbon-isotope stratigraphy across the Campanian–Maastrichtian boundary at Shatsky Rise (tropical Pacific). *Cretac. Res.* **37**, 177–185 (2012).

53. J. S. K. Barnet, K. Littler, T. Westerhold, D. Kroon, M. J. Leng, I. Bailey, U. Röhl, J. C. Zachos, A high-fidelity benthic stable isotope record of late cretaceous–early Eocene climate change and carbon-cycling. *Paleoceanogr Paleoclimatol.* **34**, 672–691 (2019).

54. D. Kroon, J. C. Zachos, Leg 208 Scientific Party, in *Proceedings of the Ocean Drilling Program, Scientific Results*, *Volume 208*, D. Kroon, J. C. Zachos, C. Richter, Eds. (Ocean Drilling Program, 2007), pp. 1–55.

55. L. Alegret, E. Thomas, Benthic foraminifera across the Cretaceous/Paleogene boundary in the Southern Ocean (ODP Site 690): Diversity, food and carbonate saturation. *Mar. Micropaleontol.* **105**, 40–51 (2013).

56. G. Ravizza, D. VonderHaar, A geochemical clock in earliest Paleogene pelagic carbonates based on the impact-induced Os isotope excursion at the Cretaceous-Paleogene boundary. *Paleoceanography* **27**, PA3219 (2012).

57. M. L. G. Tejada, K. Suzuki, J. Kuroda, R. Coccioni, J. J. Mahoney, N. Ohkouchi, T. Sakamoto, Y. Tatsumi, Ontong Java Plateau eruption as a trigger for the early Aptian oceanic anoxic event. *Geology* **37**, 855–858 (2009).

58. J. Kuroda, R. S. Hori, K. Suzuki, D. R. Gröcke, N. Ohkouchi, Marine osmium isotope record across the Triassic-Jurassic boundary from a Pacific pelagic site. *Geology* **38**, 1095–1098 (2010).

59. S. B. Shirey, R. J. Walker, Carius tube digestion for low-blank rhenium-osmium analysis. *Anal. Chem.* **67**, 2136–2141 (1995).

60. D. G. Pearson, S. J. Woodland, Solvent extraction/anion exchange separation and determination of PGEs (Os, Ir, Pt, Pd, Ru) and Re–Os isotopes in geological samples by isotope dilution ICP-MS. *Chem. Geol.* **165**, 87–107 (2000).

61. A. S. Cohen, F. G. Waters, Separation of osmium from geological materials by solvent extraction for analysis by thermal ionisation mass spectrometry. *Anal. Chim. Acta* **332**, 269–275 (1996).

62. M. Roy-Barman, thesis, Paris, Université de Paris VII (1993).

63. M. I. Smoliar, R. J. Walker, J. W. Morgan, Re-Os ages of group IIA, IIIA, IVA, and IVB iron meteorites. *Science* **271**, 1099–1102 (1996).

64. G. R. Dickens, J. Backman, Core alignment and composite depth scale for the lower paleogene through uppermost cretaceous interval at deep sea drilling project site 577. *Newsl. Stratigr.* **46**, 47–68 (2013).

65. A. D. Chave, in *Initial Reports of the Deep Sea Drilling Project*, *Volume 74*, T. C. Moore,P. D. Rabinowitz, Eds. (US Government Printing Office, 1984), pp. 525–531.m.

66. J. Bowles, in *Proc. ODP, Sci. Results, 208: College Station, TX (Ocean Drilling Program),*D. Kroon, J. C. Zachos, C. Richter, Eds. (2006).

67. U. Bleil, in *Initial Reports of the Deep Sea Drilling Project, Volume 86*, G. R. Heath,L. H. Burckle, Eds. (US Government Printing Office, 1985), pp. 441–458.

68. P. M. Hull, R. D. Norris, Diverse patterns of ocean export productivity change across the Cretaceous-Paleogene boundary: New insights from biogenic barium. *Paleoceanography* **26**, PA3205 (2011).

69. A. Paytan, M. Kastner, F. P. Chavez, Glacial to interglacial fluctuations in productivity in the equatorial pacific as indicated by marine barite. *Science* **274**, 1355–1357 (1996).

70. M. Eagle, A. Paytan, K. R. Arrigo, G. van Dijken, R. W. Murray, A comparison between excess barium and barite as indicators of carbon export. *Paleoceanography* **18**, 10.1029/2002PA000793 (2003).

71. J. Laskar, M. Gastineau, J. B. Delisle, A. Farrés, A. Fienga, Strong chaos induced by close encounters with Ceres and Vesta. *Astronomy and Astrophysics* **532**, L4 (2011).

72. J. Laskar, in *Geologic Time Scale 2020*, F. M. Gradstein, J. G. Ogg, M. D. Schmitz, G. M. Ogg, Eds. (Elsevier, 2020), pp. 139–158.

73. J. Dinarès-Turell, T. Westerhold, V. Pujalte, U. Röhl, D. Kroon, Astronomical calibration of the Danian stage (Early Paleocene) revisited: Settling chronologies of sedimentary records across the Atlantic and Pacific Oceans. *Earth Planet. Sci. Lett.* **405**, 119–131 (2014).

74. N. J. Shackleton, and M. A. Hall, 1984 Oxygen and carbonisotope data from Leg 74 sediments. In *Init. Repts. DSDP, 74: Washington*, T. C. Moore Jr., P. D. Rabinowitz, et al., (U.S. Govt. Printing Office).

75. Shipboard Scientific Party, in *Proc. ODP, Init. Repts., 208: College Station, TX*, J. C. Zachos,D. Kroon, P. Blum, *et al.*, Eds. (Ocean Drilling Program, 2004), vol. 208, pp. 1–92.

76. Shipboard Scientific Party, in *Proc. ODP, Init. Repts., 208: College Station, TX*, J. C. Zachos,D. Kroon, P. Blum, et al., Eds. (Ocean Drilling Program, 2004), vol. 208, pp. 1–77.

77. J. D. A. Zijderveld, in *Methods in Paleomagnetism*, D. W. Collinson, K. M. Creer,S. K. Runcorn, Eds. (Elsevier, 1967), pp. 254–286.

78. P. C. Lurcock, G. S. Wilson, PuffinPlot: A versatile, user-friendly program for paleomagnetic analysis. *Geochem. Geophys. Geosyst.* **13**, Q06Z45 (2012).

79. D. Heslop, A. P. Roberts, Analyzing paleomagnetic data: To anchor or not to anchor?*J. Geophys. Res. Solid Earth* **121**, 7742–7753 (2016).

80. D. P. Maxbauer, J. M. Feinberg, D. L. Fox, M. A. X. UnMix, A web application for unmixing magnetic coercivity distributions. *Comput. Geosci.* **95**, 140–145 (2016).

81. R. Egli, Analysis of the field dependence of remanent magnetization curves. *J. Geophys. Res. Solid Earth* **108**, 2081 (2003).

82. M. J. Henehan, P. M. Hull, D. E. Penman, J. W. B. Rae, D. N. Schmidt, Biogeochemical significance of pelagic ecosystem function: An end-Cretaceous case study. *Philos. Trans. R. Soc. Lond. B Biol. Sci.* **371**, 20150510 (2016).

83. E. Tanaka, K. Yasukawa, J. Ohta, Y. Kato, Enhanced continental chemical weathering during the multiple early Eocene hyperthermals: New constraints from the southern Indian Ocean. *Geochim. Cosmochim. Acta* **331**, 192–211 (2022).

84. H. Sato, T. Nozaki, T. Onoue, A. Ishikawa, K. Soda, K. Yasukawa, J.–I. Kimura, Q. Chang, Y. Kato, M. Rigo, Rhenium-osmium isotope evidence for the onset of volcanism in the central Panthalassa Ocean during the Norian "chaotic carbon episode". *Glob. Planet. Change* **229**, 104239 (2023).

85. S. Levasseur, J. L. Birck, C. J. Allègre, The osmium riverine flux and the oceanic mass balance of osmium. *Earth Planet. Sci. Lett.* **174**, 7–23 (1999).

86. R. J. Walker, J. W. Morgan, Rhenium-osmium isotope systematics of carbonaceous chondrites. *Science* **243**, 519–522 (1989).

87. D. Stüben, U. Kramar, Z. A. Berner, M. Meudt, G. Keller, S. Abramovich, T. Adatte, U. Hambach, W. Stinnesbeck, Late Maastrichtian paleoclimatic and paleoceanographic changes inferred from Sr/Ca ratio and stable isotopes. *Palaeogeogr. Palaeoclimatol. Palaeoecol.* **199**, 107–127 (2003).

88. D. Stüben, U. Kramar, Z. Berner, W. Stinnesbeck, G. Keller, T. Adatte, Trace elements, stable isotopes, and clay mineralogy of the Elles II K–T boundary section in Tunisia: indications for sea level fluctuations and primary productivity. *Palaeogeogr. Palaeoclimatol. Palaeoecol.* **178**, 321–345 (2002).

89. G. P. Wilson, in *Through the End of the Cretaceous in the Type Locality of the Hell Creek Formation in Montana and Adjacent Areas*, G. P. Wilson, W. A. Clemens, J. R. Horner,J. H. Hartman, Eds. (Geological Society of America, 2014).

90. P. R. Renne, R. Mundil, G. Balco, K. Min, K. R. Ludwig, Joint determination of ^{40}K decay constants and ^{40}Ar/^{40}K for the Fish Canyon sanidine standard, and improved accuracy for ^{40}Ar/^{39}Ar geochronology. *Geochim. Cosmochim. Acta* **74**, 5349–5367 (2010).

91. R. Sutherland, G. R. Dickens, P. Blum, the Expedition 371 Scientists, *Proceedings of the International Ocean Discovery Program; Tasman Frontier Subduction Initiation and Paleogene Climate; Expedition 371 of the R/V JOIDES Resolution*, *Townsville*, *Australia*, *to Hobart*, *Australia; Sites U1506-U1511*, *27 July-26 September 2017* (International Ocean Discovery Program, 2019).

92. J. L. Kirschvink, The least-squares line and plane and the analysis of paleomagnetic data. *Geophys. J. Roy. Astron. Soc.* **62**, 699–718 (1980).

93. W. C. Clyde, J. Ramezani, K. R. Johnson, S. A. Bowring, M. M. Jones, Direct high-precision U–Pb geochronology of the end-Cretaceous extinction and calibration of Paleocene astronomical timescales. *Earth Planet. Sci. Lett.* **452**, 272–280 (2016).

94. S. Levasseur, J.–L. Birck, C. J. Allègre, Direct measurement of femtomoles of osmium and the ^{187}Os/^{186}Os ratio in seawater. *Science* **282**, 272–274 (1998).

95. M. Sharma, in *Encyclopedia of Ocean Sciences*, J. K. Cochran, H. J. Bokuniewicz, P. L. Yager, Eds. (Academic Press, 2019), pp. 174–180.

96. C. J. Allègre, J.–M. Luck, Osmium isotopes as petrogenetic and geological tracers. *Earth Planet. Sci. Lett.* **48**, 148–154 (1980).

97. M. F. Coffin, O. Eldholm, Large igneous provinces: Crustal structure, dimensions, and external consequences. *Rev. Geophys.* **32**, 1–36 (1994).

98. M. Heřmanská, M. J. Voigt, C. Marieni, J. Declercq, E. H. Oelkers, A comprehensive and internally consistent mineral dissolution rate database: Part I: Primary silicate minerals and glasses. *Chem. Geol.* **597**, 120807 (2022).

99. C. Dessert, B. Dupré, L. M. François, J. Schott, J. Gaillardet, G. Chakrapani, S. Bajpai, Erosion of Deccan traps determined by river geochemistry: Impact on the global climate and the $^{87}Sr/^{86}Sr$ ratio of seawater. *Earth Planet. Sci. Lett.* **188**, 459–474 (2001).

100. C. Dessert, B. Dupré, J. Gaillardet, L. M. François, C. J. Allègre, Basalt weathering laws and the impact of basalt weathering on the global carbon cycle. *Chem. Geol.* **202**, 257–273 (2003).

101. U. Krähenbühl, M. Geissbühler, F. Bühler, P. Eberhardt, D. L. Finnegan, Osmium isotopes in the aerosols of the mantle volcano Mauna Loa. *Earth Planet. Sci. Lett.* **110**, 95–98 (1992).

102. J. Wimpenny, A. Gannoun, K. W. Burton, M. Widdowson, R. H. James, S. R. Gíslason, Rhenium and osmium isotope and elemental behaviour accompanying laterite formation in the Deccan region of India. *Earth Planet. Sci. Lett.* **261**, 239–258 (2007).

103. M. Sinnesael, L. M. E. Percival, T. Schulz, J. Vellekoop, S. Goderis, K. Daems, Y. Gao, M. Leermakers, A. Montanari, R. Coccioni, C. Koeberl, P. Claeys, Deep marine records of Deccan Trap volcanism before the Cretaceous–Paleogene (K–Pg) mass extinction. *GSA Bull.* 10.1130/B37446.1 (2024).

104. G. Ravizza, R. N. Norris, J. Blusztajn, M. P. Aubry, An osmium isotope excursion associated with the late paleocene thermal maximum: Evidence of intensified chemical weathering. *Paleoceanography* **16**, 155–163 (2001).

105. A. J. van Velzen, M. J. Dekkers, Low-temperature oxidation of magnetite in loess-paleosol sequences: A correction of rock magnetic parameters. *Studia Geophysica et Geodaetica* **43**, 357–375 (1999).

106. S. Cisowski, Interacting vs. non-interacting single domain behavior in natural and synthetic samples. *Phys. Earth Planet. Interiors* **26**, 56–62 (1981).

107. P. Jaqueto, R. I. F. Trindade, G. A. Hartmann, V. F. Novello, F. W. Cruz, I. Karmann, B. E. Strauss, J. M. Feinberg, Linking speleothem and soil magnetism in the Pau d'Alho cave (central South America). *J. Geophys. Res. Solid Earth* **121**, 7024–7039 (2016).

108. M. Fuller, Y. Touchard, in *The Cenozoic Southern Ocean: Tectonics*, *Sedimentation and Climate Change between Australia and Antarctica*, Geophysical Monograph Series,N. F. Exon, J. P. Kennett, M. J. Malone, Eds. (AGU, 2004), vol. 151, pp. 63–78.

109. B. S. Singer, B. R. Jicha, N. Mochizuki, R. S. Coe, Synchronizing volcanic, sedimentary, and ice core records of Earth's last magnetic polarity reversal. *Sci. Adv.* **5**, eaaw4621 (2019).

110. E. Dallanave, C. Agnini, G. Muttoni, D. Rio, Paleocene magneto-biostratigraphy and climate-controlled rock magnetism from the Belluno Basin, Tethys Ocean, Italy. *Palaeogeogr. Palaeoclimatol. Palaeoecol.* **337–338**, 130–142 (2012).

111. W. Alvarez, M. A. Arthur, A. G. Fischer, W. Lowrie, G. Napoleone, I. P. Silva, W. M. Roggenthen, Upper Cretaceous-Paleocene magnetic stratigraphy at Gubbio, Italy V. Type section for the Late Cretaceous-Paleocene geomagnetic reversal time scale. *Geol. Soc. Am. Bull.* **88**, 383–389 (1977).

112. H. Chauris, J. LeRousseau, B. Beaudoin, S. Propson, A. Montanari, Inoceramid extinction in the Gubbio basin (northeastern Apennines of Italy) and relations with mid–Maastrichtian environmental changes. *Palaeogeogr. Palaeoclimatol. Palaeoecol.* **139**, 177–193 (1998).

113. A. Montanari, C. Koeberl, *Impact Stratigraphy. The Italian Record*, Lecture Notes in Earth Sciences Series (Springer-Verlag, 2000), vol. 93, pp. 364.

114. R. Coccioni, I. Premoli Silva, Revised Upper Albian–Maastrichtian planktonic foraminiferal biostratigraphy and magneto-stratigraphy of the classical Tethyan Gubbio section (Italy). *Newsl. Stratigr.* **48**, 47–90 (2015).

115. J. G. Ogg, in *Geologic Time Scale 2020*, F. M. Gradstein, J. G. Ogg, M. D. Schmitz, G. M. Ogg, Eds. (Elsevier, 2020), pp. 159–192.

116. N. Thibault, D. Husson, R. Harlou, S. Gardin, B. Galbrun, E. Huret, F. Minoletti, Astronomical calibration of upper Campanian–Maastrichtian carbon isotope events and calcareous plankton biostratigraphy in the Indian Ocean (ODP Hole 762C): Implication for the age of the Campanian–Maastrichtian boundary. *Palaeogeogr. Palaeoclimatol. Palaeoecol.* **337–338**, 52–71 (2012).

117. S. J. Batenburg, A. S. Gale, M. Sprovieri, F. J. Hilgen, N. Thibault, M. Boussaha, X. Orue-Etxebarria, An astronomical time scale for the Maastrichtian based on the Zumaia and Sopelana sections (Basque country, northern Spain). *J. Geol. Soc. London* **171**, 165–180 (2014).

118. N. Thibault, S. Gardin, B. Galbrun, Latitudinal migration of calcareous nannofossil Micula murus in the Maastrichtian: Implications for global climate change. *Geology* **38**, 203–206 (2010).

119. S. Gardin, B. Galbrun, N. Thibault, R. Coccioni, I. Premoli Silva, Bio-magnetochronology for the upper Campanian–Maastrichtian from the Gubbio area, Italy: new results from the Contessa Highway and Bottaccione sections. *Newsl. Stratigr.* **45**, 75–103 (2012).

120. P. Zhu, J. D. Macdougall, Calcium isotopes in the marine environment and the oceanic calcium cycle. *Geochim. Cosmochim. Acta* **62**, 1691–1698 (1998).

121. C. L. De La Rocha, D. J. DePaolo, Isotopic evidence for variations in the marine calcium cycle over the cenozoic. *Science* **289**, 1176–1178 (2000).

122. N. Gussone, A.–S. C. Ahm, K. V. Lau, H. J. Bradbury, Calcium isotopes in deep time: Potential and limitations. *Chem. Geol.* **544**, 119601 (2020).

123. B. J. Linzmeier, A. D. Jacobson, B. B. Sageman, M. T. Hurtgen, M. E. Ankney, S. V. Petersen, T. S. Tobin, G. D. Kitch, J. Wang, Calcium isotope evidence for environmental variability before and across the Cretaceous-Paleogene mass extinction. *Geology* **48**, 34–38 (2020).

124. A. Jouini, G. Paris, G. Caro, A. Bartolini, S. Gardin, Constraining oceanic carbonate chemistry evolution during the Cretaceous-Paleogene transition: Combined benthic and planktonic calcium isotope records from the equatorial Pacific Ocean. *Earth Planet. Sci. Lett.* **619**, 118305 (2023).

125. J. Tang, M. Dietzel, F. Böhm, S. J. Köhler, A. Eisenhauer, Sr2+/Ca2+ and 44Ca/40Ca fractionation during inorganic calcite formation: II. Ca isotopes. *Geochim. Cosmochim. Acta* **72**, 3733–3745 (2008).

126. N. Gussone, G. Langer, M. Geisen, B. A. Steel, U. Riebesell, Calcium isotope fractionation in coccoliths of cultured Calcidiscus leptoporus, Helicosphaera carteri, Syracosphaera pulchra and Umbilicosphaera foliosa. *Earth Planet. Sci. Lett.* **260**, 505–515 (2007).

127. C. Holmden, D. A. Papanastassiou, P. Blanchon, S. Evans, δ44/40Ca variability in shallow water carbonates and the impact of submarine groundwater discharge on Ca-cycling in marine environments. *Geochim. Cosmochim. Acta* **83**, 179–194 (2012).

128. N. Gussone, H. L. Filipsson, H. Kuhnert, Mg/Ca, Sr/Ca and Ca isotope ratios in benthonic foraminifers related to test structure, mineralogy and environmental controls. *Geochim. Cosmochim. Acta* **173**, 142–159 (2016).

129. R. D. Müller, M. Seton, S. Zahirovic, S. E. Williams, K. J. Matthews, N. M. Wright, G. E. Shephard, K. T. Maloney, N. Barnett-Moore, M. Hosseinpour, D. J. Bower, J. Cannon, Ocean basin evolution and global-scale plate reorganization events since pangea breakup. *Annu. Rev. Earth Planet. Sci.* **44**, 107–138 (2016).

130. W. Cao, S. Zahirovic, N. Flament, S. Williams, J. Golonka, R. D. Müller, Improving global paleogeography since the late Paleozoic using paleobiology. *Biogeosciences* **14**, 5425–5439 (2017).

131. J. F. Diehl, M. E. Beck Jr., S. Beske-Diehl, D. Jacobson, B. C. Hearn Jr., Paleomagnetism of the late cretaceous-early tertiary North-central Montana Alkalic province. *J. Geophys. Res. Solid Earth* **88**, 10593–10609 (1983).

132. J. T. Hagstrum, P. W. Lipman, R. B. Moore, Late Cretaceous paleomagnetism of the Tombstone district and vicinity: Evidence for a rotational domain boundary in southeastern Arizona. *Tectonics* **13**, 1295–1308 (1994).

133. C. R. Montes-Lauar, I. G. Pacca, A. J. Melfi, K. Kawashita, Late Cretaceous alkaline complexes, southeastern Brazil: Paleomagnetism and geochronology. *Earth Planet. Sci. Lett.* **134**, 425–440 (1995).

134. M. Ganerød, M. A. Smethurst, T. H. Torsvik, T. Prestvik, S. Rousse, C. McKenna, D. J. J. Van Hinsbergen, B. W. H. Hendriks, The North Atlantic Igneous Province reconstructed and its relation to the Plume Generation Zone: The Antrim Lava Group revisited. *Geophys. J. Int.* **182**, 183–202 (2010).

135. A. E. Jay, C. M. Niocaill, M. Widdowson, S. Self, W. Turner, New palaeomagnetic data from the Mahabaleshwar Plateau, Deccan Flood Basalt Province, India: Implications for the volcanostratigraphic architecture of continental flood basalt provinces. *J. Geol. Soc. London* **166**, 13–24 (2009).

136. D. Vandamme, V. Courtillot, J. Besse, R. Montigny, Paleomagnetism and age determinations of the Deccan traps (India): Results of a Nagpur-Bombay Traverse and review of earlier work. *Rev. Geophys.* **29**, 159–190 (1991).

137. J. N. Prasad, S. K. Patil, P. D. Saraf, M. Venkateshwarlu, D. R. K. Rao, Palaeomagnetism of Dyke Swarms from the Deccan Volcanic Province of India. *J. Geomag. Geoelec.* **48**, 977–991 (1996).

138. R. A. Fisher, Dispersion on a sphere. *Proc. R. Soc. Lond. A Math. Phys. Sci.* **217**, 295–305 (1953).

139. R. D. Müller, J. Cannon, X. Qin, R. J. Watson, M. Gurnis, S. Williams, T. Pfaffelmoser, M. Seton, S. H. J. Russell, S. Zahirovic, GPlates: Building a virtual Earth through deep time. *Geochem. Geophys. Geosyst.* **19**, 2243–2261 (2018).
